# Novel Drug Candidates Improve Ganglioside Accumulation and Neural Dysfunction in GM1 Gangliosidosis Models with Autophagy Activation

**DOI:** 10.1016/j.stemcr.2020.03.012

**Published:** 2020-04-16

**Authors:** Ryutaro Kajihara, Tadahiro Numakawa, Haruki Odaka, Yuji Yaginuma, Noemi Fusaki, Toshika Okumiya, Hirokazu Furuya, Seiji Inui, Takumi Era

**Affiliations:** 1 Department of Cell Modulation, Institute of Molecular Embryology and Genetics, Kumamoto University, 2-2-1 Honjo, Chuo-ku, Kumamoto 860-0811, Japan; 2 Department of Biomedical Laboratory Sciences, Faculty of Life Sciences, Kumamoto University, Kumamoto 862-0976, Japan; 3 Department of Morphological and Physiological Sciences, Faculty of Life Sciences, Kumamoto University, Kumamoto 862-0976, Japan; 4 University Research Administration Center, Office of Research Promotion, Tohoku University, Tohoku 980-8577, Japan; 5 Department of Neurology, Kochi Medical School, Kochi University, Kochi 783-8505, Japan

**Keywords:** induced pluripotent stem cells, lysosomal storage disease, GM1 gangliosidosis, drug development

## Abstract

GM1 gangliosidosis is a lysosomal storage disease caused by loss of lysosomal β-galactosidase activity and characterized by progressive neurodegeneration due to massive accumulation of GM1 ganglioside in the brain. Here, we generated induced pluripotent stem cells (iPSCs) derived from patients with GM1 gangliosidosis, and the resultant neurons showed impaired neurotransmitter release as a presynaptic function and accumulation of GM1 ganglioside. Treatment of normal neurons with GM1 ganglioside also disturbed presynaptic function. A high-content drug-screening system was then established and identified two compounds as drug candidates for GM1 gangliosidosis. Treatment of the patient-derived neurons with the candidate agents activated autophagy pathways, reducing GM1 ganglioside accumulation *in vitro* and *in vivo*, and restoring the presynaptic dysfunction. Our findings thus demonstrated the potential value of patient-derived iPSC lines as cellular models of GM1 gangliosidosis and revealed two potential therapeutic agents for future clinical application.

## Highlights

• GM1 ganglioside accumulation affects presynaptic function in normal neurons
• Presynaptic function is impaired in neurons from GM1 gangliosidosis iPSCs
• The drugs candidates are identified using GM1 gangliosidosis iPSCs
• The drug candidates activate both autophagy and sphingolipid degradation

## Graphical Abstract

**Figure:**
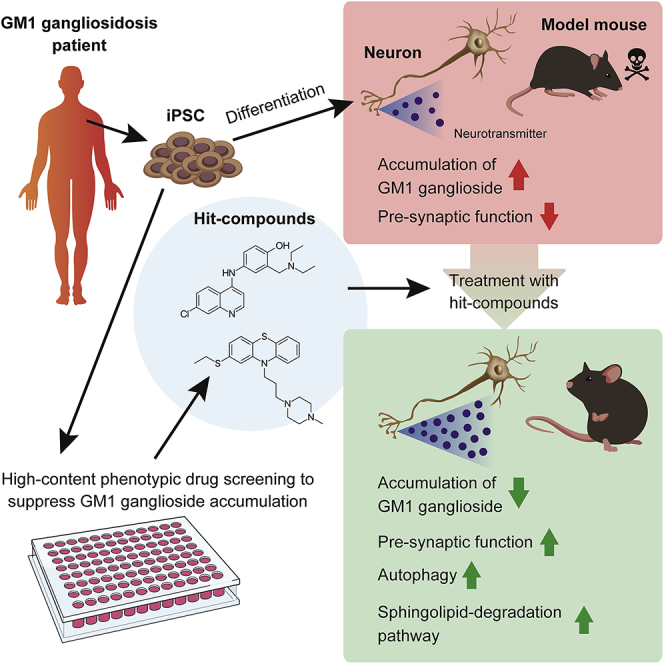

## Teaser

GM1 gangliosidosis is a lysosomal storage disease characterized by neurodegeneration due to massive accumulation of GM1 ganglioside in the brain. In this article, Era and his colleagues generate iPSCs from patients and find presynaptic dysfunction in neurons from GM1 gangliosidosis iPSCs. They identify two potential drug candidates that can restore the presynaptic dysfunction and reduce *in vivo* GM1 ganglioside accumulation.

## Introduction

GM1 gangliosidosis is a lysosomal storage disorder characterized by abnormal accumulation of GM1 ganglioside. The main clinical feature of the disease is neural dysfunction due to massive GM1 ganglioside deposition in the central nervous system (CNS) (Sandhoff and Harzer, 2013). This abnormal deposition is caused by a deficiency in lysosomal β-galactosidase (β-GAL) activity that limits the patients' ability to degrade GM1 ganglioside in lysosomes and eventually leads to excessive GM1 ganglioside accumulation, particularly in the brain. While it remains unclear how this abnormal storage of GM1 ganglioside directly affects neural function, previous studies proposed several pathological pathways, including the unfolded protein response (UPR), endoplasmic reticulum calcium signaling, autophagy, and inflammasome activation (Sano et al., 2009, Son et al., 2015, Takamura et al., 2008, Tessitore et al., 2004).

Depending on the age of onset, GM1gangliosidosis is classified into three forms: infantile, juvenile, and adult forms. The life prognosis is poorer with early onset than with late onset. The infantile form develops by the age of 6 months and exhibits the most severe symptoms, such as developmental regression, seizures, eye abnormality, and hepatosplenomegaly. The juvenile form develops after 5 years and exhibits developmental regression. The adult form has milder symptoms such as dystonia and progresses chronically (Sandhoff and Harzer, 2013).

Several therapeutic approaches have been tried to treat the disease, such as bone marrow transplantation, gene therapy, substrate reduction therapy, and chemical chaperone therapy (Baek et al., 2010, Elliot-Smith et al., 2008, Shield et al., 2005, Suzuki et al., 2012); however, these strategies have had limited effects, and there remains no effective treatment or candidate drugs on the horizon for GM1 gangliosidosis patients. Disease models in mice and using patient-derived fibroblasts have been developed to understand the pathogenesis of GM1 gangliosidosis (Sano et al., 2009, Tessitore et al., 2004); however, efforts to generate human neural cells able to simulate the disease have proven unsuccessful. For instance, human infantile GM1 gangliosidosis exhibits early onset and poor prognosis; it develops at 0–6 months of age and patients die around the age of 1 year (Brunetti-Pierri and Scaglia, 2008), while β-GAL null mice (BKO mice), a popular mouse model for GM1 gangliosidosis, develop disease around 4 months of age and live up to 10 months of age, corresponding to over 20 years of age in humans (Matsuda et al., 1997). In addition, unlike human infantile patients, hepatosplenomegaly and skeletal dysplasia are not observed in BKO mice (Matsuda et al., 1997). Thus, the mouse model also does not exactly recapitulate the human disease.

Induced pluripotent stem cells (iPSCs) are artificially generated from human somatic cells with overexpression of four reprogramming factors: OCT3/4, SOX2, KLF4, and cMYC (Takahashi et al., 2007). Human iPSCs can easily proliferate and have the potential to generate various cell types *in vitro*. Based on these attributes, patient-derived iPSCs have proven powerful tools in establishing human disease models, pathological research, and new drug development (Latour et al., 2019, Soga et al., 2015).

The present study sought to elucidate the pathological mechanisms of neural dysfunction in GM1 gangliosidosis and, based on the results, to identify therapeutic candidates. To approach this aim, we generated patient-derived iPSCs, characterized the impaired function of neural cells differentiated from the iPSCs, and established a high-content drug-screening system.

## Results

### Establishment of iPSC Lines from GM1 Gangliosidosis Patients

To investigate possible pathological events and drug targets in GM1 gangliosidosis, we generated iPSCs (GM1-iPSCs) from skin fibroblasts of three patients carrying homozygous mutations in β-GAL, R201C in A138 (juvenile form) and A360 (juvenile form), and I51T in A154 (adult form) (Figure S1A). A previous study showed that they were causative mutations in GM1 gangliosidosis (Morita et al., 2009). Six clonal iPSC lines established from three affected patients (1-1 and 1-3 from patient A138, 2-19 and 4-21 from patient A154, and 1-3 and 1-4 from patient A360) exhibited embryonic stem cell-like morphology and expressed a set of pluripotent markers determined by immunostaining and PCR analyses (Figures S1B and S1C). Subsequent examination of the iPSCs for differentiation capacity using a teratoma formation assay and histological analysis revealed descendants of all three germ cell layers, including columnar epithelia, melanin pigment-containing cells, cartilage, muscle, and various glandular structures (Figure S1D). We also confirmed that the iPSC lines derived from patients A138 and A360 had a normal karyotype of 46XX, whereas those from patient A154 (both 2-19 and 4-21) showed an abnormal karyotype of 46XX, add(9) (p24, arrow), and the abnormal karyotypes can affect the results in further experiments (Figure S1E). We therefore mainly used the A138 iPSC line and confirmed the results using A154 or A360 iPSC lines. The A154 patient was diagnosed as a different clinical form with a different mutation in the *GLB1* gene, which encodes β-GAL protein, from other two. Thus, using the A154-derived iPSCs, we sometimes confirmed the results from other iPSCs. In this study, 201B7 and 409B2 iPSC lines were used as normal controls (Okita et al., 2011, Takahashi et al., 2007).

### GM Gangliosides Accumulated in NSCs Derived from GM1-iPSCs

One of the main clinical features reported in GM1 gangliosidosis is neurodegeneration (Brunetti-Pierri and Scaglia, 2008, Sandhoff and Harzer, 2013), implicating neural cells, including neural stem cells (NSCs) and differentiated neurons as a profoundly affected cell population in the disease. To assess the cellular phenotypes present in GM1 gangliosidosis, we first differentiated the disease-derived iPSC lines into NSCs expressing specific markers, SOX1, PAX6, and NESTIN (Figure S1F). Then, we examined possible differences in neural induction efficiency between disease and control (201B7 and 409B2) iPSCs by comparing mRNA expression levels of NSC markers (*SOX1*, *ZNF521*, and *PAX6*) by qRT-PCR analysis, but found no differences between the disease and control cells (Figure S1G). Both control and disease NSCs also showed downregulation of *NANOG*, an iPSC marker (Figure S1G).

**Figure 1:**
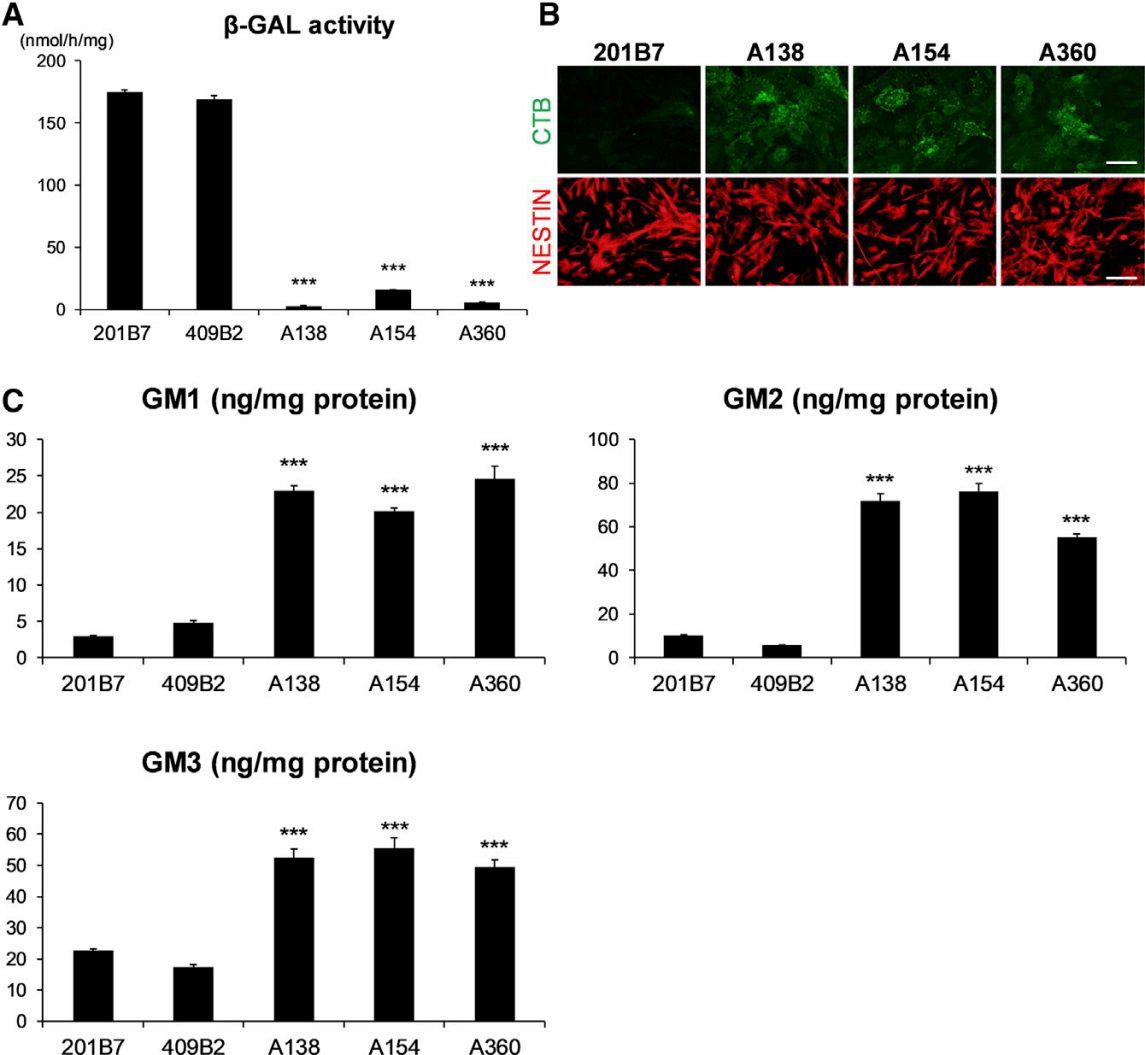
Biochemical Phenotype of GM1-iPSC-Derived Neural Stem Cells (A) β-GAL activities in normal and GM1 gangliosidosis-derived NSCs. The NSCs were generated from disease-derived iPSCs (A138 1-3, A154 4-21, and A360 1-3) and normal iPSCs (201B7). The NSC lysates were incubated with artificial β-GAL substrate (4-methylumbelliferyl-β-D-galactoside), and enzyme activities were measured based on fluorescence intensities. The activities are expressed as nmol/h/mg protein. The bars represent the mean ± SD from three independent experiments. ^∗∗∗^p < 0.001, disease versus normal, Student's t test. (B) CTB staining of iPSC-derived NSCs. Control (201B7) and disease (A138 1-3, A154 4-21, and A360 1-3)-derived NSCs were also stained with anti-NESTIN antibody (red color) followed by Alexa Fluor 488-CTB (green color). Scale bars, 50 μm. (C) Accumulation of GM gangliosides in iPSC-derived NSCs. Total amounts of GM1, GM2, and GM3 gangliosides were measured by LC-MS. The bars represent the mean ± SD from three independent experiments. ^∗∗∗^p < 0.001, disease versus normal, Student's t test.

We next investigated whether the patient-derived NSCs could mimic a disease-specific phenotype. In GM1 gangliosidosis, deficient lysosomal β-GAL activity due to a mutation in the *GLB1* gene causes accumulation of GM1 ganglioside. We therefore used an artificial β-GAL substrate (4-methylumbelliferyl-β-D-galactoside) to assess the enzymatic activities of both normal and disease-derived NSCs. GM1 gangliosidosis-derived NSCs showed markedly lower β-GAL activity compared with control NSCs (Figure 1A). We also visualized the intracellular GM1 ganglioside make-up in the NSCs using Alexa Fluor 488-conjugated cholera toxin subunit-B (AF488-CTB), which specifically binds to the GM1 ganglioside (Iwamasa et al., 1987). Positive staining was negligible in the control NSCs, whereas the disease-derived NSCs showed a strong signal for AF488-CTB (Figures 1B and S1H), and liquid chromatography-mass spectrometry (LC-MS) analysis confirmed the increased levels of GM1 ganglioside (Figure 1C). Interestingly, GM2 and GM3 gangliosides were also accumulated in the disease-derived NSCs (Figure 1C), and together, these results suggest that the NSCs derived from GM1-iPSCs mirror the biochemical phenotype of GM1 gangliosidosis.

### Accumulation of GM1 Ganglioside Disturbs Presynaptic Function *In Vitro*

**Figure 2:**
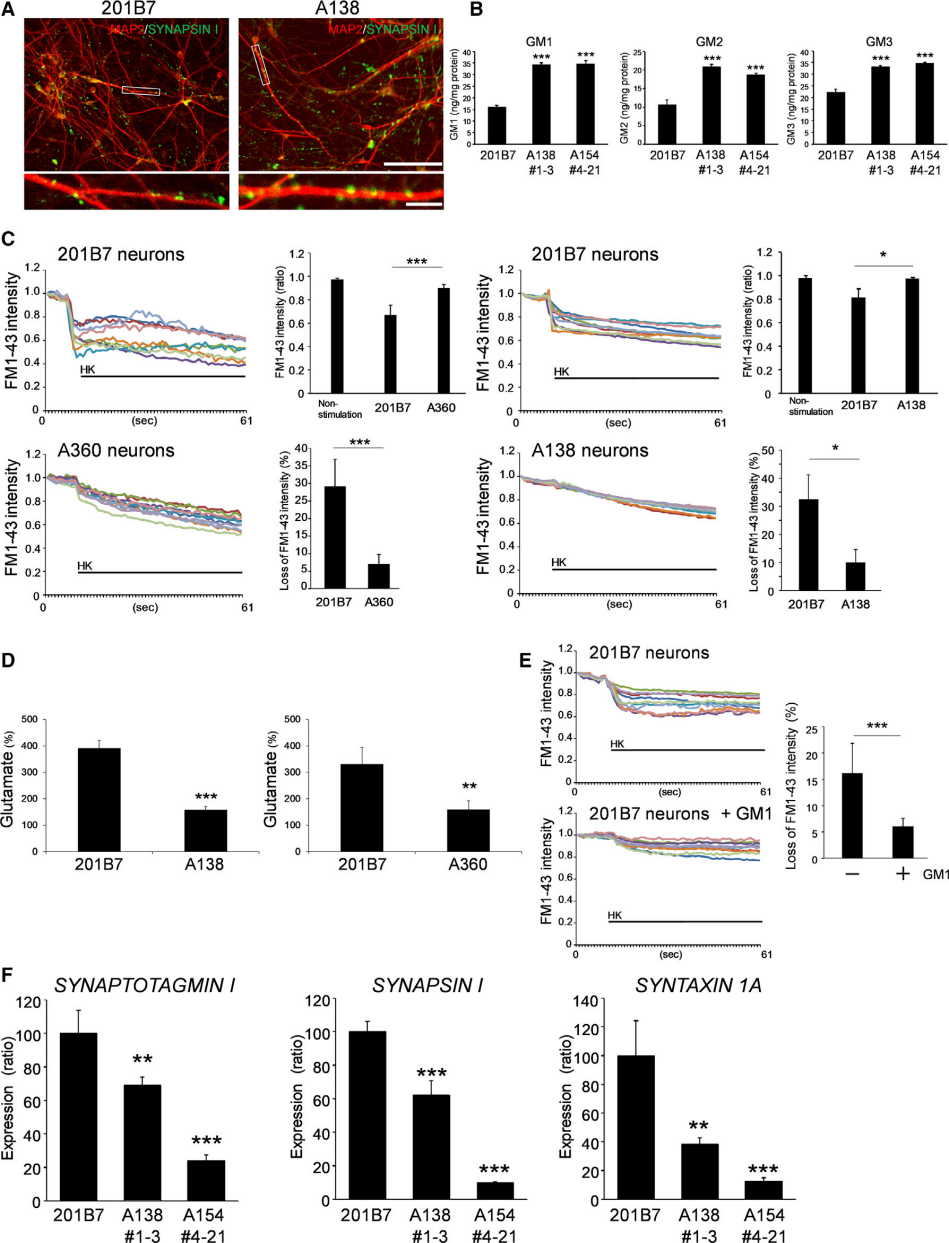
Deficit in Presynaptic Function of GM1 Gangliosidosis Neurons (A) Day 66 differentiated neurons from control (201B7) and disease (A138 1-3) NSCs were immunostained with anti-MAP2 (red) and SYNAPSIN I (green) antibodies. Boxed areas are magnified below each picture. Scale bars: 100 μm (upper), 20 μm (lower). (B) Increased accumulation of GM1, 2, and 3 gangliosides in disease neurons. Quantification by LC-MS analysis. The bars represent the mean ± SD from three independent experiments. ^∗∗∗^p < 0.001, disease versus normal, Student's t test. (C) Decreased exocytotic release in the gangliosidosis-derived neurons. To examine the exocytotic process, the loss of FM1-43 dye fluorescence was monitored. High potassium (HK) solution (50 mM) was applied for cell depolarization. Left: A360 1-3-disease neurons on day 62. Right: A138 1-3-disease neurons on day 98. Each trace indicates a changed intensity of FM1-43 fluorescence from single vesicle-like buttons. The decreased fluorescence intensity of FM dye was determined by comparing the stimulation intensity (2 s after HK+ stimulation) with basal levels (2 s before the stimulation). The quantitative data are shown as the value of the basal/HK+-stimulated ratio (upper), and loss of FM intensity (%): (basal intensity − HK+-stimulated intensity − fading value of fluorescence) × 100 (lower). ^∗^p < 0.05 (n = 10, where n indicates the number of randomly selected buttons). (D) Decreased release of glutamate from GM1 gangliosidosis-derived neurons. Left: A138 1-3-disease neurons on day 69. Right: A360 1-3-disease neurons on day 65. The data are displayed as the ratio (%) of the HK+-stimulated release/basal release. Basal release (1 min) was collected before the stimulation with HK (high KCl, 50 mM, 1 min). ^∗∗^p < 0.01, ^∗∗∗^p < 0.001 (n = 4, where n is the number of wells for each experimental condition). (E) Presynaptic function in control neurons with or without exposure to exogenous GM1 ganglioside. 200 nM GM1 ganglioside was applied throughout the time course of neural differentiation (85 days). Right: the quantitative data. ^∗∗∗^p < 0.001 (n = 10, mean ± SD). (F) Decreased mRNA levels of presynapse proteins in disease neurons. qPCR. ^∗∗^p < 0.01, ^∗∗∗^p < 0.001, disease versus normal, Student's t test (n = 4 independent samples).

We next examined *in vitro* synaptic function in the neuralized NSCs after long-term maintenance with neural differentiation medium. MAP2-positive differentiated neurons derived from both control (201B7) and disease (A138) NSCs showed many SYNAPSIN I-positive synaptic puncta (Figure 2A), and similarities in neuronal composition among the control and disease-derived cultures, including glutamatergic (*vGLUT1*) and GABAergic (*GAD67*) populations, were determined by qPCR analysis (Figure S2A). Similar to the NSCs, differentiated neurons derived from GM1-iPSCs exhibited increased accumulation of GM1, 2, and 3 gangliosides compared with the control neurons (Figure 2B). To explore the possible influence of this increased intracellular GM1 ganglioside on neuronal maturation and/or synaptic function, we next investigated cell survival and neurite outgrowth. Even though the exogenous treatment of GM1 ganglioside was able to increase the amount of GM1 ganglioside in the normal cells (Figure S2B), there was no difference in the number of MAP2-positive surviving cells between control and disease-generated neurons with or without GM1 ganglioside exposure, nor indeed in total neurite length or neurite outgrowth in both control and disease neurons with or without GM1 ganglioside exposure (Figures S2C and S2D). Furthermore, the lactate dehydrogenase (LDH) cytotoxicity assay confirmed that cell death was not enhanced with or without GM1 ganglioside exposure either, comparing with the controls (Figure S2E). These findings support that accumulated GM1 ganglioside plays a negligible role in cell survival and neurite outgrowth during neuronal differentiation. To then examine synaptic function more directly, we monitored an exocytosis process using FM1-43 imaging. In synaptic sites, functional mature neurons release neurotransmitters, which are visualized with fluorescent-labeled FM1-43 dye, and the loss of fluorescent intensity provides an exocytosis index (Numakawa et al., 1999). The fluorescent signal of FM1-43 disappeared in control neurons after application of a high potassium solution (50 mM) to induce cell depolarization, whereas it failed to reduce in the A154 and A360 disease-derived neurons (maintained for 62 days) (left panels in Figures 2C and S2F), suggesting a reduction of exocytotic activity. The disease neurons maintained for longer durations (A138 at day 98) still exhibited diminished exocytotic activity (right panels in Figure 2C).

We next asked whether or not the reduction of exocytotic activity affected neurotransmitter release. Neurotransmitters such as glutamate are mainly released at the synapses with exocytosis. When release is impaired, the amount of the neurotransmitter decreases in the culture containing the neurons after depolarization compared with normal. As expected, the amount of glutamate was lower in the culture containing GM1 gangliosidosis-derived neurons than in that containing control neurons (Figure 2D). Taken together, these results suggested that the reduction of exocytosis impaired neurotransmitter release in GM1 gangliosidosis-derived neurons.

To confirm the results thus far, we took advantage of a GM1 gangliosidosis animal model, the BKO mouse, which carries the null mutation of the *Glb1* gene encoding β-galactosidase and mimics the disease phenotype, including GM1 ganglioside accumulation in neural cells (Matsuda et al., 1997). We isolated cortical neurons from the brain of the model and examined neurotransmitter release using the same method as described here. The primary cultured neurons derived from BKO mice failed to release FM1-43, confirming impaired presynaptic functions such as neurotransmitter release in GM1 gangliosidosis neurons (Figure S2G).

We next investigated whether or not supplying β-GAL enzyme restored the impairment in exocytosis in GM1 gangliosidosis-derived neurons. The exocytosis activity was partially restored with β-GAL treatment in GM1 gangliosidosis-derived neurons, suggesting that β-GAL deficiency affected exocytosis in the neurons (Figure S2H).

Long-term treatment with GM1 ganglioside could significantly reduce the releasing efficiency of FM1-43, even in control neurons (Figure 2E). The treatment was able to increase the amount of GM1 ganglioside in the normal cells (Figure S2B). These results suggest that the accumulated GM1 ganglioside affects neurotransmitter release into the synaptic sites *in vitro*. Consistent with such a presynaptic dysfunction, we observed a significant decrease in mRNA expression of presynapse proteins, including *SYNAPTOTAGMIN I*, *SYNAPSIN I*, and *SYNTAXIN* 1A in both A138- and 154-disease neurons (Figure 2F) (Hicks et al., 1997). Therefore, it is possible that accumulated GM1 during neuronal development results in dysfunctional presynaptic machinery.

### High-Content Screening for GM1 Gangliosidosis Drug Candidates

**Figure 3:**
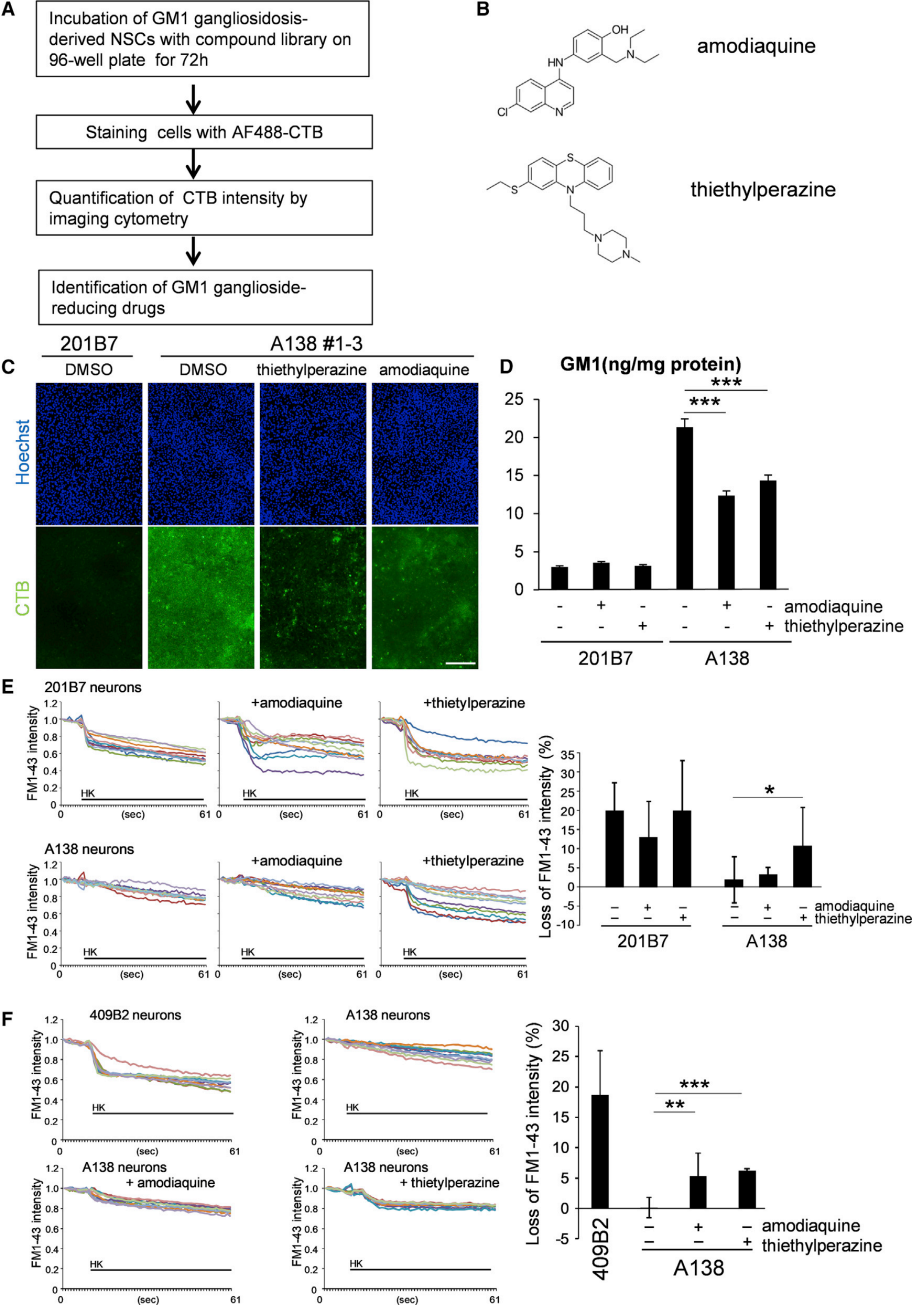
*In Vitro* Presynaptic Function Is Improved with Treatments with the Drug Candidates (A) Flowchart of imaging cytometry-based high-content screening. A138 1-3 NSCs were plated onto 96-well plates and incubated with 5 μM of validated compounds for 72 h. The cells were stained with AF488-CTB, and the fluorescence intensity was measured using an IN Cell Analyzer 6000 (GE Healthcare). (B) Skeletal formula of two hit compounds, amodiaquine and thiethylperazine. (C) Representative images of cells stained with AF488-CTB in the hit-compound-treated wells. Scale bars, 100 μm. (D) Reduction of GM1 ganglioside in control NSCs (201B7) and disease NSCs (A138 1-3) treated with amodiaquine and thiethylperazine. Gangliosides were purified from the NSCs treated with amodiaquine (5 μM) and thiethylperazine (5 μM) and were measured by LC-MS. The bars represent the mean ± SD from three independent experiments. ^∗∗∗^p < 0.001, treated versus non-treated NSCs, Student's t test. (E) Effect on the abnormal exocytotic release after short-term (for 24 h before the FM1-43 imaging) treatments with 1 μM amodiaquine or 1 μM thiethylperazine. Each trace shows the signal of FM1-43 intensity from single FM1-43 buttons (n = 10, where n is the number of randomly selected buttons). Right: the quantitative data for loss of FM intensity (%): (basal intensity − HK+-stimulated intensity − fading value of fluorescence) ×100. ^∗^p < 0.05 (n = 10 where n is the number of randomly selected buttons). (F) Improvement of the abnormal exocytotic release of A138 1-3 neurons after long-term treatments (61 days) with 1 μM amodiaquine or 1 μM thiethylperazine. The neural cells were treated throughout the time course of neural differentiation. Right: the quantitative data were calculated as described in (E). ^∗∗^p < 0.01, ^∗∗∗^p < 0.001 (n = 10 where n is the number of randomly selected buttons).

The effect of GM1 ganglioside accumulation on presynaptic function *in vitro* prompted us to seek candidate compounds to reduce this abnormal ganglioside level in disease cells (Figure 3A). To establish the necessary high-content screening (HCS) for such an aim, we took advantage of iPSC-derived NSCs, because they are more easily expandable than post-mitotic differentiated neurons, and precise detection of cytoplasmic AF488-CTB signals representing an accumulation of GM1 ganglioside was easily achieved (i.e., neurons are too thin to meaningfully visualize the AF488-CTB signal). A138 NSCs seeded in 96-well plates were incubated with 2,217 compounds containing already approved drugs and major chemicals used in pathway analyses (provided by Drug Discovery Initiative, University of Tokyo, and purchased from Sigma-Aldrich) for 72 h, and then stained with AF488-CTB for quantification of cellular fluorescence in each well by image analysis (Figure 3A). Using this drug-screening system in combination with the approved drug library revealed 25 small compounds that reduced the accumulation of GM1 ganglioside. Of these, we focused on the two best compounds, amodiaquine and thiethylperazine, to analyze their effects in detail (Figures 3B, 3C, and S3A and Table 1). LC-MS analysis confirmed the effect of these compounds on ganglioside concentration in disease cells and revealed that GM1, GM2, and GM3 gangliosides were all significantly reduced in the A138 NSCs (Figures 3D and S3B).

**Table 1:** List of Hit Compounds that Can Reduce the GM1 Ganglioside Accumulation

Compound Name	Comments	GM1 Accumulation (%)
Amodiaquine dihydrochloride dihydrate	Antimalarial, heme polymerase inhibitor, schizonticide	16.83736026
Thiethylperazine malate	Antiemetic, adrenergic antagonist	16.85138567
Sulfamerazine	Antibacterial, inhibitor of folic acid synthesis	17.03440890
Ungerine nitrate	Sedative, enhancer of analgesics	17.36768776
Amiodarone hydrochloride	Antiarrhythmic, Na^+^ channel blocker, anti-anginal, K^+^ channel blocker, non-competitive beta-adrenergic blocker	21.31707824
Sertindole	Antipsychotic	23.97221777
Delcorine	Antiarrhythmic, nicotinic ligand, hypotensive	24.28350616
Perphenazine	Antipsychotic, dopamine antagonist, tranquilizer	25.89101795
Althiazide	Diuretic, Na^+^ Cl^−^ transport inhibitor, antihypertensive	31.11239360
Diethylstilbestrol	Estrogen, antineoplastic	31.23824793
Acacetin	Antioxidant, inhibitor of topoisomerase I, antitumor agent, inhibitor of glutathione reductase	31.35048887
Harmol hydrochloride monohydrate	Anxiolytic, liver conjugation probe	34.48043792
Skimmianine	Sedative, 5-HT ligand, anticonvulsant	37.60694168
Succinylsulfathiazole	Antibacterial, inhibitor of folic acid synthesis	37.63117478
Fillalbin	Tropane alkaloid	40.66893369
Canavanine sulfate monohydrate (L,+)	Anticancer agent, nitric oxide inductible synthetase inhibitor, cholesterol-lowering agent	43.11514126
Harmaline hydrochloride dihydrate	Antihelminthic, monoamine oxidase inhibitor, antimalarial, vasorelaxant	45.67007687
Miglustat	Inhibitor of glucosylceramide synthase	52.72368500
Trihexyphenidyl-D,L hydrochloride	Antiparkinsonian, anticholinergic	54.37458264
Fluoxetine hydrochloride	Antidepressant, 5-HT uptake inhibitor	57.79212899
Lovastatin	Anti-hypercholesterolemic, HMG CoA reductase inhibitor	60.79971522
Haloperidol	Antipsychotic, dopamine antagonist, neuroleptic, alpha antagonist, antiemetic	65.56089756
Prenylamine lactate	Vasodilator, calcium channel blocker	68.01129453
Bromperidol	Antipsychotic, dopamine antagonist	68.94978044
Convolamine hydrochloride	Vasodilatator, cholinergic ligand, local anesthetic, hypotensive	70.32706820

Hit compounds found in Figure 3A are listed (selected compounds that reduced GM1 ganglioside by more than 20%). GM1 accumulation (%) is calculated as the percentage of (A_T_ – N_U_)/(A_U_ – N_U_). A_T_, CTB fluorescence intensity of compound-treated A138 NSCs; A_U_, CTB fluorescence intensity of untreated A138 NSCs; N_U_, CTB fluorescence intensity of untreated 201B7 NSCs.

We next examined whether amodiaquine and thiethylperazine could restore the presynaptic deficit in disease-derived neurons. Short-term treatment (for 24 h before FM1-43 imaging) with thiethylperazine, but not amodiaquine, significantly recovered the presynaptic dysfunction in A138 neurons (Figure 3E). In contrast, long-term treatment with amodiaquine or thiethylperazine throughout the time course of *in vitro* neural differentiation restored the decreased presynaptic activity of A138 neurons (Figure 3F), and of the other iPSC clones, A154 and A360 (Figures S3C and S3D).

The treatments also significantly restored the decrease of glutamate in the culture containing the GM1 gangliosidosis-derived neurons (Figures S3E and S3F). These results suggest that both amodiaquine and thiethylperazine can reduce GM1 ganglioside accumulation and recover the presynaptic dysfunction characteristic of disease-derived neurons.

### Amodiaquine and Thiethylperazine Upregulate Lysosomal Glycosphingolipid Degradation Enzymes and Activate Autophagy

**Figure 4:**
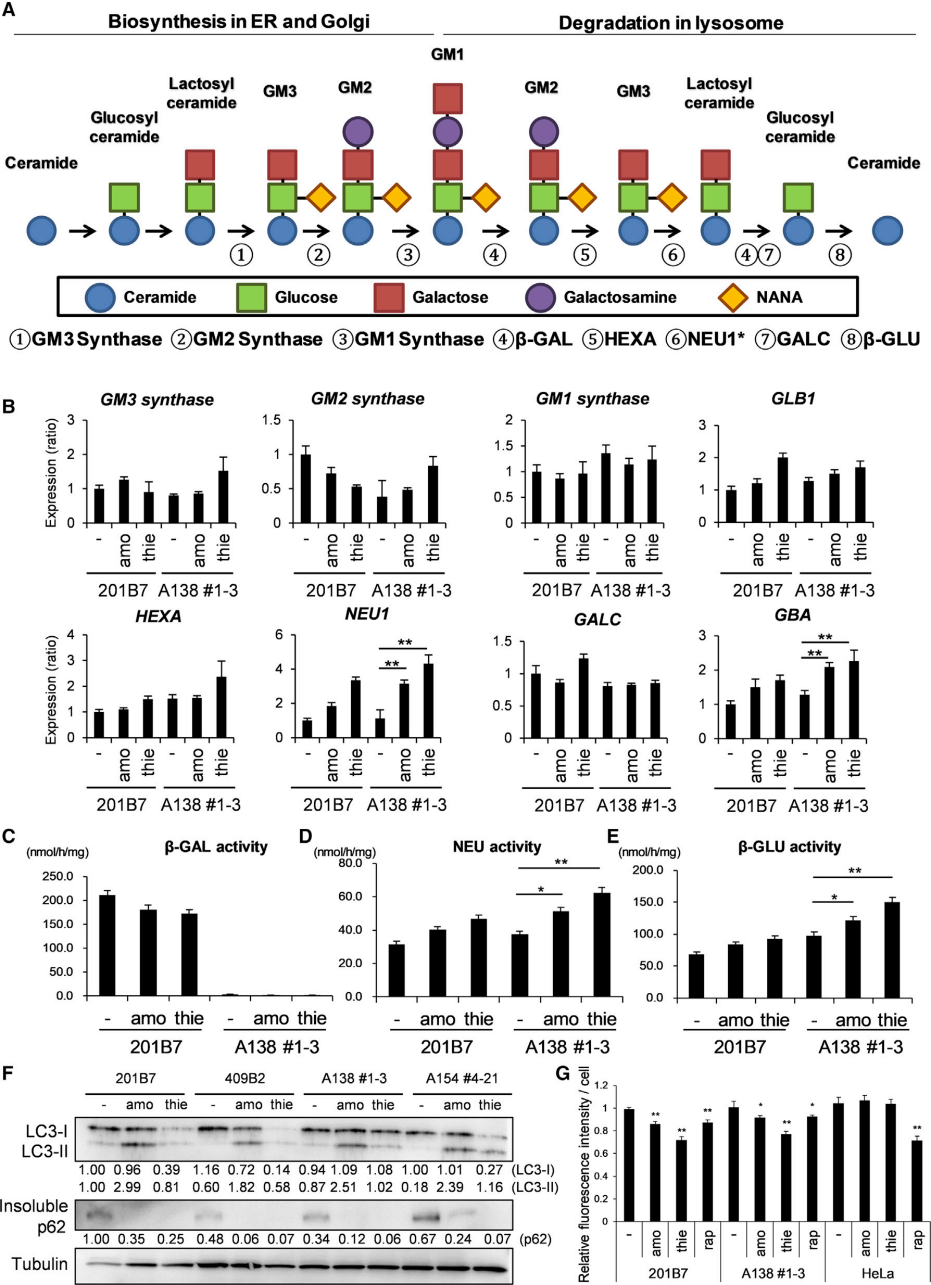
Amodiaquine and Thiethylperazine Enhance Lysosomal Glycosphingolipid Degradation and Activate Autophagy (A) Schemes for the synthetic and catabolic pathways of glycosphingolipid. GM1, GM2, and GM3 gangliosides are synthesized from ceramide by the sequential addition of monosaccharide or N-acetylneuraminic acid (NANA). Ganglioside catabolism occurs in reverse order from their anabolism, with the stepwise actions of lysosomal glycosidase specific for gangliosides. Number 1–8 indicate the enzymes of each reaction. ^∗^It is controversial that Neu1 is involved in the degradation of GM3 ganglioside (Yoshino et al., 1990, O'Leary and Igdoura, 2012). (B) Expression of *NEU1* and *β-GLU* is elevated with the candidate treatments. Normal (201B7) and disease-derived (A138 1-3) NSCs were treated with 5 μM amodiaquine and 5 μM thiethylperazine for 72 h qPCR. Data were normalized to the levels in 201B7-NSCs (−) (mean ± SD from three independent experiments). ^∗∗^p < 0.01, treated versus non-treated NSCs, Student's t test. amo, amodiaquine; thie, thiethylperazine. (C–E) Enzyme activities of NEU1 and β-GLU are also enhanced with the candidate treatments. NSCs were treated with 5 μM amodiaquine (amo) and 5 μM thiethylperazine (thie) for 48 h and enzyme activities of β-GAL (C), NEU1 (D), and β-GLU (E) were measured. Enzyme activities are expressed as nmol/h/mg protein (mean ± SD from three independent experiments). ^∗^p < 0.05, ^∗∗^p < 0.01, treated versus non-treated NSCs, Student's t test. (F) Autophagic flux was upregulated in NSCs treated with amodiaquine and thiethylperazine. The protein levels of LC3-II were increased and those of insoluble p62 decreased in NSCs treated with both compounds. NSCs were treated with 5 μM amodiaquine (amo) and 5 μM thiethylperazine (thie) for 72 h, western blotting. The band intensities were measured using ImageJ software, normalized to the non-treated 201B7 and described below the images. (G) Measurement of autophagic flux using a GFP-LC3-RFP-LC3ΔG probe. NSCs stably expressing GFP-LC3-RFP-LC3ΔG were treated with the candidates and 1 μM rapamycin (rap) as described in (F). The fluorescence intensity was measured using an IN Cell Analyzer 6000 and the fluorescence intensity ratio (GFP/RFP) of the samples was normalized to that of non-treated 201B7. ^∗^p < 0.05, ^∗∗^p < 0.01 (n = 5 independent samples).

GM1 ganglioside is biosynthesized via a series of synthase processes that start with ceramide in the endoplasmic reticulum and Golgi apparatus and is subsequently degraded by lysosomal hydrolases (Figure 4A) (Venier and Igdoura, 2012). To clarify possible mechanisms underlying the downregulation of GM1 ganglioside by our two hit compounds, we investigated whether the metabolism of GM1 ganglioside is altered after treatment with amodiaquine or thiethylperazine. After treating disease-derived NSCs with each compound for 72 h, we measured the mRNA levels of a series of enzymes on GM1 ganglioside synthesis and metabolic pathways by qRT-PCR. There was no significant change in expression levels of the biosynthetic synthases (for GM1, GM2, and GM3), *HEXA, GALC*, and *GLB1* (Figure 4B). In contrast, the expression of *NEU1* and *GBA* (which encodes β-GLU) was significantly upregulated in the treated patient-derived NSCs compared with those in vehicle-treated NSCs (Figure 4B). In addition, expression levels of other members of NEU family, *NEU2, NEU3*, and *NEU4*, were not increased in the treated NSCs compared with the non-treated NSCs (Figure S4A). Accordingly, the enzyme activities of NEU1 and β-GLU were also enhanced with amodiaquine and thiethylperazine treatments, while β-GAL activity was unaffected (Figures 4C–4E and S4B). Combined with our finding of reduced GM1, GM2, and GM3 ganglioside accumulation with the two hit compounds (Figures 3D and S3B), the enzyme analyses suggest activation of the lysosomal sphingolipid degradation pathway, and consequently, downregulation of ganglioside accumulations.

Cellular autophagy is impaired in lysosomal storage diseases (Lieberman et al., 2012, Maetzel et al., 2014, Soga et al., 2015), thus here, we investigated the autophagy pathway in NSCs treated with the hit compounds, focusing on autophagy markers such as microtubule-associated protein 1 light chain 3-II (LC3-II) and the insoluble p62/SQSTM1 (p62). The C-terminal processing of LC3 produces LC3-I, which is modified to LC3-II following the initiation of autophagosome formation (Kabeya et al., 2000). Because p62/SQSTM1 (p62) binds to LC3 and is degraded upon fusion with the lysosome, impairment of autophagy flux accumulates insoluble p62 (Komatsu et al., 2007). Thus, we confirmed the upregulation of LC3-II and insoluble p62 in GM1 gangliosidosis-derived skin fibroblasts compared with normal controls and suggested that although autophagy initiation is upregulated in fibroblasts, the autophagic flux is impaired (Figure S4C). Surprisingly, both markers were not enhanced in GM1 gangliosidosis-derived NSCs compared with normal cells (Figures 4F and S4D); however, treatment with amodiaquine and thiethylperazine enhanced the expression of LC3-II and reduced insoluble p62 in both normal and GM1 NSCs (Figures 4F and S4D). Consistently, p62 granules were detected in both NSCs treated with the two compounds, appearing only in those cells with high autophagy activity (Figure S4E) (Ben Younes et al., 2011). To further assess autophagy activity, we took advantage of GFP-LC3-RFP-LC3ΔG, a fluorescent probe for calculating autophagy flux. This probe is cleaved by endogenous ATG4 into equal amounts of GFP-LC3 and RFP-LC3ΔG. While GFP-LC3 is degraded by autophagy, RFP-LC3ΔG remains in the cytosol and acts as an internal control. Thus, autophagic flux can be estimated by calculating the GFP/RFP signal ratio, which is decreased with the activation of autophagy. Treatments with amodiaquine and thiethylperazine significantly reduced the GFP/RFP signal ratio in both normal and patient NSCs (Figures 4G and S4F). The reduction levels were similar to that of the treatment with rapamycin, which is known as an activating drug for autophagy (Figure 4G) (Kaizuka et al., 2016). In contrast, the treatments with amodiaquine and thiethylperazine did not reduce the ratio in HeLa carcinoma cells (Figures 4G and S4F). The results suggest that the two compounds have potential to activate autophagy, and their effects on autophagy were dependent on the cell type.

Taken together, these results indicate that the two hit compounds can reduce GM1 ganglioside accumulation and upregulate both autophagy and enzyme activities involved in lysosomal sphingolipid degradation.

### Hit Compounds Suppress GM1 Ganglioside Accumulation *In Vivo*

**Figure 5:**
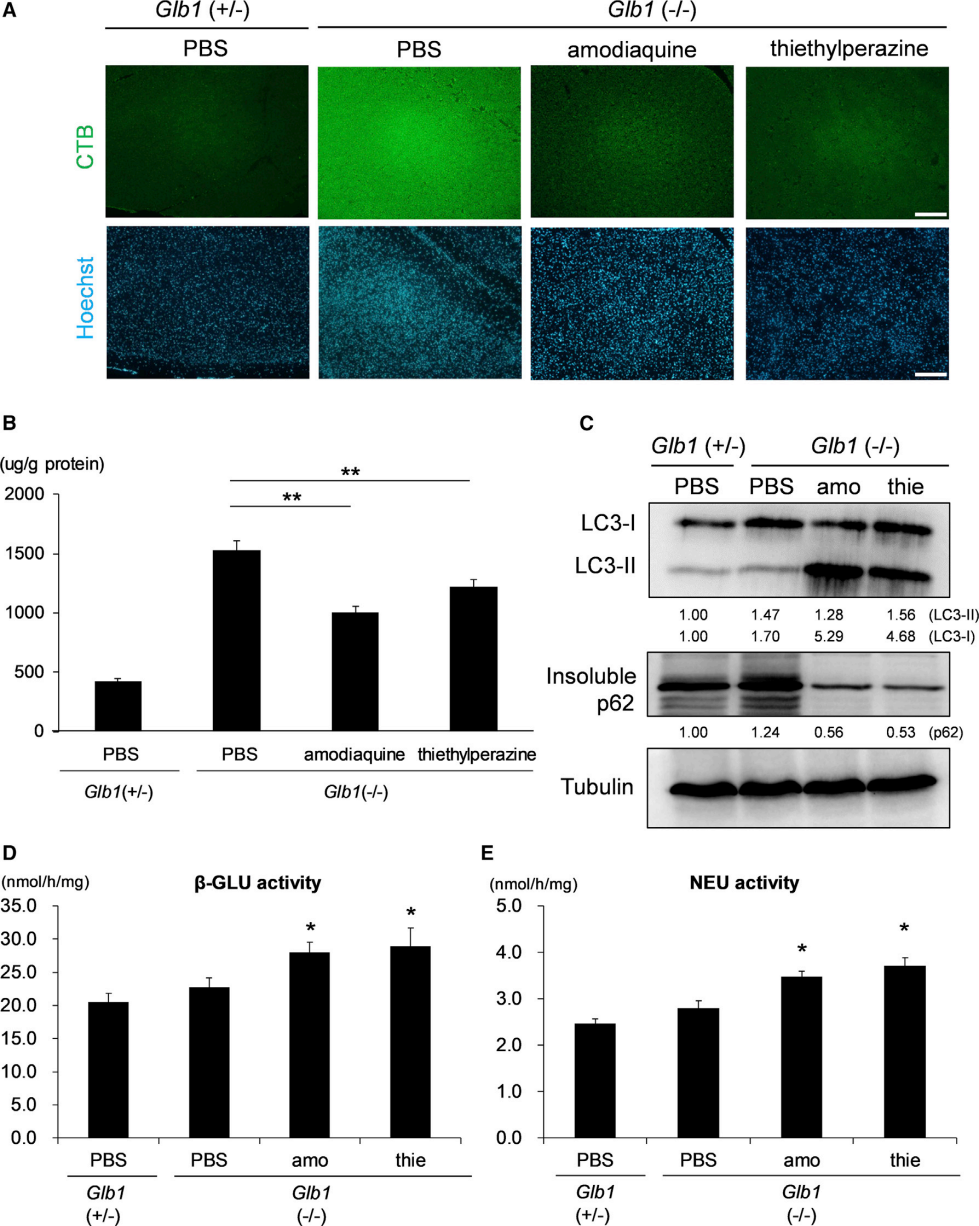
Treatments with Amodiaquine and Thiethylperazine Can Reduce GM1 Ganglioside Accumulations in the Brain of Model Mice (A) β-GAL KO (BKO, *Glb1* −/−) mice were treated with amodiaquine (40 mg/kg, i.p.) and thiethylperazine (6 mg/kg, i.p.) twice a day from P9 to P15. Brain slices were stained with AF488-CTB and Hoechst to visualize GM1 ganglioside accumulation. Scale bars, 200 μm. (B) The treatments with two candidates reduced GM1 ganglioside accumulation in β-GAL KO mice, which were treated as described in (A). GM1 ganglioside was extracted from brain homogenates and measured by LC-MS. (C) The treatments with our two candidates activated autophagy in β-GAL KO mice, which were treated as described in (A). The brain homogenates were prepared and subjected to western blot analysis with anti-LC3B, anti-tubulin, or anti-p62 antibodies. The band intensities were measured using ImageJ software, normalized to the PBS-treated *Glb1* (+/−) and described below the images. (D and E) Enzyme activities of NEU and β-GLU were also enhanced with the candidate treatments in β-GAL KO mice, which were treated as described in (A). The brain homogenates were prepared and the enzyme activities of β-GLU (D), and NEU (E) were measured. ^∗^p < 0.05, each candidate-treated *Glb1* (−/−) versus PBS-treated *Glb1* (−/−), Student's t test (n = 3 independent samples).

Finally, we examined whether the hit compounds identified were effective *in vivo*. To verify the preventive effect of hit compounds against an accumulation of GM1 ganglioside in the brain, we treated BKO mice with amodiaquine (40 mg/kg, i.p.) or thiethylperazine (6 mg/kg, i.p.) twice a day from postnatal day 9 (P9) to 15. Then, mice brains were sampled and analyzed to quantify levels of GM1 ganglioside; AF488-CTB staining revealed that both amodiaquine and thiethylperazine treatments could reduce the signal of GM1 ganglioside in the BKO mice compared with the controls (Figures 5A and S5A). Quantification of GM1 ganglioside content by LC-MS showed a significant reduction of GM1 ganglioside accumulation in the brain of treated mice (Figure 5B). Similar to the results *in vitro*, the treatments also activated autophagy and enhanced β-GLU and NEU1 but not β-GAL activities (Figures 5C–5E and S5B). These results indicate that treatment with amodiaquine and thiethylperazine affects GM1 accumulation in the brain of model mice.

## Discussion

Here, we generated iPSCs from the cells of GM1 gangliosidosis patients and demonstrated that these lines provide a cellular model of pathological neural dysfunction. Using iPSCs, we found drug candidates for future therapy that reduced GM1 ganglioside accumulation *in vitro* and *in vivo*. Specifically, the candidate compounds activated both the metabolic pathway from GM3 ganglioside to ceramide and the autophagic response.

Considering that neural cells are predominantly affected in GM1 gangliosidosis (Sandhoff and Harzer, 2013), we directed the iPSCs to differentiate into both NSCs and mature neurons. Using this system, we confirmed excessive GM1 ganglioside accumulation in both cell types and low β-GAL activity compared with normal controls. While the pathogenesis of GM1 gangliosidosis most likely involves a massive and progressive increase of GM1 ganglioside in neural cells, a more precise understanding of the downstream effects of such cellular events has yet to be elucidated. Some studies implicated the accumulation of GM1 ganglioside in causing neuronal apoptosis via the UPR and endoplasmic reticulum calcium signaling in a mouse model (Sano et al., 2009, Tessitore et al., 2004); however, we did not detect cell death in the neuronal differentiation of GM1 gangliosidosis iPSCs. These discrepancies could be in part due to differences in the species studied (mouse versus human), cell types (MEF, neurosphere versus neuron), and β-GAL gene mutations (null versus point mutation). On the other hand, our analysis revealed impaired neurotransmitter release from presynaptic sites in mature neurons derived from GM1 gangliosidosis iPSCs and the mouse model. The GM1 gangliosidosis-derived mature neurons exhibited a similar number of SYNAPSIN I-positive puncta to control neurons. The neurons expressed less of the essential synapse-associated molecules than controls, including *SYNAPTOTAMINE I*, *SYNAPSIN I*, and *SYNTAXIN* 1A (Hicks et al., 1997). It has been well accepted that synaptic vesicles containing snare proteins, such as SYNTAXIN 1A, and other presynaptic proteins, including SYNAPTOTAMINE I and SYNAPSIN I, have a pivotal role in exocytotic release of neurotransmitters in neurons (Hilfiker et al., 1999, Li and Chin, 2003, Tucker and Chapman, 2002, Vardar et al., 2016). Therefore, our results suggest that the disease-associated defect in neurotransmitter release is due to their reduced expression at individual presynaptic sites. We also found that long-term treatment with GM1 ganglioside during neural differentiation affected neurotransmitter release from the presynapse even in normal iPSC-derived neurons. This study thus supports a pathogenic mechanism underlying neural dysfunction in GM1 gangliosidosis, in which the excess accumulation of GM1 ganglioside inhibits synaptic function via the impairment of synapse-associated molecules during neuronal differentiation.

Considering that GM1 ganglioside itself could cause synaptic dysfunction, removing the accumulated GM1 ganglioside should be effective in treating the disease. To this end, we sought to identify drug candidates that could suppress the accumulation of GM1 ganglioside using HCS with iPSC-derived NSCs. Using a chemical library containing 2,217 compounds, we found 25 compounds that reduced GM1 ganglioside accumulation in NSCs. Among them, amodiaquine and thiethylperazine also reduced GM1 ganglioside accumulation in the model mice. Accumulated GM1 ganglioside was observed in the brain of BKO mice as early as when they were born. With regard to the clinical symptoms, they were born normal and healthy at least until 4 months of age. Therefore, we investigated the effects of two compounds on the accumulation in 15-day aged BKO mice. The previous study also reported that the mice developed definite neurological symptoms from 6 months of age and they died at 7–10 months of age (Matsuda et al., 1997). Although this study did not approach the neurological symptoms and the survival rate in the compounds-treated mice, further study is required to find out whether the compounds are still effective on the neurological symptoms and the survival rate *in vivo*. Amodiaquine and thiethylperazine also restored the defective neurotransmitter release at the synapse of patient-derived neurons. While it remains unclear whether reducing GM1 ganglioside can recover neuronal function in clinical GM1 gangliosidosis, our results provide the demonstration that suppressing the accumulation of GM1 ganglioside might alleviate the neuronal dysfunction associated with this disease.

Based on this study, we propose two mechanisms underlying the effect of amodiaquine and thiethylperazine on GM1 ganglioside accumulation. First, our results suggest a strategy for GM1 gangliosidosis therapy whereby activation of the metabolic pathway from GM3 ganglioside to ceramide might affect GM1 ganglioside accumulation. Although the treatment with amodiaquine and thiethylperazine reduced GM1 ganglioside accumulation in the patient cells, we did not detect any upregulation of β-GAL activity. In addition, the treatment did not affect the expression of GM gangliosides synthases; however, we did demonstrate enhanced enzymatic activities, including NEU1 and β-GLU in GM1 ganglioside degradation. NEU1 encodes the lysosomal neuraminidase enzyme, sialidase, which removes the terminal sialic acid from oligosaccharide chains and metabolizes GM3 ganglioside to lactosylceramide by removing neuraminic acid (Venier and Igdoura, 2012). In the lysosome, this enzyme is also part of a heterotrimeric complex together with β-GAL and cathepsin A (Bonten et al., 2014), and upregulation of NEU1 expression with the treatment might affect formation of the heterotrimeric complex and somehow stabilize the β-GAL component. β-GLU is an enzyme with glucosylceramidase activity that converts glucosylceramide to ceramide in lysosomes (Venier and Igdoura, 2012). Mutations in the β-GLU gene cause Gaucher's disease, which is characterized by neuronal disturbance and could also be associated with Parkinson's disease (Suzuki et al., 2015). With respect to GM1 ganglioside accumulation, we showed that GM2 and GM3 gangliosides accumulated in GM1 gangliosidosis-derived NSCs and neurons, suggesting that synthetic and/or degradative pathways for GM1 ganglioside are stagnated in GM1 gangliosidosis. A previous study showed accumulations of GM2 and GM3 gangliosides in patients’ brain (Hahn et al., 1997). Reactivating the pathways from GM3 ganglioside to ceramide could remove this stasis and progress to relaxation of GM1 ganglioside accumulation. GM2 and GM3 gangliosides are converted from GM1 ganglioside (i.e., GM2 and GM3 gangliosides are downstream products of GM1 ganglioside). At the same time, GM2 and GM3 gangliosides are also substrates for GM1 ganglioside (i.e., GM2 and GM3 gangliosides are also located upstream in the biosynthesis of GM1 ganglioside). Activating the metabolic pathway of GM2 and GM3 ganglioside might affect the conversion into GM1 from precursor gangliosides (GM2 and GM3) in the biosynthesis pathway of GM1 ganglioside.

We also identified autophagy activation as a possible second mechanism by which our drug agents exert their demonstrated effects on neural cells, and cellular autophagy is impaired in lysosomal storage diseases (Lieberman et al., 2012, Maetzel et al., 2014, Soga et al., 2015). We confirmed the upregulations of LC3-II and insoluble p62 in GM1 gangliosidosis-derived skin fibroblasts compared with normal controls, suggesting that autophagy initiation is upregulated in the fibroblasts, in turn impairing autophagic flux; however, both markers were not at all enhanced in GM1 gangliosidosis-derived NSCs. This discrepancy might be due to the rapid turnover of autophagic flux in neural cells compared with non-neural cells (Yue et al., 2009). We found that the tested compounds could enhance the protein levels of LC3-II and downregulate the insoluble p62 expression both in NSCs and in mouse models. Consistently, the treatments also upregulated p62 granules in GM1 gangliosidosis-derived NSCs, and these granules appear in cells with high autophagy activity such as during starvation (Ben Younes et al., 2011). A previous study using iPSCs derived from Niemann-Pick type C disease, a lysosomal storage disorder characterized by excess deposition of cellular cholesterol, showed that autophagy-inducing compounds could have cytoprotective effects even without affecting the deposition of cholesterol itself and that inducing autophagy might be a promising treatment option for lysosomal storage diseases (Maetzel et al., 2014). The present study suggests that such a scenario might also be applied for GM1 gangliosidosis, although our case did not show any cytoprotective effect on the neurons derived from GM1 iPSCs, although activating autophagy could still have a potential effect on GM1 gangliosidosis. We showed that amodiaquine and thiethylperazine reduced GM1 ganglioside accumulation with activating autophagy. Because these compounds activated autophagy in both patient cells and normal cells, they must affect the autophagic pathway directly, i.e., their autophagy-inducing effects were not due to the outcome of reducing GM1 ganglioside accumulation. Autophagy plays a role in the digestion of waste products in cells. Its activation promotes the metabolism of intracellular components. Therefore, the metabolism of GM gangliosides contained in major membrane components of cells may also be activated with activation of autophagy.

In contrast to our results, a previous study demonstrated that amodiaquine treatment inhibited autophagy function in a melanoma cell line (Qiao et al., 2013). In this study, the treatment did not activate autophagy flux in HeLa carcinoma cells. This suggested that the effects on autophagy with amodiaquine and thiethylperazine treatments were dependent on the cell type.

This study highlights the downstream pathway of GM1 ganglioside degradation and autophagy as a potential therapeutic target and presents drug candidates, including amodiaquine and thiethylperazine, for treating GM1 ganglioside accumulation in patients with GM1 gangliosidosis.

## Experimental Procedures

### Imaging Cytometry-Based High-Content Screening for GM1-Reducing Drugs

A138 and 201B7 NSCs were plated at 5 × 10^4^ cells/well in 96-well plates coated with Geltrex, and then compound libraries (1,457 compounds were kindly provided from Drug Discovery Initiative, The University of Tokyo, https://www.ddi.u-tokyo.ac.jp/en/, and 760 compounds were purchased as “Petit screening 2014” from Sigma-Aldrich) were added to each well at 5 μM. After incubation for 72 h, the cells were fixed for 10 min in 4% paraformaldehyde in PBS (pH 7.4) at room temperature, permeabilized with 0.1% Triton X-100 in PBS, and then blocked with 1% BSA in PBS for 10 min at room temperature. GM1 gangliosides in the cells were stained with Alexa Fluor 488-conjugated cholera toxin B (CTB) in PBS, and the fluorescence intensities of CTB staining were measured with an IN Cell Analyzer 6000 using the Developer Toolbox software (GE Healthcare). The CTB fluorescence intensity of untreated 201B7 NSCs was used for negative controls. All the procedures from plating to staining were automated using the Biomek NX laboratory automation workstation (Beckman Coulter). Statistical evaluation of the results yielded a reproducible Z′-factor value range of 0.78–0.81, demonstrating the excellent assay. Z′ factor = 1 − (3 × SD_2_ + 3 × SD_1_)/(X_2_ − X_1_) where SD_1_, X_1_ and SD_2_, X_2_ represent the standard deviations and means of the minimal (1) and maximal (2) signals, respectively (Mayr and Bojanic, 2009).

### Statistics

Unless indicated in the figure legends, an unpaired, 2-tailed Student's t test was used to calculate p values and evaluate the statistical significance of the difference between the indicated samples. The differences were considered to be significant at a 5% level.

### Study Approval

Human samples were collected and analyzed in accordance with approval from the Ethics Committee for Gene Analysis and Genome Research of Kumamoto University. Subjects gave informed consent. All animal experiments conformed to the animal use guidelines of the Committee for Ethics on Animal Experiments of Kumamoto University.

### Other Materials and Methods

Other materials and methods are described in Supplemental Experimental Procedures.

## Author Contributions

R.K., T.N., and T.E. designed the experiments. R.K., T.N., H.O., Y.Y., T.O., and S.I. performed the experiments and analyzed the data. N.F. and H.F. provided the Sendai viruses and patient samples, respectively. R.K., T.N., and T.E. wrote the manuscript. All authors edited and approved the manuscript.

## Supplemental Information

Document S1. Supplemental Experimental Procedures, Figures S1–S6, and Tables S1 and S2

Document S2. Article plus Supplemental Information
