# Supplementary material for: Novel Drug Candidates Improve Ganglioside Accumulation and Neural Dysfunction in GM1 Gangliosidosis Models with Autophagy Activation
Source: Stem Cell Reports. 2020 Apr 16;14(5):909–23. doi: 10.1016/j.stemcr.2020.03.012 (PMC7220856; doi:10.1016/j.stemcr.2020.03.012)
Supplement: Document S1. Supplemental Experimental Procedures, Figures S1–S6, and Tables S1 and S2 [file mmc1.pdf]

Stem Cell Reports, Volume *14*

Supplemental Information

Novel Drug Candidates Improve Ganglioside Accumulation and Neural Dysfunction in GM1 Gangliosidosis Models with Autophagy Activation

Ryutaro Kajihara, Tadahiro Numakawa, Haruki Odaka, Yuji Yaginuma, Noemi Fusaki, Toshika Okumiya, Hirokazu Furuya, Seiji Inui, and Takumi Era

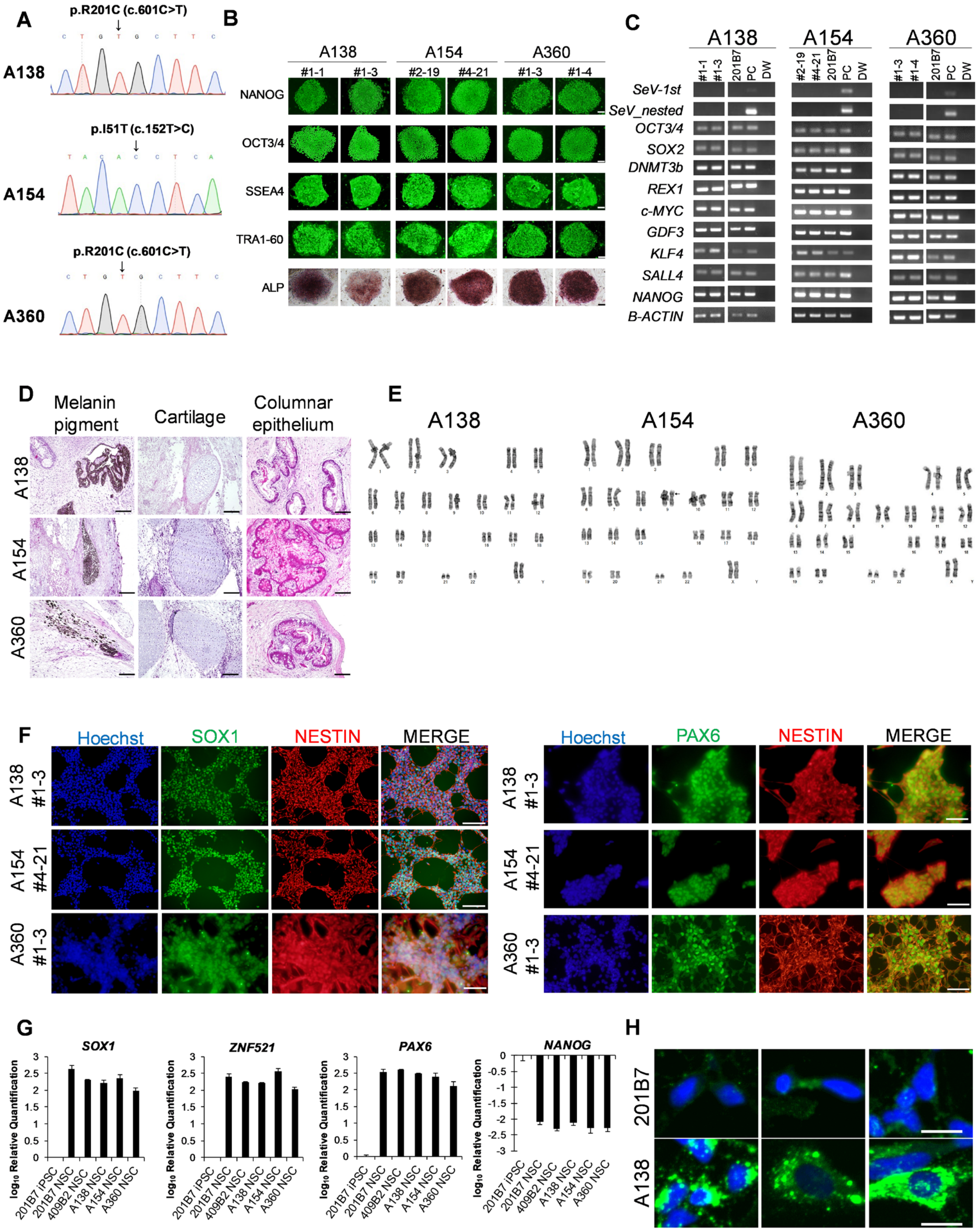

Figure S1. iPSCs and NSCs derived from patients with GM1 gangliosidosis (Related to Figure 1)

(A) Mutations of *GLB1* gene in the patients-derived fibroblasts were confirmed by direct sequencing. The *GLB1* gene was amplified by the genomic PCR and the resultant

PCR products were sequenced using ABI PRISMTM 310 Genetic Analyzer. (B) Immunofluorescence and Alkaline Phosphatase (ALP) staining of iPSC lines for pluripotency markers. The iPSC lines A138 #1-1, 1-3, A154 #2-19, #4-21, A360 #1-3 and #1-4 were generated from skin fibroblasts derived from GM1 gangliosidosis patients. Scale bars, 200 μm. (C) RT-PCR analyses of Sendai virus RNA and pluripotency markers. RT-PCR analyses of GM1 gangliosidosis-derived iPSCs for pluripotency markers. Primers used for *OCT3/4*, *SOX2*, *KLF4,* and *cMYC* were designed to detect the expressions of endogenous genes, but not of transgenes. To detect SeV RNA, nested RT-PCR was performed. PC: positive control, DW: Deionized water as a negative control. (D) Histological analyses of the iPSC-derived teratomas. The iPSC lines, A138 #1-3, A154 #4-21 and A360 #1-3, were transplanted into the testes of NOJ（NOD/Scid/Jak3 KO） immunodeficient mice. Palpable tumors were observed about 12-16 weeks after the transplantation. Tumor samples were collected, fixed in 10% formalin, and processed for paraffin-embedding and hematoxylin-eosin staining following standard procedures. Scale bars, 100 μm. (E) Karyotype analyses of the patients-derived iPSCs, A138 #1-3, A154 #4-21 and A360 #1-3. G band analyses of chromosome were performed by Nihon Gene Research Laboratories. Inc. (Sendai, Japan), according to the manufacturer's protocol. (F) Immunofluorescence staining of the iPSC-derived NSCs for NSC markers. The A138 #1-3, A154 #4-21 and A360 #1-3 iPSC lines were induced for NSC differentiation. Scale bars, 100 μm. (G) Expression of NSC markers *SOX1*, *ZNF521*, *PAX6,* and *NANOG* in the iPSC-derived NSCs, measured by RT-qPCR. The bars represent the mean ± SD from three independent experiments and the ratio value is normalized to the levels in 201B7-iPSCs (Control iPSC). (H) Representative images with the high magnification corresponding to Figure 1B. Green: CTB, Blue: Hoechst. Scale bars, 200 μm.

kajihara *et al*   Figure S2

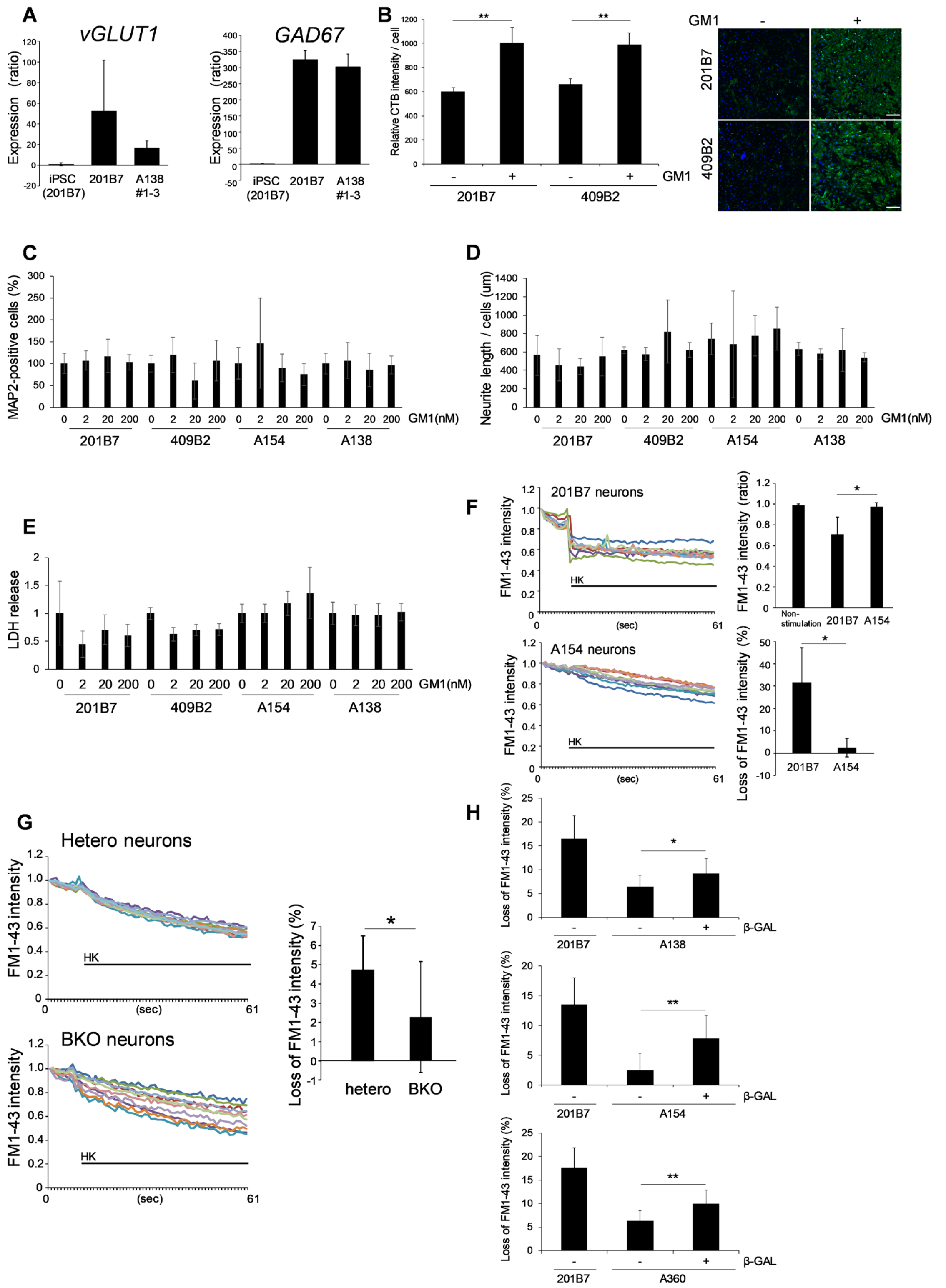

Figure S2. Deficit in presynaptic function of GM1 gangliosidosis neurons (Related to Figure 2)

No alteration in the number of MAP2-positive surviving cells or total neurite outgrowth in control- and disease-neurons with or without exposure to exogenous GM1 ganglioside. (A) Expression of glutamatergic (*vGLUT1*) and GABAergic (*GAD67*) neural markers was determined by qPCR (n = 3-5, n indicates the number of culture dishes in each condition). Data were ratio value normalized to the levels in 201B7-iPSCs. (B) Treatment of GM1 ganglioside in normal NSCs causes accumulation of GM1 ganglioside. Normal NSCs derived from 201B7 and 409B2 iPSCs were treated with 200 nM GM1 ganglioside for 72 hours. The NSCs were stained with AF488-CTB and the fluorescence intensities were measured using IN CELL ANALYZER 6000 (Left). ***P* < 0.01 (n = 4, independent samples) Representative images (Right). Scale bars, 200 µm. (C-E) Immunostaining of MAP2 and LDH leakage assay using neurons at day 66 after the 20-day treatment with GM1 gangliosides (0, 2, 20, or 200 nM). (mean ± SD from three independent experiments). (C); MAP2-positive cells in control (201B7 and 409B2)- and disease (A138 #1-3 and A154 #4-21)-neurons with or without GM1 ganglioside application. Neuronal survival was shown as the relative value of MAP2-positive cells at each GM1 concentration using the non-treatment group for each cell line. (D); quantification data of neurite outgrowth in neurons with or without GM1 ganglioside exposure was also shown. (E); Relative enzymatic activity of lactate dehydrogenase (LDH) in the culture medium was assessed. (F) Decreased exocytotic release in the gangliosidosis-derived neurons. To examine exocytotic process, the loss of FM1-43 dye fluorescence was monitored. High potassium (HK^+) solution (50 mM) was applied for cell depolarization. A154 #4-21-disease neurons on Day 62. Each trace indicates a changed intensity of FM 1-43 fluorescence from single vesicle-like buttons. The decreased fluorescence intensity of FM dye was determined by comparing the stimulation intensity (2 sec after HK+ stimulation) with basal levels (2 sec before the stimulation). The quantitative data were shown as; (Upper) ratio value of basal/ HK+-stimulated one, and (Lower) loss of FM intensity [%: (basal intensity − HK+-stimulated one − fading value of fluorescence) × 100], respectively. *P < 0.05 (n = 10, n indicates the number of randomly selected buttons). (G) Decreased exocytotic release in the BKO mice-derived neurons. To examine exocytotic process during the depolarization at the presynaptic sites, loss of FM1-43 dye fluorescence was monitored. HK^+ solution (50 mM) was applied for cell depolarization. Each trace indicates the changed intensity of FM 1-43 fluorescence from single vesicle-like buttons. The decreased fluorescence intensity of FM dye was determined by comparing the stimulation intensity (2 sec after HK+ stimulation) with basal levels (2 sec before the stimulation). The quantitative data are shown as loss of FM intensity [%: (basal

intensity − HK+-stimulated one − fading value of fluorescence) ×100]. * $P < 0.05$ ($n = 10$, n indicates the number of randomly selected buttons). Hetero neurons, neurons derived from β-GAL heterogeneous (*Glb1* +/-) mice; BKO neurons, neurons derived from β-GAL KO (*Glb1* -/-) mice. (H) Effects of β-GAL treatment on the neural cells. Improvement of the abnormal exocytotic release in the disease-derived neurons after the long-term treatments with 200 nM β-GAL. The neural cells were treated throughout the entire time course of neural differentiation. Left: A138 #1-3 on day 60. Middle: A154 #4-21 on day 87. Right: A360 #1-3 on day 62. *$P < 0.05$, **$P < 0.01$ ($n = 10$, mean ± SD).

kajihara *et al* **Figure S3**

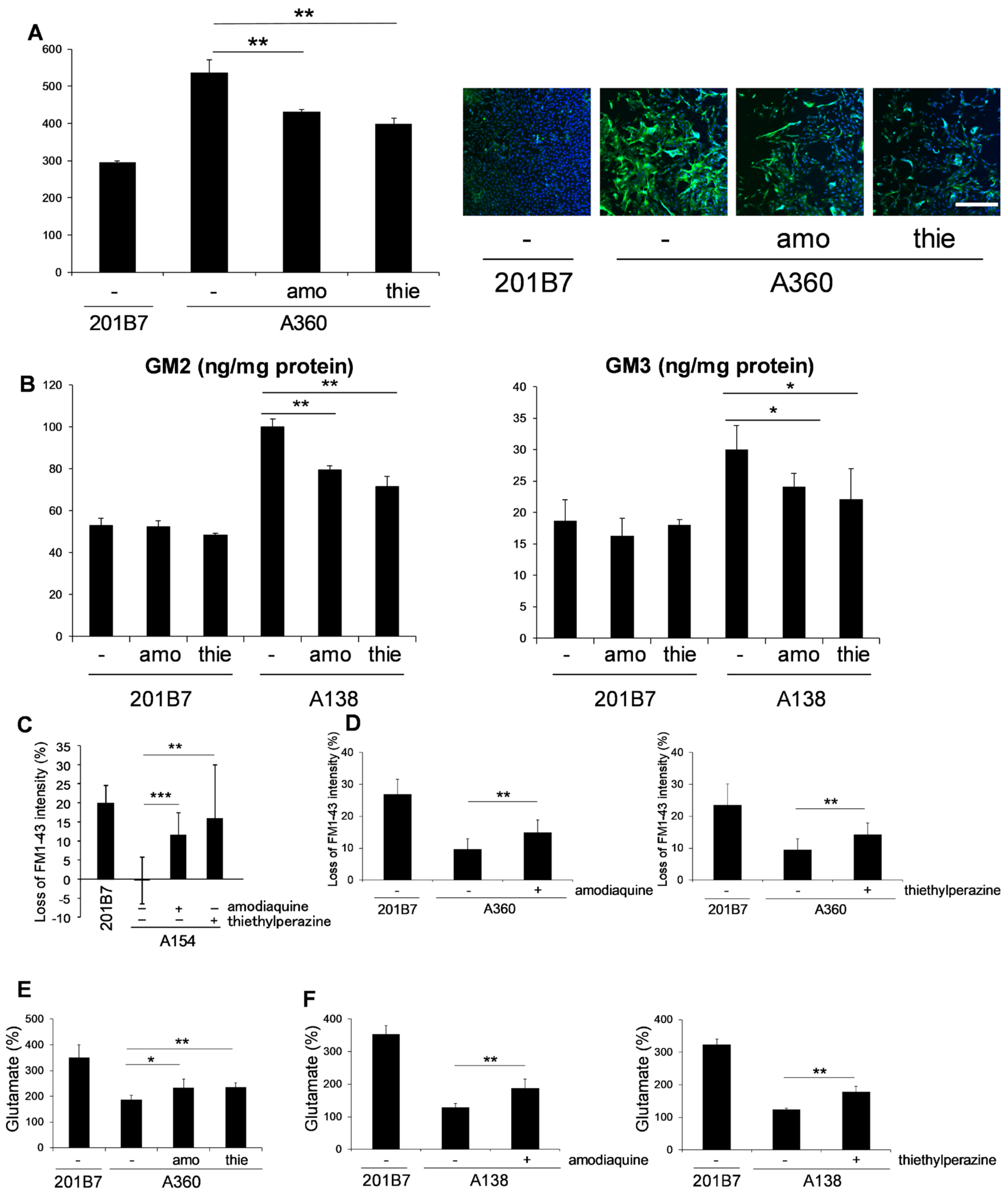

Figure S3. Gangliosides accumulation and in vitro presynaptic function is improved with the treatments of drug candidates (Related to Figure 3)

(A) 201B7 and A360 NSCs were treated with amodiaquine (5 μM) and thiethylperazine (5 μM) for 72 hours. The NSCs were stained with AF488-CTB and the fluorescence intensities were measured using IN CELL ANAYLZER 6000 (Left). $^{**}P < 0.01$ (n = 4, independent samples) Representative images (Right). Scale bars, 100 μm. (B)

Quantification of GM2 and GM3 gangliosides in the NSCs treated with amodiaquine and thiethylperazine. Gangliosides were purified from control (201B7)- and disease (A138 #1-3)-NSCs treated with amodiaquine (5 μM) and thiethylperazine (5 μM) and were measured by LC-MS (mean ± SD from three independent experiments). * $P < 0.05$, ** $P < 0.01$, indicated the treated versus non-treated NSCs, Student's t-test. (C-F) In vitro presynaptic function is restored with long-term treatments of the identified drug candidates. (C, D) Recovery from presynaptic dysfunction in the disease neurons after a long-term treatment with amodiaquine or thiethylperazine in vitro. These two compounds (1.0 μM, respectively) were applied throughout the entire time course of neural differentiation. ** $P < 0.01$, *** $P < 0.001$ (n = 10, n indicates the number of randomly selected buttons). (C); A154 #4-21 on day 74. (D); A360 #1-3 on day 62. (E, F) Treatment with amodiaqine or thiethylperazine restored the decrease of glutamate released from GM1 gangliosidosis-derived neurons. The amount of glutamate was measured by HPLC. The data were displayed as; ratio value (%) of the HK+-stimulated release/basal one, respectively. Basal release (1 min) was collected before the stimulation with HK^+ (50mM, 1 min). *P<0.05., **P<0.01 (n=4, n indicating the number of wells for each experimental condition). (E); A360 #1-3 on day 57. (F); A138 #1-3 on day 69

kajihara *et al* **Figure S4**

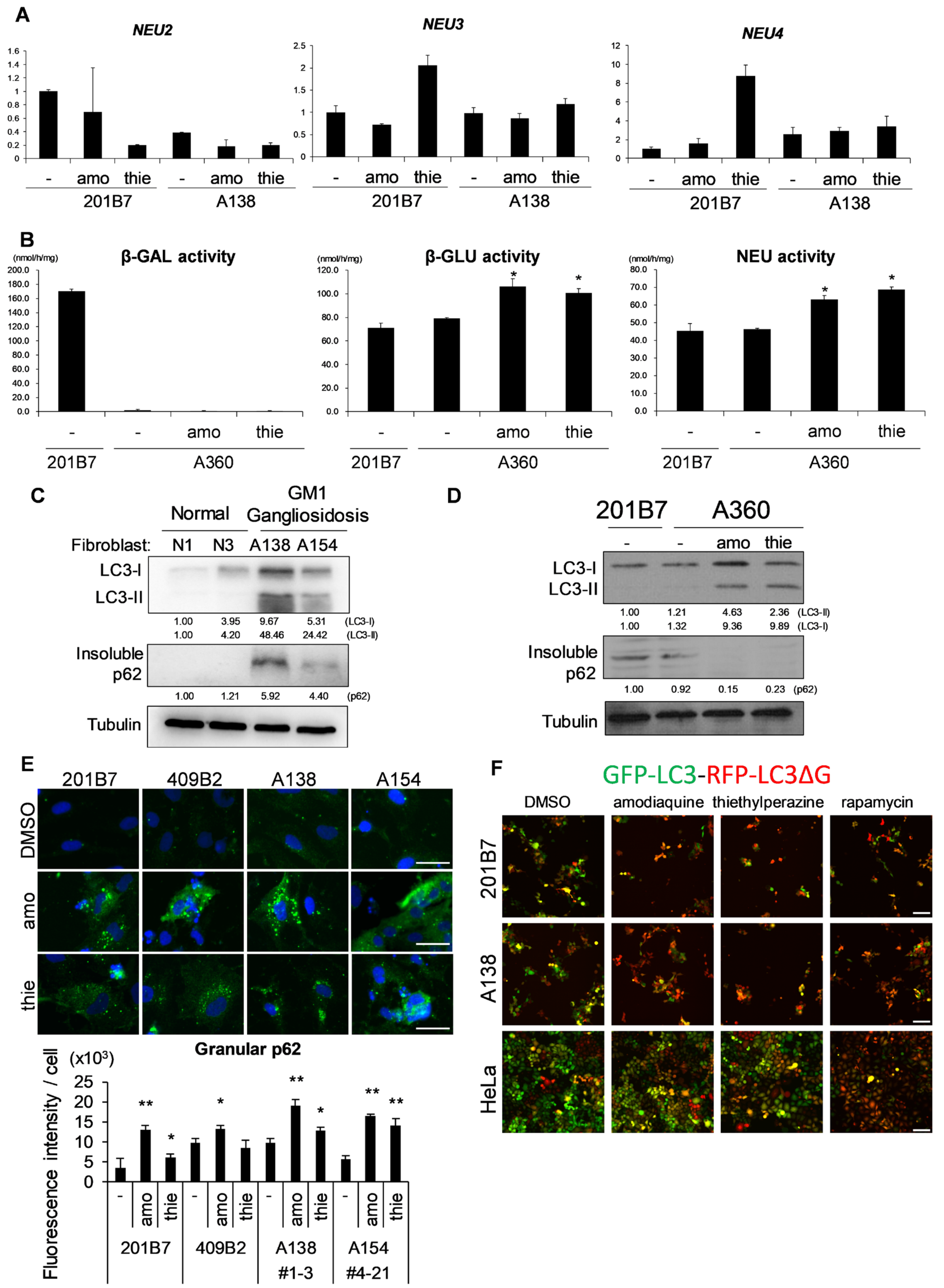

Figure S4. Amodiaquine and thiethylperazine enhance the lysosomal glycosphingolipid degradation and activate autophagy (Related to Figure 4)

(A) Gene expression of NEU family with drug candidate treatments. Normal (201B7) and disease-derived (A138 #1-3) NSCs were treated with 5 μM amodiaquine and 5 μM thiethylperazine for 72 hours. The expression levels of neuraminidases (*NEU2*, *NEU3* and *NEU4*) were measured by RT-qPCR. Data were ratio value normalized to the levels in 201B7-NSCs (-). (mean ± SD from three independent experiments). amo, amodiaquine; thie, thiethylperazine. (B) Enzyme activities of NEU1 and β-GLU were also enhanced with the candidate treatments. NSCs were treated with 5 μM amodiaquine (amo) and 5 μM thiethylperazine (thie) for 48 hours and enzyme activities of β-GAL (left), NEU1 (middle), and β-GLU (right) were measured. Enzyme activities are expressed as nmol/h/mg protein. (mean ± SD from three independent experiments). *$P < 0.05$, ** $P < 0.01$, indicated the treated versus non-treated NSCs, Student's t-test. (C-F) Autophagy in GM1 gangliosidosis-derived cells. (C) Autophagic flux was impaired in GM1 gangliosidosis-derived skin fibroblasts. The protein levels of LC3-II and insoluble p62 were enhanced in GM1 gangliosidosis-derived skin fibroblasts (A138 and A154) compared to normal fibroblasts (N1 and N3). The band intensities were measured using ImageJ software, normalized to each N1 control and described below the images. The results shown are representative of two independent experiments. (D) The protein levels of LC3-II were elevated and those of insoluble p62 decreased in NSCs treated with both compounds. NSCs were treated with 5 μM amodiaquine (amo) and 5 μM thiethylperazine (thie) for 72 hours, western blotting. The band intensities were measured using ImageJ software, normalized to the non-treated 201B7 and described below the images. (E) Granular p62 increased in NSCs treated with amodiaquine and thiethylperazine. NSCs were treated with the candidates as described in Figure 4F. Right: Granular p62 was measured by immunofluorescence staining for p62 followed by the quantification of the fluorescence intensity using IN CELL ANALYZER 6000. *$P < 0.05$, **$P < 0.01$, indicated the treated versus non-treated NSCs, Student's t-test. Left: Representative images. Scale bars, 200 μm. Green, p62, Blue, Hoechst. (F) Representative images corresponding to Figure 4G. Scale bars, 150 μm.

kajihara *et al*　Figure S5

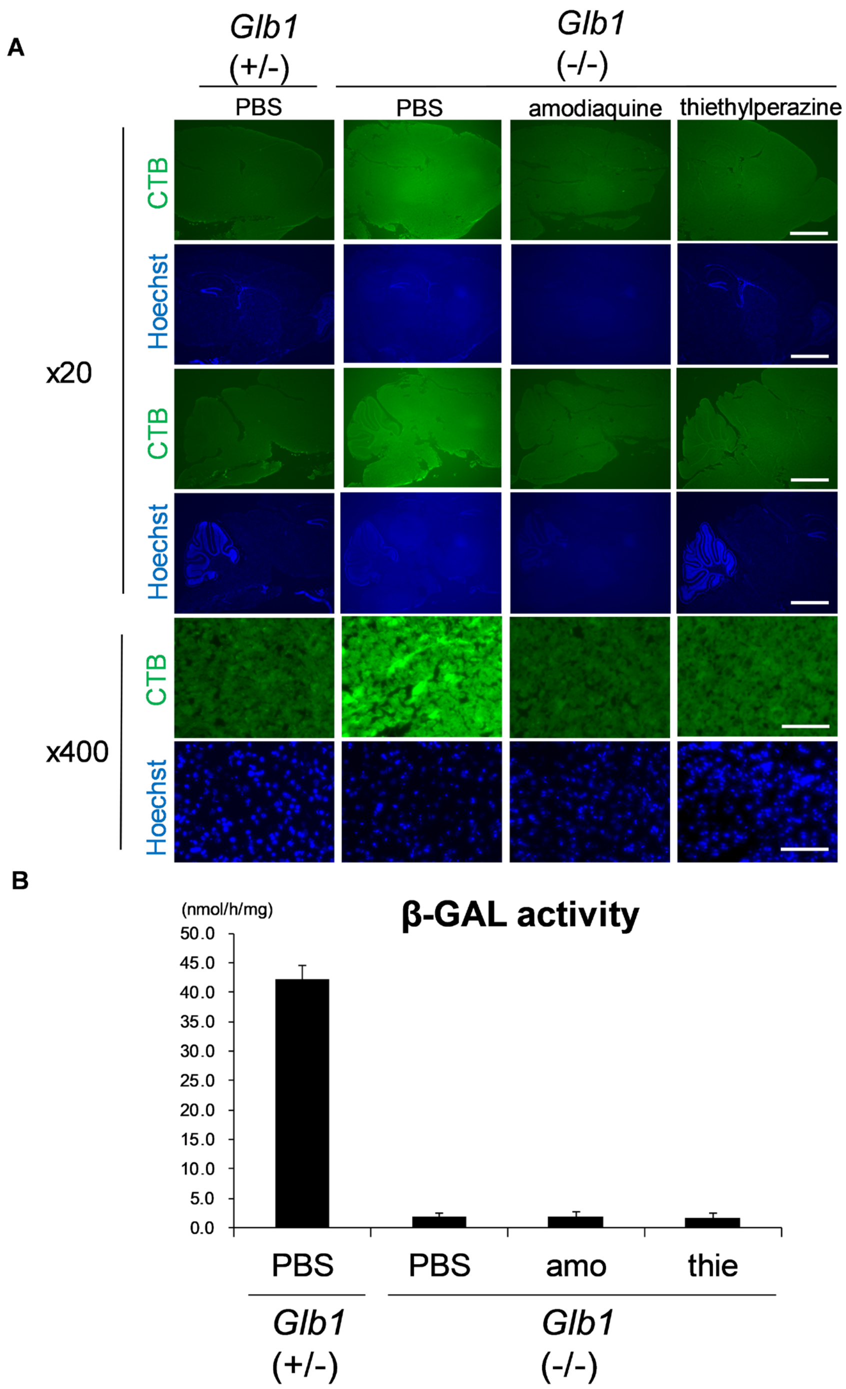

Figure S5. Treatments with amodiaquine and thiethylperazine can reduce GM1 ganglioside accumulations in the brains of model mice (Related to Figure 5)

(A) High and low magnified images corresponding to Figure 5A. Scale bars, 1000 μm (x20), 200 μm (x400). (B) Enzyme activities of β-GAL were not altered with the candidate treatments in *Glb1* KO mice, which were treated as described in Figure 5A.

The brain homogenates were prepared and enzyme activities of β-GAL were measured (mean ± SD from three independent experiments).

kajihara *et al* Figure S6

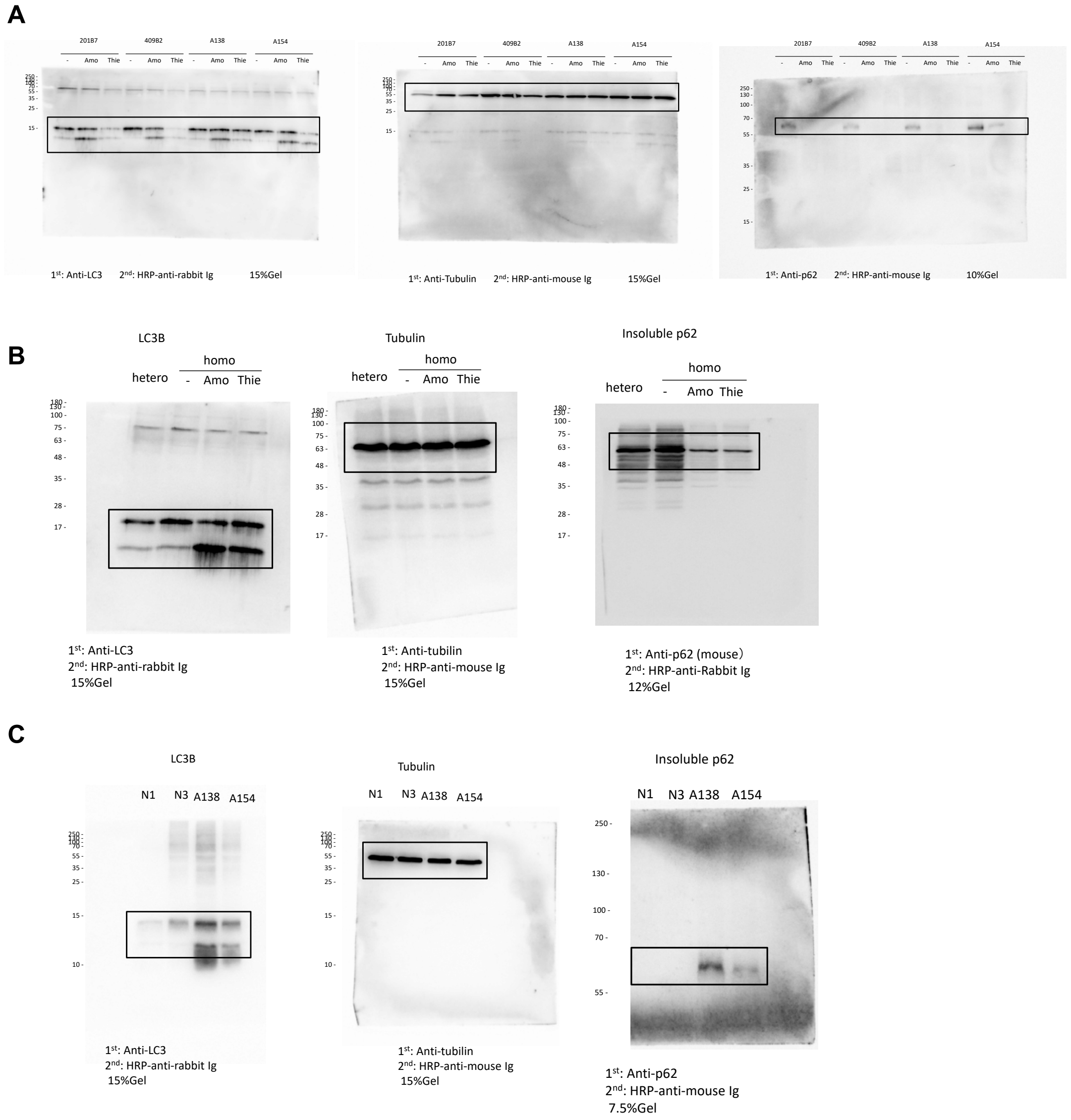

Figure S6. Uncropped images of immunoblots

Raw data corresponding to Figure 4F (**A**), to Figure 5C (**B**), and to Figure S4C (**C**).

Supplemental Experimental Procedure

Skin-derived fibroblasts

Fibroblasts were isolated from explants of skin biopsies obtained from patients following informed consent under protocols approved by the ethics committees of the assigning authors. Skin samples were minced and cultured in Dulbecco's modified essential medium (DMEM, Life Technologies) supplemented with 10% FBS. The subsequent fibroblast cultures were expanded for iPS cell induction as follows.

iPSC generation

We generated iPSCs from human skin-derived fibroblasts as described previously (Fusaki et al., 2009). Briefly, human fibroblast cells were seeded in six-well plates 1 day before infection with Sendai virus (SeV) vectors at 10 multiplicity of infection. After 7 days of culturing, the infected cells were harvested by trypsin and plated on 60-mm dishes coated with mitomycin C-treated mouse embryonic fibroblast feeder cells. From 18 to 25 days after infection, colonies were picked and cultured in human iPSC medium. To remove the Sendai virus, the culturing temperature was increased from 37℃ to 38℃ for 3 days at passage 1 or 2.

iPSC culture

Human iPSCs were cultured on the mitomycin C-treated mouse feeder cells in DMEM/F12 (SIGMA) supplemented with 20% KNOCKOUT serum replacement (KSR, Invitrogen), 2 mM L-glutamine (Life Technologies), 0.1 mM nonessential amino acids (NEAA, SIGMA), 0.1 mM 2-mercaptoethanol (SIGMA), 0.5% penicillin and streptomycin (Nacalai Tesque, Japan), and 5 ng/ml basic fibroblast growth factor (bFGF, WAKO, Japan).

RNA isolation and PCR

Total RNA was isolated using Sepasol® Super G reagent (Nacalai Tesque, Japan), and then transcribed to cDNA using Superscript III (Invitrogen) and random primers (Invitrogen). QuickTaq (TOYOBO, Japan) was used for RT-PCR and THUNDERBIRD qPCR Mix (TOYOBO) for RT-Quantitative PCR (RT-qPCR), as described previously (Soga et al., 2015), and data were analyzed using a StepOnePlus real-time PCR system (Applied Biosystems). Primers used for *OCT3/4*, *SOX2*, *KLF4* and c-*MYC* were designed to detect endogenous gene expression, but not that of transgenes. Nested RT-PCR was performed to detect the SeV genome. Table S1 lists the relevant primer sequences.

Table S1. Primers details for PCR, RT-PCR and RT-qPCR

Genes	Sequences (Forward; F, Reverse; R)	Application
GLB1 (I51T)	F: CTGGTGGACCTGGCTTAGCAATGGT<br>R: GCCAGTCCGGCTCATGACAAGCCCC	PCR
GLB1 (R201C)	F: TAGGAGGTAAACTTTTATGGTGAAT<br>R: ATGAAAACATGAAAAATCTCAATCT	PCR
SeV	F: GGATCACTAGGTGATATCGAGC<br>R: ACCAGACAAGAGTTTAAGAGATATGTATC	RT-PCR
SeV Nested	F: TCGAGCCATATGACAGCTCG<br>R: GAGATATGTATCCTTTTAAATTTTCTTGTCTTCTTG	RT-PCR(nested PCR)
OCT3/4	F: GACAGGGGGAGGGGAGGAGCTAGG<br>R: CTTCCCTCCAACCAGTTGCCCCAAAC	RT-PCR
SOX2	F: GGGAAATGGGAGGGGTGCAAAAGAGG<br>R: TTGCGTGAGTGTGGATGGGATTGGTG	RT-PCR
KLF4	F: GATTACGCGGGCTGCGGCAAAACCTACACA<br>R: TGATTGTAGTGCTTTCTGGCTGGGCTCC	RT-PCR
c-MYC	F: GCGTCCTGGGAAGGGAGATCCGGAGC<br>R: TTGAGGGGCATCGTCGCGGGAGGCTG	RT-PCR
NANOG	F: CAGCCCCGATTCTTCCACCAGTCCC<br>R: CGGAAGATTCCCAGTCGGGTTCACC	RT-PCR
GDF3	F: CTTATGCTACGTAAAGGAGCTGGG<br>R: GTGCCAACCCAGGTCCCGGAAGTT	RT-PCR
REX1	F: CAGATCCTAAACAGCTCGCAGAAT<br>R: GCGTACGCAAATTAAAGTCCAGA	RT-PCR
SALL4	F: AAACCCCAGCACATCAACTC<br>R: GTCATTCCCTGGGTGGTTC	RT-PCR
DNMT3b	F: TGCTGCTCACAGGGCCCGATACTTC<br>R: TCCTTTCGAGCTCAGTGCACCACAAAAC	RT-PCR
b-ACTIN	F: CAACCGCGAGAAGATGAC<br>R: AGGAAGGCTGGAAGAGTG	RT-PCR
PAX6	F: GTCCATCTTTGCTTGGGAAA<br>R: TAGCCAGGTTGCGAAGAACT	qPCR
SOX1	F: GCCCTGAGCCGACTGTGA<br>R: CCGTGAATACGATGAGTG	qPCR
ZNF 521	F: ACCTCCGTGTCCAGTACGAC<br>R: ATGTCAGGGGTTTGTTGAGC	qPCR
NANOG	F: CCAAAGGCAAACAACCCACTT<br>R: CGGGACCTTGTCTTCCTTTTT	qPCR
SYNAPTOTAGMIN I	F: TGAAGTACCTTTTGAACAAATCCA<br>R: GCATCGTTCTTGCCAATCTT	qPCR
SYNAPSIN I	F: TTGCCCAGATGGTTCGACTG<br>R: TGTCAACCTTGACCTTGCCC	qPCR
SYNTAXIN 1A	F: GAAGCACAGTGCCATCCTG<br>R: TGTTTGCTGTCTTCTTTATGTCG	qPCR
GM3 synthase	F: AAGAGACTGCCTTTGACATCC<br>R: CATATCCAAAACCCGCCAAAC	qPCR
GM2 synthase	F: AGAGGGTCAGGCAGATCTCA<br>R: TGCTGTGTTGGTCTGGTAGC	qPCR
GM1 synthase	F: CGCTATTCTTGCTGGGAGAG<br>R: GGGTCTTTAGGGTGAGGTTG	qPCR
GLB1	F: ACGGTGGACTTTGGAACAG<br>R: TGATTGTGGAGTGAGGTTGG	qPCR
HEXA	F: GCTATCGTGACCTGCTTTTCG<br>R: TCTCCAGTGTATGCCGTTTC	qPCR
NEU1	F: TTCTCCAACCCAGCACATC<br>R: ACTGTCTCTTTCCGCCATG	qPCR
NEU2	F: TGCGCAGAGGAGACTACGA<br>R: GTCTGCGCGTCATACAAGG	qPCR
NEU3	F: GGCTTGTTTGGGTGTTTGTT<br>R: TTTTGAATTGGCTTGGGTTC	qPCR
NEU4	F: ACCGCCGAGAGTGTTTTGG<br>R: CGTGGTCATCGCTGTAGAAGG	qPCR
GBA	F: ACAGGATTGCTTCTACTTCAGG<br>R: GGGTCAAAGGAGTCACAGTATG	qPCR
GALC	F: CCTGCATGAAGATGAGCTGTTCACA<br>R: TAGGTACTTGGGAAGGGCTGGGA	qPCR
GAPDH	F: GGCTGGCATTGCCCTCAACG<br>R: AGGGACTCCCCAGCAGTGAG	qPCR

Karyotype analysis

G band chromosome analyses were performed by Nihon Gene Research Laboratories Inc. (Sendai, Japan), according to the manufacturer's protocol.

Teratoma formation

The patient-derived iPSC lines grown on MEF feeder layers were collected by treatment with collagenase IV and injected into the testes of NOJ（NOD／Scid／Jak3 KO） immunodeficient mice (Okada et al., 2008). Palpable tumors were observed at 12-16 weeks after injection. Tumor samples were collected, fixed in 10% formalin, and processed for paraffin-embedding and hematoxylin-eosin staining following standard procedures.

Genomic Sequencing

The mutations of *GLB1* gene in the patients-derived fibroblasts were confirmed by direct sequencing. The genomic DNAs extracted were amplified by PCR and the resultant PCR products were sequenced by ABI PRISM™ 310 Genetic Analyzer (Applied Biosystems) as described previously (Soga et al., 2015) .

Western blot analysis

Lysates were prepared in RIPA buffer (50 mM Tris-HCl [pH 8], 150 mM NaCl, 0.5% sodium deoxycholate, 0.1% SDS, and 1% Nonidet P-40) with freshly added protease inhibitors and phosphatase inhibitors. To detect insoluble p62, RIPA-insoluble pellets were dissolved in SDS sample buffer. Western blot analysis was performed as described previously (Kajihara et al., 2014). Briefly, samples were separated by SDS-PAGE and transferred to Immobilon-P membranes (Millipore), which were incubated for 1 h in blocking buffer (TBS containing 0.1% Tween 20 and 5% nonfat dry milk) followed by overnight incubation with primary antibody in blocking buffer. After extensive washing, the blot was incubated with a relevant secondary antibody for 1 h and processed using ECL reagents (GE Healthcare, Buckinghamshire, U.K.) according to the manufacturer's instructions. In order to compare the particular protein band intensities, the obtained scanned images were analyzed using ImageJ software (NIH, Bethesda, MA, USA). The relative band intensities were expressed in arbitrary units in relation to the appropriate reference sample band intensity. Antibodies and working conditions are listed in Table S2.

Table S2. Antibodies details for immunostaining and immunoblotting

Antibodies and other probes	Species	Working Dilution	Vendor (Catalog number)	Application
Anti-SSEA4	Mouse	1:500	MILLIPORE (MAB4304)	immunostaining
Anti-TRA-1-60	Mouse	1:500	MILLIPORE (MAB4360)	immunostaining
Anti-NANOG	Goat	1:1000	R&D systems (4903S)	immunostaining
Anti-OCT3/4	Mouse	1:500	Santa Cruz (sc-5279)	immunostaining
Anti-SOX1	Rabbit	1:100	Cell Signaling Technology	immunostaining
Anti-PAX6	Rabbit	1:1000	Covance (PRB-278P)	immunostaining
Anti-NESTIN	Mouse	1:500	R&D systems (MAB1259)	immunostaining
Cholera Toxin Subunit B (Recombinant), Alexa Fluor 488		1:2500	Molecular Probes (C34775)	immunostaining
Anti-LC3	Rabbit	1:500	Cell Signaling Technology	immunoblot
Anti-p62 (Human)	Mouse	1:500<br>1:200	MBL (M162-3)	immunoblot<br>immunostaining
Anti-p62 (Mouse)	Rabbit	1:1000	MBL (PM045)	immunoblot
Anti-MAP2	Rabbit	1:500	Millipore (Ab5622)	immunostaining
Anti-SYNAPSIN I	Mouse	1:500	Synaptic systems (106001)	immunostaining
Anti-mouse HRP	Goat	1:3000	Bio rad (170-6516)	immunoblot
Anti-Rabbit HRP	Goat	1:3000	Bio rad (170-6515)	immunoblot
Alexa 488-conjugated goat anti-mouse IgG	Goat	1:1000	Invitrogen (A11029)	immunostaining
Alexa 488-conjugated donkey anti-rabbit IgG	Donkey	1:1000	Invitrogen (A21206)	immunostaining
Alexa 594-conjugated goat anti-mouse IgG	Goat	1:1000	Invitrogen (A11005)	immunostaining
Hoechst 33258		1:2000	Invitrogen (H3569)	immunostaining

Cell staining and immunocytochemistry

Alkaline phosphatase staining was performed using the Leukocyte Alkaline Phosphatase kit (SIGMA). For immunocytochemistry, cells were fixed with 4% paraformaldehyde in phosphate-buffered saline (PBS) for 30 min at 4℃, and then treated with 0.2% Triton X-100 for 15 min at room temperature (RT) to localize nuclear proteins. The cells were then washed three times with blocking buffer (PBS containing 2% FBS) and incubated overnight at 4℃ with primary antibodies diluted in blocking buffer. The bound primary antibodies were detected by incubation in Alexa-Fluor-conjugated secondary antibodies for 1 h, and nuclei were counterstained with 1 μg/ml Hoechst 33258 (Invitrogen). Table S2 lists the antibodies and conditions used in these experiments.

Neuralization of iPSCs

We differentiated iPSCs into neural stem cells (NSCs) using Gibco PSC neural induction medium (Life Technologies) according to the manufacturer's protocol with minor modification (Yan et al., 2013). Semi-confluent iPSCs cultured on MEF feeder cells were detached with collagenase IV and re-plated onto Geltrex (Life Technologies)-coated plates with Neural Induction Medium [NIM, consisting of Neurobasal Medium (NM: from Life Technologies) and 1/100 volume of Neural Induction Supplement (NIS: Life Technologies)], containing Y-27632 (10 μM). Medium was changed with fresh NIM (without Y-27632) on days 3 and 5 after plating. At day 7, colonies of differentiated NSCs were re-plated for further maintenance (48 hours), before detachment using Accutase (Life Technologies) and gentle pipetting to dissociate the colonies into single cells. The dissociated NSCs were then cultured on Geltrex-coated dishes with Neural Expansion Medium (NEM) comprising 50% Advanced DMEM/F12 (Life Technologies), 50% NM, and 1/100 volume of NIS, supplemented with Y-27632 [10 μM, until passage 2 (P2)]. The NSCs were then passaged every 5-9 days and used for neural differentiation up to P10. For neuralization, the NSCs were dissociated for plating on polyethyleneimine (PEI)-coated glass-bottom dishes (Matsunami Glass Ind.) or astrocyte feeders prepared from mouse cortex with NM containing a 1/50 volume of B-27 supplement (Life Technologies) and 0.5 mM L-glutamate. Three weeks later, the culture medium was replaced with DMEM containing 20% KSR, and fresh medium was replaced once or twice a week. For immunocytochemistry and imaging assays, we used neurons maintained for 56-85 days from neuralization. In some experiments, recombinant human β-GAL (R&D systems, 200nM) was added to the culture medium during neuralization

FM 1-43 imaging

FM 1-43 imaging was used to monitor exocytotic activity in the iPSC-derived neurons, as described previously (Numakawa et al., 1999). Briefly, the neuralizing culture medium was replaced with artificial cerebrospinal fluid (ACSF; 125 mM NaCl, 2.5 mM KCl, 2 mM $CaCl_2$, 1 mM $MgCl_2$, 1.25 mM NaH_2PO_4, 25 mM $NaHCO_3$, 25 mM glucose and 5 μM FM 1-43) for 30 min at 37℃. After several washes with fresh ACSF without the FM dye, the FM 1-43 concentration in cells was monitored using a fluorescence microscope (DMi8, Leica). Neurons were irradiated with an excitation wavelength of 488 nm and emitted fluorescence was detected at 520 nm. HK+ (50 mM KCl) ACSF solution was used to trigger membrane depolarization, and decreases in the cellular FM 1-43 fluorescence intensity were determined by comparing the stimulation (2 sec after HK+ stimulation) and basal (2 sec before the stimulation) intensities. The intensity of FM emission was captured every 1 sec and stored as stacked images using the LAS X imaging system (Leica). To confirm reproducibility, the imaging experiment was carried out at least 3-5 times on independent cultures.

Measurement of lysosomal enzyme activities

Lysosomal enzyme activities in cell lysates were measured fluorometrically with 4-methylumbelliferone (4MU) substrates including 4-methylumbelliferyl-β-D-galactopyranoside (Sigma-Aldrich) for β-GAL, 4-methylumbelliferyl-α-D-N-acetylneuraminic acid (Sigma-Aldrich) for NEU1, and 4-methylumbelliferyl-β-D-glucopyranoside (Sigma-Aldrich) for β-GLU, as described previously (Okumiya et al., 2006). Briefly, 10 μl of the cell homogenate was added to 60 μl of the 4MU substrate solution in a 96-well, white microtiter plate (OptiPlate-96, Packard, Meriden, CT). The reaction mixture was then incubated at 37°C for 1 h, and the reaction was stopped by addition of 200 μl of 0.2M Glycine-NaOH buffer pH10.7 with 0.1% Triton X-100. Fluorescence intensities were measured with a fluorescence plate reader (excitation 360 nm; emission 450 nm; Infinite F200 Pro, TECAN Japan, Kawasaki, Japan), and corrected for substrate blank without cell homogenates. To calibrate the measurement of liberated 4MU, we used 125 μmol/l of 4MU in 20 mmol/l sodium phosphate buffer at pH 7.0 instead of the sample. The enzyme activity was expressed as nanomoles of 4MU released per hour per milligram cellular protein (nmol/h/mg protein).

Ganglioside purification and quantification by LC-MS

Gangliosides were extracted according to the procedure developed by Svennerholm and

Fredman (Fong et al., 2009). Briefly, cells or portions of mice brains were resuspended in water, lysed by freeze thawing, and then extracted twice with chloroform/methanol/water (4:8:3 v/v/v). The upper layers were pooled, and distilled water was added to give a final chloroform/methanol/water ratio of 1:2:1.4 (v/v/v), before centrifuging for 30 min at 2000 x g. Additional methanol (half of the lower phase volume) and 0.01 M KCl (20% of the total volume of the lower phase plus added methanol) were added to the lower phase, vortexed, and then centrifuged for 30 min at 2000 x g. The two upper phases were combined and evaporated to dryness in a rotary evaporator. The dried residue was dissolved in 2 ml of KCl 0.1 M, and recycled 3 times on a Sepak C18 column (1 ml). The bound material was eluted with 15 ml of methanol and 5 ml of chloroform/methanol (2:1 v/v). The eluates were evaporated to dryness in a rotary evaporator, and resuspended in 50% methanol as samples for HPLC analysis using an Agilent 1100 series HPLC system (Santa Clara, CA) comprising a binary pump, degasser, column heater (60°C), and refrigerated auto-sampler (5°C). Samples or standards (10 μl) were separated using an APS-2 hypersil hydrophilic column (150 mm x 2.1 mm, 3 μm, Thermo Scientific, Waltham, MA) coupled to an APS-2 guard column (10 mm x 2.1 mm inner diameter) with an acetonitrile/ammonium acetate buffer gradient. Eluants from the HPLC system were introduced into an Agilent 6470 Triple Quadrupole Mass Spectrometer to quantify each class of ganglioside.

Neuronal survival assay and neurite length measurement

NSC lines (201B7, 409B2, A138 #1-3, A154 #4-21) were plated at 1.6 x 10^4 cells/well in PEI-coated 96-well plates, and then cultured for neuronal differentiation for 66 days. GM1 exposure was performed during day 46 to 66, and then again at day 96. At day 66, cells were fixed with 4% paraformaldehyde for 20 min at room temperature. After blocking and permeabilization with PBS containing 1% BSA and 0.1% Triton X-100 for 30 min, cells were incubated with anti-MAP2 antibody (1:500, ab5622, Millipore) at 4°C overnight. Cells were then washed with PBS and stained with Alexa Fluor 488 donkey anti-rabbit IgG (1:1000, Life Technologies) and Hoechst 33342 (1 μg/ml, Invitrogen) for 1 hour at room temperature. Fluorescence of MAP2 and nuclear staining was visualized using an IN Cell Analyzer 6000, and quantified as number of MAP2-positive cells per field and length of MAP2-positive neurites using Developer Toolbox software. To assess cell viability, neuronal survival was shown as the relative value of MAP2-positive cells at each GM1 concentration compared to the non-treatment group for each cell line.

Treatment of GM1 gangliosidosis model mice with hit compounds

Glb1 gene KO mice (BKO mice) were bred and kept in specific pathogen-free conditions in the Center for Animal Resources and Development (CARD), Kumamoto University. PBS, 40 mg/kg amodiaquine, or 6 mg/kg thiethylperazine was intraperitoneally injected into homozygous BKO mice twice a day from 9 to 15 days of age (n = 4 each). At 15 days of age, the mice were sacrificed and the brain samples were collected for further experiments. For histological analysis of GM1-ganglioside storage, frozen sections of the brain samples were fixed for 10 min in 4% paraformaldehyde in PBS, pH 7.4 at room temperature. After blocking with 1% BSA in PBS for 10 min at room temperature, sections were incubated for 1 h at room temperature with Alexa Fluor 488-conjugated cholera toxin B in PBS, (1:200; Molecular Probes). After 3 × 10-min washes in PBS they were counterstained with Hoechst 33258.

Primary culture of mouse neurons

Cerebral cortical tissues of 1- or 2-day-old controls (*Glb1* +/-) and BKO mice (*Glb1* -/-) were dissected for culturing cortical neurons as previously reported (Numakawa et al., 2009). Briefly, dissected brain tissue was digested with 0.25% trypsin at 37° C for 30 min, and then cortical cells were dissociated by gentle pipetting, followed by plating on polyethyleneimine-coated glass-bottom dishes at 5 x 10^5 cells/cm^2. The cortical cell cultures were maintained for 22 days with neurobasal media containing B27 prior to FM imaging.

Measurement of autophagic flux

To generate stable cell lines expressing an autophagic flux probe (GFP-LC3-RFP-LC3ΔG), NSCs and HeLa cells were retrovirally transfected with pMRX-IP-GFP-LC3-RFP-LC3ΔG (Addgene, 84572) as previously described (Kaizuka et al., 2016). The cells were plated at 5 x 10^4 cells/well in 96-well plates coated with Geltrex and then treated with 5 μM amodiaquine, 5 μM thiethylperazine or 1 μM rapamycin for 72 hours. The fluorescence intensities of GFP and RFP were measured using IN Cell Analyzer 6000 (GE Healthcare). The data was analyzed with Developer Toolbox software (GE Healthcare). Autophagic flux was estimated by calculating the GFP/RFP signal ratio.

LDH leakage assay

In order to evaluate the possible cytotoxicity of GM1 ganglioside, lactate dehydrogenase (LDH) leaking from the neurons into the culture medium was measured

by Cytotoxicity LDH Assay Kit-WST (Dojindo) according to the manufacturer's protocol with minor modification. Following exposure to 200nM GM1 ganglioside during day 46 to 66 of neuronal differentiations, 50 μl culture medium was transferred from 96 well culture plate to a clear 96 well plate. After adding 50 μl Working Solution to each wells, plate was incubated for 30 min at the room temperature. Then 25 μl Stop Solution was applied to each wells and the absorbance at 450 nm was measured by a microplate reader.

Measurement of glutamate

The amount of released glutamate, an excitatory neurotransmitter, from differentiated neurons was measured with high performance liquid chromatography (HPLC; Shimazu Co, Osaka, Japan). First, neuronal cultures were gently washed 4-6 times using assay buffer (ACSF; 125 mM NaCl, 2.5 mM KCl, 2 mM $CaCl_2$, 1 mM $MgCl_2$, 1.25 mM NaH2PO_4, 25 mM $NaHCO_3$, and 25 mM glucose). Then, fresh assay buffer was applied for 1min and collected without a stimulation as the basal release of glutamate. Afterword, secondary incubation with assay buffer containing KCl (50 mM) was performed for 1 min. Before the analysis with HPLC, amino acids in the collected samples were derivated with o-phthalaldehyde and -2-mercaptoethanol. The 12- or 24-well plate were used for cell cultures to carry out the determination of glutamate.

Supplemental References

Fong, B., Norris, C., Lowe, E., and McJarrow, P. (2009). Liquid chromatography-high-resolution mass spectrometry for quantitative analysis of gangliosides. Lipids *44*, 867-874.

Fusaki, N., Ban, H., Nishiyama, A., Saeki, K., and Hasegawa, M. (2009). Efficient induction of transgene-free human pluripotent stem cells using a vector based on Sendai virus, an RNA virus that does not integrate into the host genome. Proceedings of the Japan Academy Series B, Physical and biological sciences *85*, 348-362.

Kaizuka, T., Morishita, H., Hama, Y., Tsukamoto, S., Matsui, T., Toyota, Y., Kodama, A., Ishihara, T., Mizushima, T., and Mizushima, N. (2016). An Autophagic Flux Probe that Releases an Internal Control. Mol Cell *64*, 835-849.

Kajihara, R., Sakamoto, H., Tanabe, K., Takemoto, K., Tasaki, M., Ando, Y., and Inui, S. (2014). Protein phosphatase 6 controls BCR-induced apoptosis of WEHI-231 cells by regulating ubiquitination of Bcl-xL. Journal of immunology (Baltimore, Md : 1950) *192*, 5720-5729.

Numakawa, T., Kumamaru, E., Adachi, N., Yagasaki, Y., Izumi, A., and Kunugi, H. (2009). Glucocorticoid receptor interaction with TrkB promotes BDNF-triggered PLC-gamma signaling for glutamate release via a glutamate transporter. Proc Natl Acad Sci U S A *106*, 647-652.

Numakawa, T., Takei, N., Yamagishi, S., Sakai, N., and Hatanaka, H. (1999). Neurotrophin-elicited short-term glutamate release from cultured cerebellar granule neurons. Brain Res *842*, 431-438.

Okada, S., Harada, H., Ito, T., Saito, T., and Suzu, S. (2008). Early development of human hematopoietic and acquired immune systems in new born NOD/Scid/Jak3null mice intrahepatic engrafted with cord blood-derived CD34 + cells. International journal of hematology *88*, 476-482.

Okumiya, T., Keulemans, J.L., Kroos, M.A., Van der Beek, N.M., Boer, M.A., Takeuchi, H., Van Diggelen, O.P., and Reuser, A.J. (2006). A new diagnostic assay for glycogen storage disease type II in mixed leukocytes. Mol Genet Metab *88*, 22-28.

Soga, M., Ishitsuka, Y., Hamasaki, M., Yoneda, K., Furuya, H., Matsuo, M., Ihn, H., Fusaki, N., Nakamura, K., Nakagata, N.*, et al.* (2015). HPGCD outperforms HPBCD as a potential treatment for Niemann-Pick disease type C during disease modeling with iPS cells. Stem Cells *33*, 1075-1088.

Yan, Y., Shin, S., Jha, B.S., Liu, Q., Sheng, J., Li, F., Zhan, M., Davis, J., Bharti, K., Zeng, X.*, et al.* (2013). Efficient and rapid derivation of primitive neural stem cells and generation of brain subtype neurons from human pluripotent stem cells. Stem Cells Transl Med *2*, 862-870.
