# Supplementary material for: Novel Drug Candidates Improve Ganglioside Accumulation and Neural Dysfunction in GM1 Gangliosidosis Models with Autophagy Activation
Source: Stem Cell Reports. 2020 Apr 16;14(5):909–23. doi: 10.1016/j.stemcr.2020.03.012 (PMC7220856; doi:10.1016/j.stemcr.2020.03.012)
Supplement: Document S2. Article plus Supplemental Information [file mmc2.pdf]

Stem Cell Reports
Article
OPEN ACCESS

Novel Drug Candidates Improve Ganglioside Accumulation and Neural Dysfunction in GM1 Gangliosidosis Models with Autophagy Activation

Ryutaro Kajihara,[1,2] Tadahiro Numakawa,[1] Haruki Odaka,[1] Yuji Yaginuma,[3] Noemi Fusaki,[4] Toshika Okumiya,[2] Hirokazu Furuya,[5] Seiji Inui,[2] and Takumi Era[1,*]

[1]Department of Cell Modulation, Institute of Molecular Embryology and Genetics, Kumamoto University, 2-2-1 Honjo, Chuo-ku, Kumamoto 860-0811, Japan
[2]Department of Biomedical Laboratory Sciences, Faculty of Life Sciences, Kumamoto University, Kumamoto 862-0976, Japan
[3]Department of Morphological and Physiological Sciences, Faculty of Life Sciences, Kumamoto University, Kumamoto 862-0976, Japan
[4]University Research Administration Center, Office of Research Promotion, Tohoku University, Tohoku 980-8577, Japan
[5]Department of Neurology, Kochi Medical School, Kochi University, Kochi 783-8505, Japan
*Correspondence: tera@kumamoto-u.ac.jp
https://doi.org/10.1016/j.stemcr.2020.03.012

SUMMARY

GM1 gangliosidosis is a lysosomal storage disease caused by loss of lysosomal β-galactosidase activity and characterized by progressive neurodegeneration due to massive accumulation of GM1 ganglioside in the brain. Here, we generated induced pluripotent stem cells (iPSCs) derived from patients with GM1 gangliosidosis, and the resultant neurons showed impaired neurotransmitter release as a presynaptic function and accumulation of GM1 ganglioside. Treatment of normal neurons with GM1 ganglioside also disturbed presynaptic function. A high-content drug-screening system was then established and identified two compounds as drug candidates for GM1 gangliosidosis. Treatment of the patient-derived neurons with the candidate agents activated autophagy pathways, reducing GM1 ganglioside accumulation *in vitro* and *in vivo*, and restoring the presynaptic dysfunction. Our findings thus demonstrated the potential value of patient-derived iPSC lines as cellular models of GM1 gangliosidosis and revealed two potential therapeutic agents for future clinical application.

INTRODUCTION

GM1 gangliosidosis is a lysosomal storage disorder characterized by abnormal accumulation of GM1 ganglioside. The main clinical feature of the disease is neural dysfunction due to massive GM1 ganglioside deposition in the central nervous system (CNS) (Sandhoff and Harzer, 2013). This abnormal deposition is caused by a deficiency in lysosomal β-galactosidase (β-GAL) activity that limits the patients' ability to degrade GM1 ganglioside in lysosomes and eventually leads to excessive GM1 ganglioside accumulation, particularly in the brain. While it remains unclear how this abnormal storage of GM1 ganglioside directly affects neural function, previous studies proposed several pathological pathways, including the unfolded protein response (UPR), endoplasmic reticulum calcium signaling, autophagy, and inflammasome activation (Sano et al., 2009; Son et al., 2015; Takamura et al., 2008; Tessitore et al., 2004).

Depending on the age of onset, GM1gangliosidosis is classified into three forms: infantile, juvenile, and adult forms. The life prognosis is poorer with early onset than with late onset. The infantile form develops by the age of 6 months and exhibits the most severe symptoms, such as developmental regression, seizures, eye abnormality, and hepatosplenomegaly. The juvenile form develops after 5 years and exhibits developmental regression. The adult form has milder symptoms such as dystonia and progresses chronically (Sandhoff and Harzer, 2013).

Several therapeutic approaches have been tried to treat the disease, such as bone marrow transplantation, gene therapy, substrate reduction therapy, and chemical chaperone therapy (Baek et al., 2010; Elliot-Smith et al., 2008; Shield et al., 2005; Suzuki et al., 2012); however, these strategies have had limited effects, and there remains no effective treatment or candidate drugs on the horizon for GM1 gangliosidosis patients. Disease models in mice and using patient-derived fibroblasts have been developed to understand the pathogenesis of GM1 gangliosidosis (Sano et al., 2009; Tessitore et al., 2004); however, efforts to generate human neural cells able to simulate the disease have proven unsuccessful. For instance, human infantile GM1 gangliosidosis exhibits early onset and poor prognosis; it develops at 0–6 months of age and patients die around the age of 1 year (Brunetti-Pierri and Scaglia, 2008), while β-GAL null mice (BKO mice), a popular mouse model for GM1 gangliosidosis, develop disease around 4 months of age and live up to 10 months of age, corresponding to over 20 years of age in humans (Matsuda et al., 1997). In addition, unlike human infantile patients, hepatosplenomegaly and skeletal dysplasia are not observed in BKO mice (Matsuda et al., 1997). Thus, the mouse model also does not exactly recapitulate the human disease.

Induced pluripotent stem cells (iPSCs) are artificially generated from human somatic cells with overexpression of four reprogramming factors: OCT3/4, SOX2, KLF4, and cMYC (Takahashi et al., 2007). Human iPSCs can easily proliferate and have the potential to generate various cell types

This is an open access article under the CC BY license (http://creativecommons.org/licenses/by/4.0/).

in vitro. Based on these attributes, patient-derived iPSCs have proven powerful tools in establishing human disease models, pathological research, and new drug development (Latour et al., 2019; Soga et al., 2015).

The present study sought to elucidate the pathological mechanisms of neural dysfunction in GM1 gangliosidosis and, based on the results, to identify therapeutic candidates. To approach this aim, we generated patient-derived iPSCs, characterized the impaired function of neural cells differentiated from the iPSCs, and established a high-content drug-screening system.

RESULTS

Establishment of iPSC Lines from GM1 Gangliosidosis Patients

To investigate possible pathological events and drug targets in GM1 gangliosidosis, we generated iPSCs (GM1-iPSCs) from skin fibroblasts of three patients carrying homozygous mutations in β-GAL, R201C in A138 (juvenile form) and A360 (juvenile form), and I51T in A154 (adult form) (Figure S1A). A previous study showed that they were causative mutations in GM1 gangliosidosis (Morita et al., 2009). Six clonal iPSC lines established from three affected patients (1-1 and 1-3 from patient A138, 2-19 and 4-21 from patient A154, and 1-3 and 1-4 from patient A360) exhibited embryonic stem cell-like morphology and expressed a set of pluripotent markers determined by immunostaining and PCR analyses (Figures S1B and S1C). Subsequent examination of the iPSCs for differentiation capacity using a teratoma formation assay and histological analysis revealed descendants of all three germ cell layers, including columnar epithelia, melanin pigment-containing cells, cartilage, muscle, and various glandular structures (Figure S1D). We also confirmed that the iPSC lines derived from patients A138 and A360 had a normal karyotype of 46XX, whereas those from patient A154 (both 2-19 and 4-21) showed an abnormal karyotype of 46XX, add(9) (p24, arrow), and the abnormal karyotypes can affect the results in further experiments (Figure S1E). We therefore mainly used the A138 iPSC line and confirmed the results using A154 or A360 iPSC lines. The A154 patient was diagnosed as a different clinical form with a different mutation in the *GLB1* gene, which encodes β-GAL protein, from other two. Thus, using the A154-derived iPSCs, we sometimes confirmed the results from other iPSCs. In this study, 201B7 and 409B2 iPSC lines were used as normal controls (Okita et al., 2011; Takahashi et al., 2007).

GM Gangliosides Accumulated in NSCs Derived from GM1-iPSCs

One of the main clinical features reported in GM1 gangliosidosis is neurodegeneration (Brunetti-Pierri and Scaglia, 2008; Sandhoff and Harzer, 2013), implicating neural cells, including neural stem cells (NSCs) and differentiated neurons as a profoundly affected cell population in the disease. To assess the cellular phenotypes present in GM1 gangliosidosis, we first differentiated the disease-derived iPSC lines into NSCs expressing specific markers, SOX1, PAX6, and NESTIN (Figure S1F). Then, we examined possible differences in neural induction efficiency between disease and control (201B7 and 409B2) iPSCs by comparing mRNA expression levels of NSC markers (*SOX1*, *ZNF521*, and *PAX6*) by qRT-PCR analysis, but found no differences between the disease and control cells (Figure S1G). Both control and disease NSCs also showed downregulation of *NANOG*, an iPSC marker (Figure S1G).

We next investigated whether the patient-derived NSCs could mimic a disease-specific phenotype. In GM1 gangliosidosis, deficient lysosomal β-GAL activity due to a mutation in the *GLB1* gene causes accumulation of GM1 ganglioside. We therefore used an artificial β-GAL substrate (4-methylumbelliferyl-β-D-galactoside) to assess the enzymatic activities of both normal and disease-derived NSCs. GM1 gangliosidosis-derived NSCs showed markedly lower β-GAL activity compared with control NSCs (Figure 1A). We also visualized the intracellular GM1 ganglioside make-up in the NSCs using Alexa Fluor 488-conjugated cholera toxin subunit-B (AF488-CTB), which specifically binds to the GM1 ganglioside (Iwamasa et al., 1987). Positive staining was negligible in the control NSCs, whereas the disease-derived NSCs showed a strong signal for AF488-CTB (Figures 1B and S1H), and liquid chromatography-mass spectrometry (LC-MS) analysis confirmed the increased levels of GM1 ganglioside (Figure 1C). Interestingly, GM2 and GM3 gangliosides were also accumulated in the disease-derived NSCs (Figure 1C), and together, these results suggest that the NSCs derived from GM1-iPSCs mirror the biochemical phenotype of GM1 gangliosidosis.

Accumulation of GM1 Ganglioside Disturbs Presynaptic Function *In Vitro*

We next examined *in vitro* synaptic function in the neuralized NSCs after long-term maintenance with neural differentiation medium. MAP2-positive differentiated neurons derived from both control (201B7) and disease (A138) NSCs showed many SYNAPSIN I-positive synaptic puncta (Figure 2A), and similarities in neuronal composition among the control and disease-derived cultures, including glutamatergic (*vGLUT1*) and GABAergic (*GAD67*) populations, were determined by qPCR analysis (Figure S2A). Similar to the NSCs, differentiated neurons derived from GM1-iPSCs exhibited increased accumulation of GM1, 2, and 3 gangliosides compared with the control neurons (Figure 2B). To explore the possible influence of this increased intracellular GM1 ganglioside on neuronal

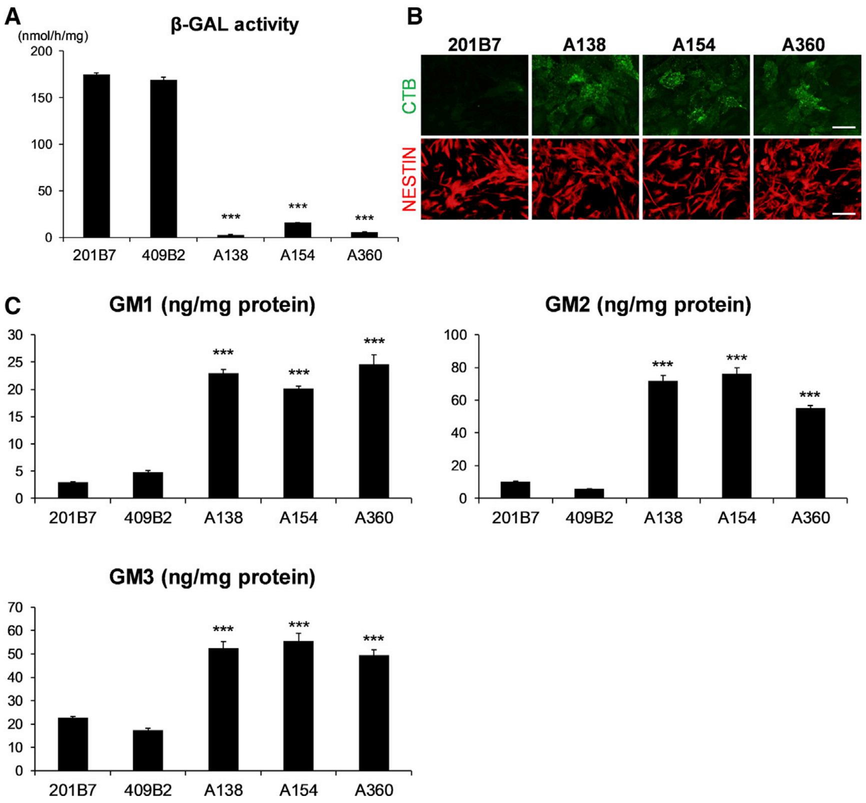

Figure 1. Biochemical Phenotype of GM1-iPSC-Derived Neural Stem Cells

(A) β-GAL activities in normal and GM1 gangliosidosis-derived NSCs. The NSCs were generated from disease-derived iPSCs (A138 1-3, A154 4-21, and A360 1-3) and normal iPSCs (201B7). The NSC lysates were incubated with artificial β-GAL substrate (4-methylumbelliferyl-β-D-galactoside), and enzyme activities were measured based on fluorescence intensities. The activities are expressed as nmol/h/mg protein. The bars represent the mean ± SD from three independent experiments. ***$p < 0.001$, disease versus normal, Student's t test.

(B) CTB staining of iPSC-derived NSCs. Control (201B7) and disease (A138 1-3, A154 4-21, and A360 1-3)-derived NSCs were also stained with anti-NESTIN antibody (red color) followed by Alexa Fluor 488-CTB (green color). Scale bars, 50 μm.

(C) Accumulation of GM gangliosides in iPSC-derived NSCs. Total amounts of GM1, GM2, and GM3 gangliosides were measured by LC-MS. The bars represent the mean ± SD from three independent experiments. ***$p < 0.001$, disease versus normal, Student's t test.

maturation and/or synaptic function, we next investigated cell survival and neurite outgrowth. Even though the exogenous treatment of GM1 ganglioside was able to increase the amount of GM1 ganglioside in the normal cells (Figure S2B), there was no difference in the number of MAP2-positive surviving cells between control and disease-generated neurons with or without GM1 ganglioside exposure, nor indeed in total neurite length or neurite outgrowth in both control and disease neurons with or without GM1 ganglioside exposure (Figures S2C and S2D). Furthermore, the lactate dehydrogenase (LDH) cytotoxicity assay confirmed that cell death was not enhanced with or without GM1 ganglioside exposure either, comparing with the controls (Figure S2E). These findings support that accumulated GM1 ganglioside plays a negligible role in cell survival and neurite outgrowth during neuronal differentiation. To then examine synaptic function more directly, we monitored an exocytosis process using FM1-43 imaging. In synaptic sites, functional mature neurons release neurotransmitters, which are visualized with fluorescent-labeled FM1-43 dye, and the loss of fluorescent intensity provides an exocytosis index (Numakawa et al., 1999). The fluorescent signal of FM1-43 disappeared in control neurons after application of a high potassium solution (50 mM) to induce cell depolarization, whereas it failed to reduce in the A154 and A360 disease-derived neurons (maintained for 62 days) (left panels in Figures 2C and S2F), suggesting a reduction of exocytotic activity. The disease neurons maintained for longer durations (A138 at day 98) still exhibited diminished exocytotic activity (right panels in Figure 2C).

We next asked whether or not the reduction of exocytotic activity affected neurotransmitter release. Neurotransmitters such as glutamate are mainly released at the synapses with exocytosis. When release is impaired, the amount of the neurotransmitter decreases in the culture containing the neurons after depolarization compared with normal. As expected, the amount of glutamate was lower in the culture containing GM1 gangliosidosis-derived neurons than in that containing control neurons (Figure 2D). Taken together, these results suggested that the reduction of exocytosis impaired neurotransmitter release in GM1 gangliosidosis-derived neurons.

To confirm the results thus far, we took advantage of a GM1 gangliosidosis animal model, the BKO mouse, which carries the null mutation of the *Glb1* gene encoding β-galactosidase and mimics the disease phenotype, including GM1 ganglioside accumulation in neural cells (Matsuda et al., 1997). We isolated cortical neurons from the brain of the model and examined neurotransmitter release using the same method as described here. The primary cultured neurons derived from BKO mice failed to

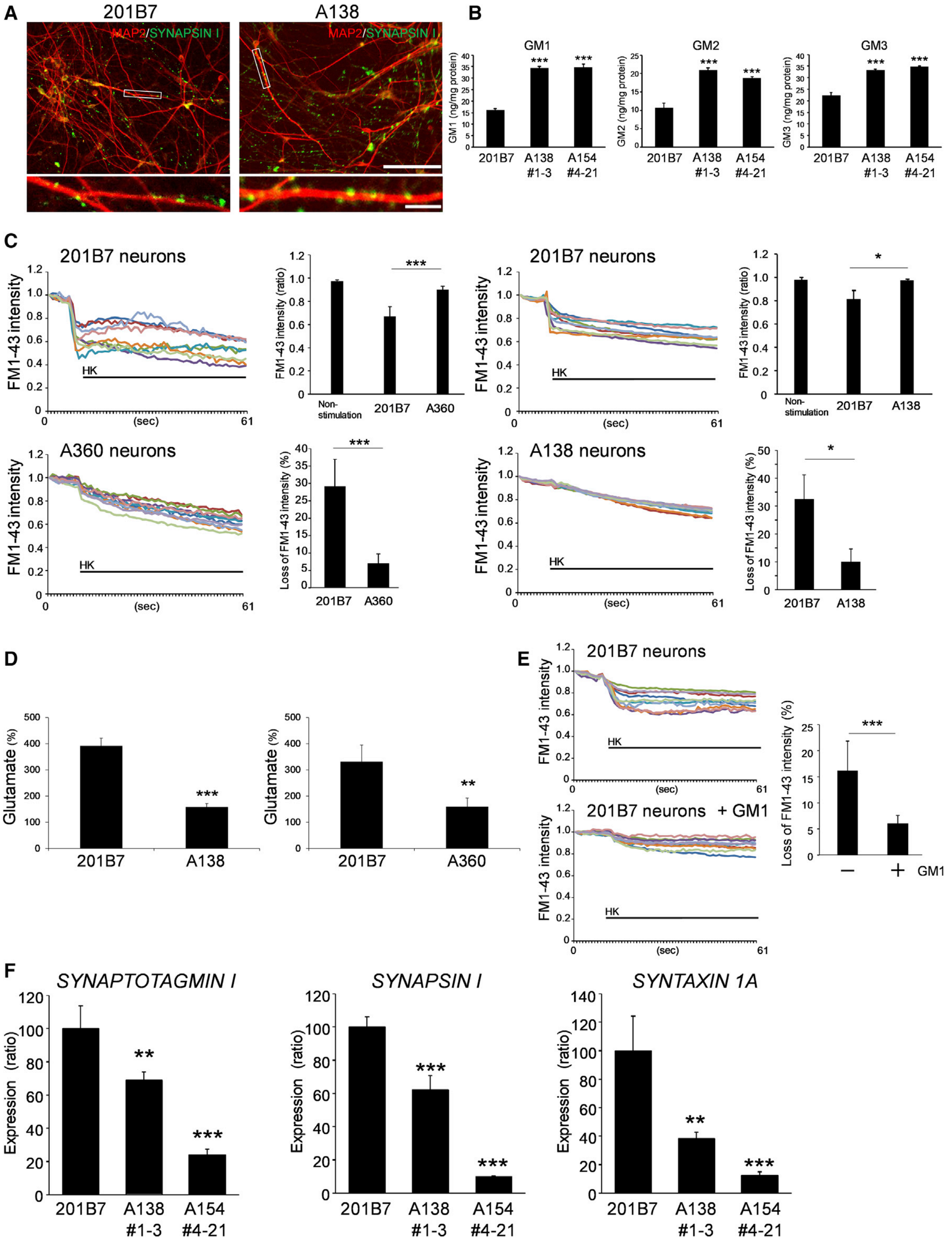

Figure 2. Deficit in Presynaptic Function of GM1 Gangliosidosis Neurons

(A) Day 66 differentiated neurons from control (201B7) and disease (A138 1-3) NSCs were immunostained with anti-MAP2 (red) and SYNAPSIN I (green) antibodies. Boxed areas are magnified below each picture. Scale bars: 100 μm (upper), 20 μm (lower).

(B) Increased accumulation of GM1, 2, and 3 gangliosides in disease neurons. Quantification by LC-MS analysis. The bars represent the mean ± SD from three independent experiments. ***$p < 0.001$, disease versus normal, Student's t test.

(legend continued on next page)

release FM1-43, confirming impaired presynaptic functions such as neurotransmitter release in GM1 gangliosidosis neurons (Figure S2G).

We next investigated whether or not supplying β-GAL enzyme restored the impairment in exocytosis in GM1 gangliosidosis-derived neurons. The exocytosis activity was partially restored with β-GAL treatment in GM1 gangliosidosis-derived neurons, suggesting that β-GAL deficiency affected exocytosis in the neurons (Figure S2H).

Long-term treatment with GM1 ganglioside could significantly reduce the releasing efficiency of FM1-43, even in control neurons (Figure 2E). The treatment was able to increase the amount of GM1 ganglioside in the normal cells (Figure S2B). These results suggest that the accumulated GM1 ganglioside affects neurotransmitter release into the synaptic sites *in vitro*. Consistent with such a presynaptic dysfunction, we observed a significant decrease in mRNA expression of presynapse proteins, including *SYNAPTOTAGMIN I*, *SYNAPSIN I*, and *SYNTAXIN* 1A in both A138- and 154-disease neurons (Figure 2F) (Hicks et al., 1997). Therefore, it is possible that accumulated GM1 during neuronal development results in dysfunctional presynaptic machinery.

High-Content Screening for GM1 Gangliosidosis Drug Candidates

The effect of GM1 ganglioside accumulation on presynaptic function *in vitro* prompted us to seek candidate compounds to reduce this abnormal ganglioside level in disease cells (Figure 3A). To establish the necessary high-content screening (HCS) for such an aim, we took advantage of iPSC-derived NSCs, because they are more easily expandable than post-mitotic differentiated neurons, and precise detection of cytoplasmic AF488-CTB signals representing an accumulation of GM1 ganglioside was easily achieved (i.e., neurons are too thin to meaningfully visualize the AF488-CTB signal). A138 NSCs seeded in 96-well plates were incubated with 2,217 compounds containing already approved drugs and major chemicals used in pathway analyses (provided by Drug Discovery Initiative, University of Tokyo, and purchased from Sigma-Aldrich) for 72 h, and then stained with AF488-CTB for quantification of cellular fluorescence in each well by image analysis (Figure 3A). Using this drug-screening system in combination with the approved drug library revealed 25 small compounds that reduced the accumulation of GM1 ganglioside. Of these, we focused on the two best compounds, amodiaquine and thiethylperazine, to analyze their effects in detail (Figures 3B, 3C, and S3A and Table 1). LC-MS analysis confirmed the effect of these compounds on ganglioside concentration in disease cells and revealed that GM1, GM2, and GM3 gangliosides were all significantly reduced in the A138 NSCs (Figures 3D and S3B).

We next examined whether amodiaquine and thiethylperazine could restore the presynaptic deficit in disease-derived neurons. Short-term treatment (for 24 h before FM1-43 imaging) with thiethylperazine, but not amodiaquine, significantly recovered the presynaptic dysfunction in A138 neurons (Figure 3E). In contrast, long-term treatment with amodiaquine or thiethylperazine throughout the time course of *in vitro* neural differentiation restored the decreased presynaptic activity of A138 neurons (Figure 3F), and of the other iPSC clones, A154 and A360 (Figures S3C and S3D).

The treatments also significantly restored the decrease of glutamate in the culture containing the GM1 gangliosidosis-derived neurons (Figures S3E and S3F). These results suggest that both amodiaquine and thiethylperazine can reduce GM1 ganglioside accumulation and recover the presynaptic dysfunction characteristic of disease-derived neurons.

Amodiaquine and Thiethylperazine Upregulate Lysosomal Glycosphingolipid Degradation Enzymes and Activate Autophagy

GM1 ganglioside is biosynthesized via a series of synthase processes that start with ceramide in the

(C) Decreased exocytotic release in the gangliosidosis-derived neurons. To examine the exocytotic process, the loss of FM1-43 dye fluorescence was monitored. High potassium (HK) solution (50 mM) was applied for cell depolarization. Left: A360 1-3-disease neurons on day 62. Right: A138 1-3-disease neurons on day 98. Each trace indicates a changed intensity of FM1-43 fluorescence from single vesicle-like buttons. The decreased fluorescence intensity of FM dye was determined by comparing the stimulation intensity (2 s after HK+ stimulation) with basal levels (2 s before the stimulation). The quantitative data are shown as the value of the basal/HK+-stimulated ratio (upper), and loss of FM intensity (%): (basal intensity − HK+-stimulated intensity − fading value of fluorescence) × 100 (lower). *$p < 0.05$ ($n = 10$, where n indicates the number of randomly selected buttons).
(D) Decreased release of glutamate from GM1 gangliosidosis-derived neurons. Left: A138 1-3-disease neurons on day 69. Right: A360 1-3-disease neurons on day 65. The data are displayed as the ratio (%) of the HK+-stimulated release/basal release. Basal release (1 min) was collected before the stimulation with HK (high KCl, 50 mM, 1 min). **$p < 0.01$, ***$p < 0.001$ ($n = 4$, where n is the number of wells for each experimental condition).
(E) Presynaptic function in control neurons with or without exposure to exogenous GM1 ganglioside. 200 nM GM1 ganglioside was applied throughout the time course of neural differentiation (85 days). Right: the quantitative data. ***$p < 0.001$ ($n = 10$, mean ± SD).
(F) Decreased mRNA levels of presynapse proteins in disease neurons. qPCR. **$p < 0.01$, ***$p < 0.001$, disease versus normal, Student's t test ($n = 4$ independent samples).

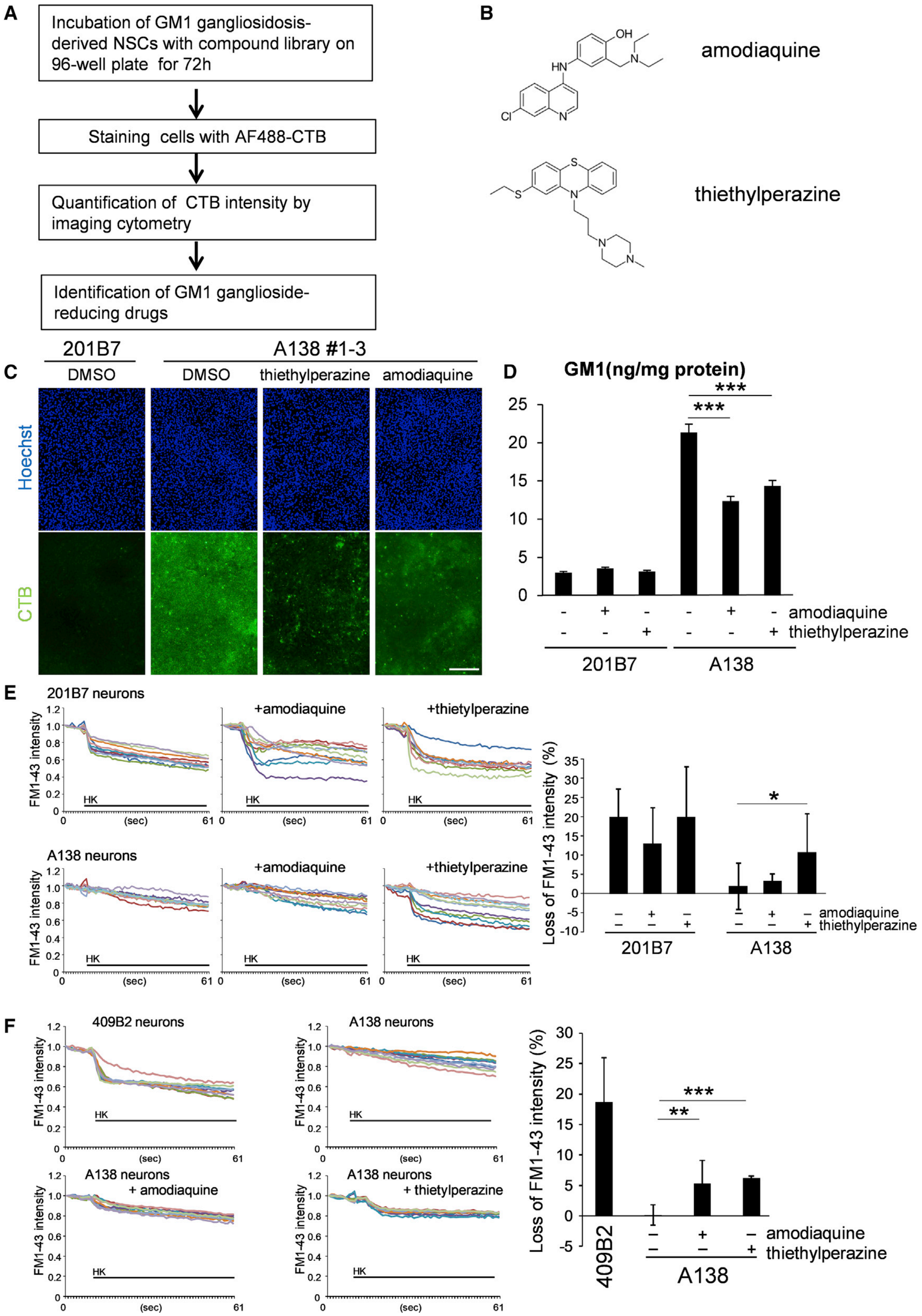

(legend on next page)

endoplasmic reticulum and Golgi apparatus and is subsequently degraded by lysosomal hydrolases (Figure 4A) (Venier and Igdoura, 2012). To clarify possible mechanisms underlying the downregulation of GM1 ganglioside by our two hit compounds, we investigated whether the metabolism of GM1 ganglioside is altered after treatment with amodiaquine or thiethylperazine. After treating disease-derived NSCs with each compound for 72 h, we measured the mRNA levels of a series of enzymes on GM1 ganglioside synthesis and metabolic pathways by qRT-PCR. There was no significant change in expression levels of the biosynthetic synthases (for GM1, GM2, and GM3), *HEXA, GALC*, and *GLB1* (Figure 4B). In contrast, the expression of *NEU1* and *GBA* (which encodes β-GLU) was significantly upregulated in the treated patient-derived NSCs compared with those in vehicle-treated NSCs (Figure 4B). In addition, expression levels of other members of NEU family, *NEU2, NEU3*, and *NEU4*, were not increased in the treated NSCs compared with the non-treated NSCs (Figure S4A). Accordingly, the enzyme activities of NEU1 and β-GLU were also enhanced with amodiaquine and thiethylperazine treatments, while β-GAL activity was unaffected (Figures 4C–4E and S4B). Combined with our finding of reduced GM1, GM2, and GM3 ganglioside accumulation with the two hit compounds (Figures 3D and S3B), the enzyme analyses suggest activation of the lysosomal sphingolipid degradation pathway, and consequently, downregulation of ganglioside accumulations.

Cellular autophagy is impaired in lysosomal storage diseases (Lieberman et al., 2012; Maetzel et al., 2014; Soga et al., 2015), thus here, we investigated the autophagy pathway in NSCs treated with the hit compounds, focusing on autophagy markers such as microtubule-associated protein 1 light chain 3-II (LC3-II) and the insoluble p62/SQSTM1 (p62). The C-terminal processing of LC3 produces LC3-I, which is modified to LC3-II following the initiation of autophagosome formation (Kabeya et al., 2000). Because p62/SQSTM1 (p62) binds to LC3 and is degraded upon fusion with the lysosome, impairment of autophagy flux accumulates insoluble p62 (Komatsu et al., 2007). Thus, we confirmed the upregulation of LC3-II and insoluble p62 in GM1 gangliosidosis-derived skin fibroblasts compared with normal controls and suggested that although autophagy initiation is upregulated in fibroblasts, the autophagic flux is impaired (Figure S4C). Surprisingly, both markers were not enhanced in GM1 gangliosidosis-derived NSCs compared with normal cells (Figures 4F and S4D); however, treatment with amodiaquine and thiethylperazine enhanced the expression of LC3-II and reduced insoluble p62 in both normal and GM1 NSCs (Figures 4F and S4D). Consistently, p62 granules were detected in both NSCs treated with the two compounds, appearing only in those cells with high autophagy activity (Figure S4E) (Ben Younes et al., 2011). To further assess autophagy activity, we took advantage of GFP-LC3-RFP-LC3ΔG, a fluorescent probe for calculating autophagy flux. This probe is cleaved by endogenous ATG4 into equal amounts of GFP-LC3 and RFP-LC3ΔG. While GFP-LC3 is degraded by autophagy, RFP-LC3ΔG remains in the cytosol and acts as an internal control. Thus, autophagic flux can be estimated by calculating the GFP/RFP signal ratio, which is decreased with the activation of autophagy. Treatments with amodiaquine and thiethylperazine significantly reduced the GFP/RFP signal ratio in both normal and patient NSCs (Figures 4G and S4F). The reduction levels were similar to that of the treatment with rapamycin, which is known as an activating drug for autophagy (Figure 4G) (Kaizuka et al., 2016). In contrast, the treatments with amodiaquine and thiethylperazine did not reduce the ratio in HeLa carcinoma cells

Figure 3. *In Vitro* Presynaptic Function Is Improved with Treatments with the Drug Candidates

(A) Flowchart of imaging cytometry-based high-content screening. A138 1-3 NSCs were plated onto 96-well plates and incubated with 5 μM of validated compounds for 72 h. The cells were stained with AF488-CTB, and the fluorescence intensity was measured using an IN Cell Analyzer 6000 (GE Healthcare).

(B) Skeletal formula of two hit compounds, amodiaquine and thiethylperazine.

(C) Representative images of cells stained with AF488-CTB in the hit-compound-treated wells. Scale bars, 100 μm.

(D) Reduction of GM1 ganglioside in control NSCs (201B7) and disease NSCs (A138 1-3) treated with amodiaquine and thiethylperazine. Gangliosides were purified from the NSCs treated with amodiaquine (5 μM) and thiethylperazine (5 μM) and were measured by LC-MS. The bars represent the mean ± SD from three independent experiments. ***$p < 0.001$, treated versus non-treated NSCs, Student's t test.

(E) Effect on the abnormal exocytotic release after short-term (for 24 h before the FM1-43 imaging) treatments with 1 μM amodiaquine or 1 μM thiethylperazine. Each trace shows the signal of FM1-43 intensity from single FM1-43 buttons (n = 10, where n is the number of randomly selected buttons). Right: the quantitative data for loss of FM intensity (%): (basal intensity − HK+-stimulated intensity − fading value of fluorescence) ×100. *$p < 0.05$ (n = 10 where n is the number of randomly selected buttons).

(F) Improvement of the abnormal exocytotic release of A138 1-3 neurons after long-term treatments (61 days) with 1 μM amodiaquine or 1 μM thiethylperazine. The neural cells were treated throughout the time course of neural differentiation. Right: the quantitative data were calculated as described in (E). **$p < 0.01$, ***$p < 0.001$ (n = 10 where n is the number of randomly selected buttons).

Table 1. List of Hit Compounds that Can Reduce the GM1 Ganglioside Accumulation

Compound Name	Comments	GM1 Accumulation (%)
Amodiaquine dihydrochloride dihydrate	Antimalarial, heme polymerase inhibitor, schizonticide	16.83736026
Thiethylperazine malate	Antiemetic, adrenergic antagonist	16.85138567
Sulfamerazine	Antibacterial, inhibitor of folic acid synthesis	17.03440890
Ungerine nitrate	Sedative, enhancer of analgesics	17.36768776
Amiodarone hydrochloride	Antiarrhythmic, Na^+ channel blocker, anti-anginal, K^+ channel blocker, non-competitive beta-adrenergic blocker	21.31707824
Sertindole	Antipsychotic	23.97221777
Delcorine	Antiarrhythmic, nicotinic ligand, hypotensive	24.28350616
Perphenazine	Antipsychotic, dopamine antagonist, tranquilizer	25.89101795
Althiazide	Diuretic, Na^+ Cl^- transport inhibitor, antihypertensive	31.11239360
Diethylstilbestrol	Estrogen, antineoplastic	31.23824793
Acacetin	Antioxidant, inhibitor of topoisomerase I, antitumor agent, inhibitor of glutathione reductase	31.35048887
Harmol hydrochloride monohydrate	Anxiolytic, liver conjugation probe	34.48043792
Skimmianine	Sedative, 5-HT ligand, anticonvulsant	37.60694168
Succinylsulfathiazole	Antibacterial, inhibitor of folic acid synthesis	37.63117478
Fillalbin	Tropane alkaloid	40.66893369
Canavanine sulfate monohydrate (L,+)	Anticancer agent, nitric oxide inductible synthetase inhibitor, cholesterol-lowering agent	43.11514126
Harmaline hydrochloride dihydrate	Antihelminthic, monoamine oxidase inhibitor, antimalarial, vasorelaxant	45.67007687
Miglustat	Inhibitor of glucosylceramide synthase	52.72368500
Trihexyphenidyl-D,L hydrochloride	Antiparkinsonian, anticholinergic	54.37458264
Fluoxetine hydrochloride	Antidepressant, 5-HT uptake inhibitor	57.79212899
Lovastatin	Anti-hypercholesterolemic, HMG CoA reductase inhibitor	60.79971522
Haloperidol	Antipsychotic, dopamine antagonist, neuroleptic, alpha antagonist, antiemetic	65.56089756
Prenylamine lactate	Vasodilator, calcium channel blocker	68.01129453
Bromperidol	Antipsychotic, dopamine antagonist	68.94978044
Convolamine hydrochloride	Vasodilatator, cholinergic ligand, local anesthetic, hypotensive	70.32706820

Hit compounds found in Figure 3A are listed (selected compounds that reduced GM1 ganglioside by more than 20%). GM1 accumulation (%) is calculated as the percentage of $(A_T - N_U)/(A_U - N_U)$. A_T, CTB fluorescence intensity of compound-treated A138 NSCs; A_U, CTB fluorescence intensity of untreated A138 NSCs; N_U, CTB fluorescence intensity of untreated 201B7 NSCs.

(Figures 4G and S4F). The results suggest that the two compounds have potential to activate autophagy, and their effects on autophagy were dependent on the cell type.

Taken together, these results indicate that the two hit compounds can reduce GM1 ganglioside accumulation and upregulate both autophagy and enzyme activities involved in lysosomal sphingolipid degradation.

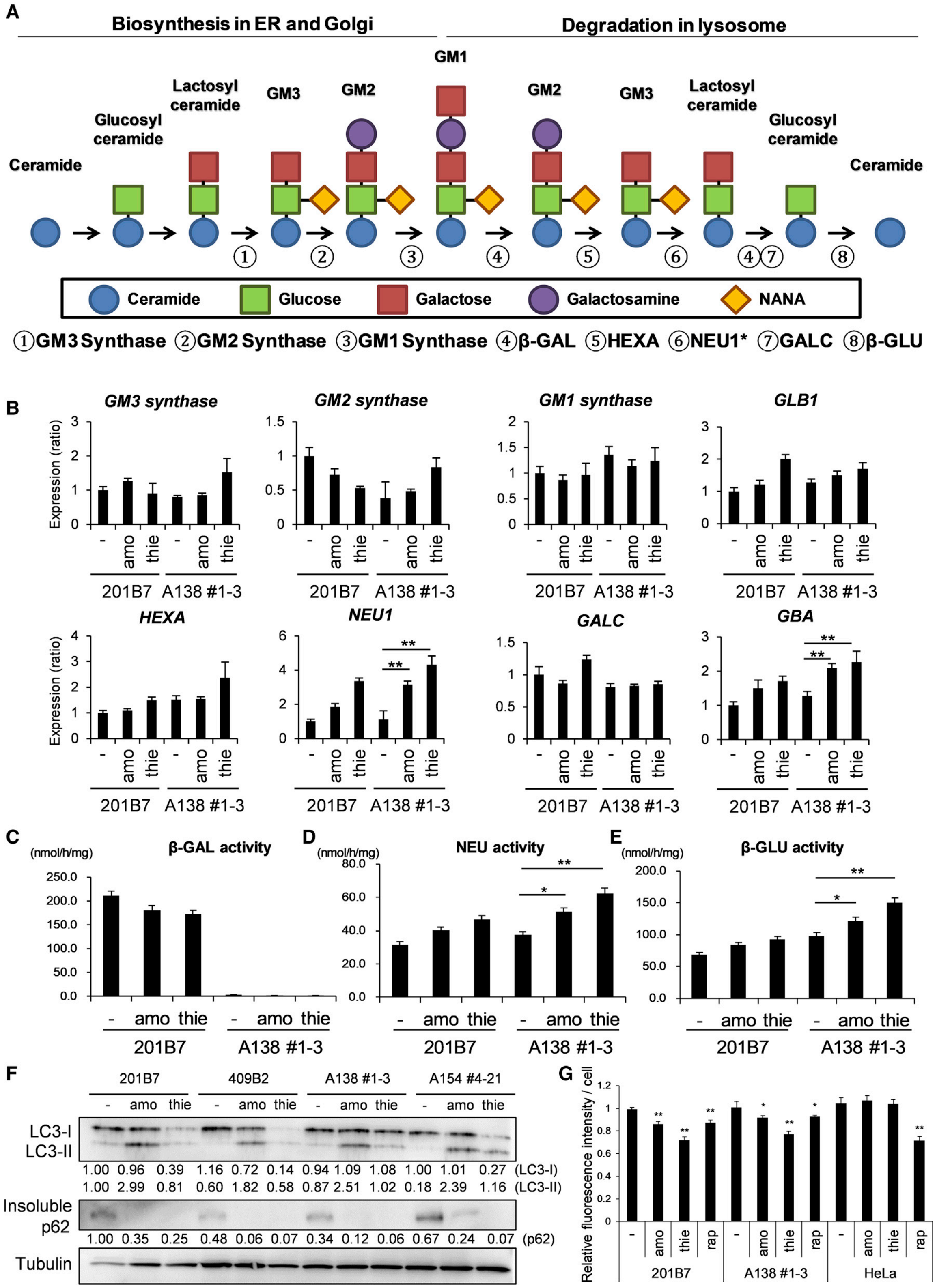

(legend on next page)

Hit Compounds Suppress GM1 Ganglioside Accumulation *In Vivo*

Finally, we examined whether the hit compounds identified were effective *in vivo*. To verify the preventive effect of hit compounds against an accumulation of GM1 ganglioside in the brain, we treated BKO mice with amodiaquine (40 mg/kg, i.p.) or thiethylperazine (6 mg/kg, i.p.) twice a day from postnatal day 9 (P9) to 15. Then, mice brains were sampled and analyzed to quantify levels of GM1 ganglioside; AF488-CTB staining revealed that both amodiaquine and thiethylperazine treatments could reduce the signal of GM1 ganglioside in the BKO mice compared with the controls (Figures 5A and S5A). Quantification of GM1 ganglioside content by LC-MS showed a significant reduction of GM1 ganglioside accumulation in the brain of treated mice (Figure 5B). Similar to the results *in vitro*, the treatments also activated autophagy and enhanced β-GLU and NEU1 but not β-GAL activities (Figures 5C–5E and S5B). These results indicate that treatment with amodiaquine and thiethylperazine affects GM1 accumulation in the brain of model mice.

DISCUSSION

Here, we generated iPSCs from the cells of GM1 gangliosidosis patients and demonstrated that these lines provide a cellular model of pathological neural dysfunction. Using iPSCs, we found drug candidates for future therapy that reduced GM1 ganglioside accumulation *in vitro* and *in vivo*. Specifically, the candidate compounds activated both the metabolic pathway from GM3 ganglioside to ceramide and the autophagic response.

Considering that neural cells are predominantly affected in GM1 gangliosidosis (Sandhoff and Harzer, 2013), we directed the iPSCs to differentiate into both NSCs and mature neurons. Using this system, we confirmed excessive GM1 ganglioside accumulation in both cell types and low β-GAL activity compared with normal controls. While the pathogenesis of GM1 gangliosidosis most likely involves a massive and progressive increase of GM1 ganglioside in neural cells, a more precise understanding of the downstream effects of such cellular events has yet to be elucidated. Some studies implicated the accumulation of GM1 ganglioside in causing neuronal apoptosis via the UPR and endoplasmic reticulum calcium signaling in a mouse model (Sano et al., 2009; Tessitore et al., 2004); however, we did not detect cell death in the neuronal differentiation of GM1 gangliosidosis iPSCs. These discrepancies could be in part due to differences in the species studied (mouse versus human), cell types (MEF, neurosphere versus neuron), and β-GAL gene mutations (null versus point mutation). On the other hand, our analysis revealed impaired neurotransmitter release from presynaptic sites in mature neurons derived from GM1 gangliosidosis iPSCs and the mouse model. The GM1 gangliosidosis-derived mature neurons exhibited a similar number of SYNAPSIN I-positive puncta to control neurons. The neurons expressed less of the essential synapse-associated molecules than controls, including *SYNAPTOTAMINE I*, *SYNAPSIN I*, and *SYNTAXIN* 1A (Hicks et al., 1997). It has been well accepted that synaptic vesicles containing snare proteins, such as

Figure 4. Amodiaquine and Thiethylperazine Enhance Lysosomal Glycosphingolipid Degradation and Activate Autophagy

(A) Schemes for the synthetic and catabolic pathways of glycosphingolipid. GM1, GM2, and GM3 gangliosides are synthesized from ceramide by the sequential addition of monosaccharide or N-acetylneuraminic acid (NANA). Ganglioside catabolism occurs in reverse order from their anabolism, with the stepwise actions of lysosomal glycosidase specific for gangliosides. Number 1–8 indicate the enzymes of each reaction. *It is controversial that Neu1 is involved in the degradation of GM3 ganglioside (Yoshino et al., 1990; O'Leary and Igdoura, 2012).

(B) Expression of *NEU1* and *β-GLU* is elevated with the candidate treatments. Normal (201B7) and disease-derived (A138 1-3) NSCs were treated with 5 μM amodiaquine and 5 μM thiethylperazine for 72 h qPCR. Data were normalized to the levels in 201B7-NSCs (−) (mean ± SD from three independent experiments). $^{**}p < 0.01$, treated versus non-treated NSCs, Student's t test. amo, amodiaquine; thie, thiethylperazine.

(C–E) Enzyme activities of NEU1 and β-GLU are also enhanced with the candidate treatments. NSCs were treated with 5 μM amodiaquine (amo) and 5 μM thiethylperazine (thie) for 48 h and enzyme activities of β-GAL (C), NEU1 (D), and β-GLU (E) were measured. Enzyme activities are expressed as nmol/h/mg protein (mean ± SD from three independent experiments). $^{*}p < 0.05$, $^{**}p < 0.01$, treated versus non-treated NSCs, Student's t test.

(F) Autophagic flux was upregulated in NSCs treated with amodiaquine and thiethylperazine. The protein levels of LC3-II were increased and those of insoluble p62 decreased in NSCs treated with both compounds. NSCs were treated with 5 μM amodiaquine (amo) and 5 μM thiethylperazine (thie) for 72 h, western blotting. The band intensities were measured using ImageJ software, normalized to the non-treated 201B7 and described below the images.

(G) Measurement of autophagic flux using a GFP-LC3-RFP-LC3ΔG probe. NSCs stably expressing GFP-LC3-RFP-LC3ΔG were treated with the candidates and 1 μM rapamycin (rap) as described in (F). The fluorescence intensity was measured using an IN Cell Analyzer 6000 and the fluorescence intensity ratio (GFP/RFP) of the samples was normalized to that of non-treated 201B7. $^{*}p < 0.05$, $^{**}p < 0.01$ (n = 5 independent samples).

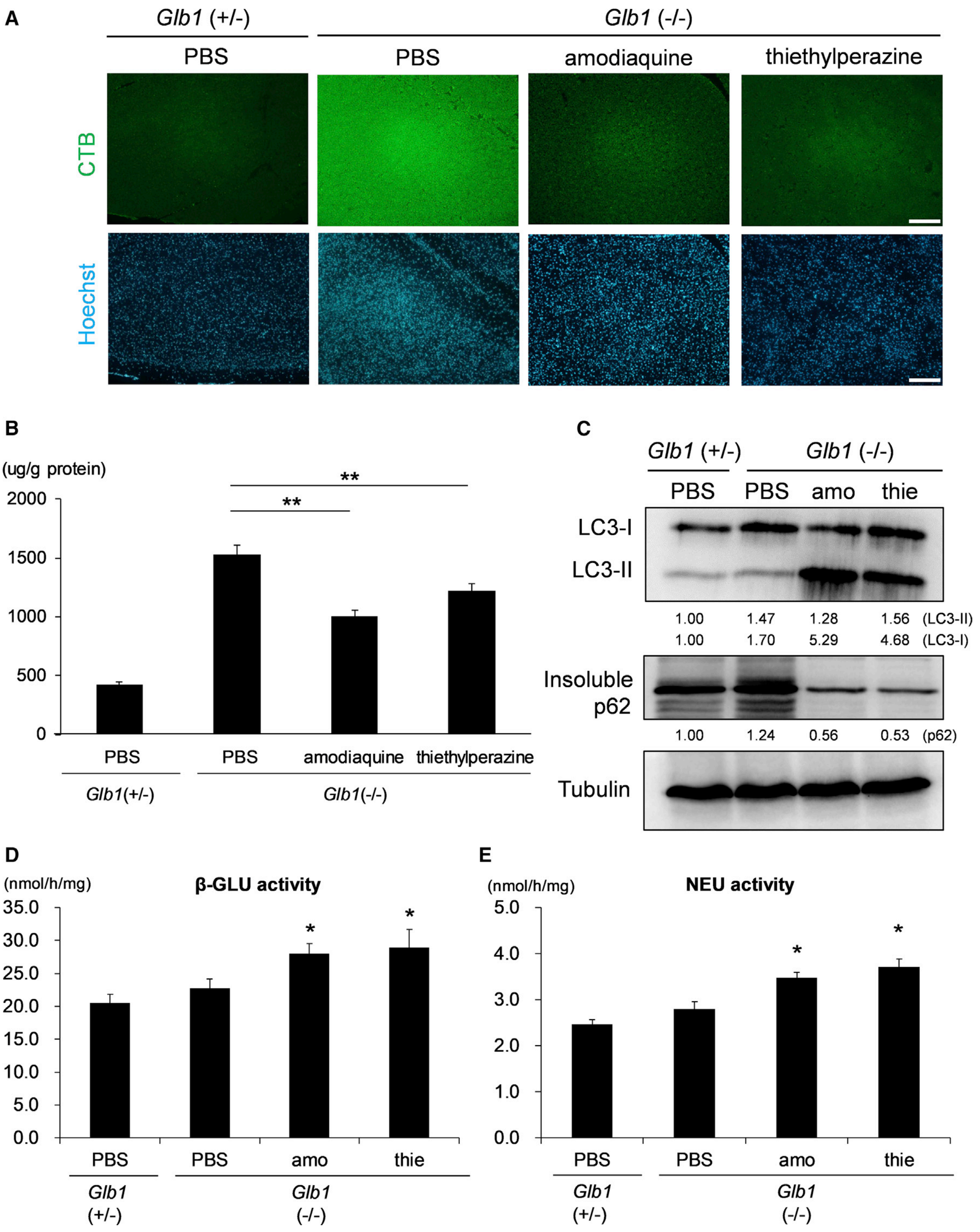

Figure 5. Treatments with Amodiaquine and Thiethylperazine Can Reduce GM1 Ganglioside Accumulations in the Brain of Model Mice

(A) β-GAL KO (BKO, *Glb1* −/−) mice were treated with amodiaquine (40 mg/kg, i.p.) and thiethylperazine (6 mg/kg, i.p.) twice a day from P9 to P15. Brain slices were stained with AF488-CTB and Hoechst to visualize GM1 ganglioside accumulation. Scale bars, 200 μm.

(B) The treatments with two candidates reduced GM1 ganglioside accumulation in β-GAL KO mice, which were treated as described in (A). GM1 ganglioside was extracted from brain homogenates and measured by LC-MS.

(legend continued on next page)

SYNTAXIN 1A, and other presynaptic proteins, including SYNAPTOTAMINE I and SYNAPSIN I, have a pivotal role in exocytotic release of neurotransmitters in neurons (Hilfiker et al., 1999; Li and Chin, 2003; Tucker and Chapman, 2002; Vardar et al., 2016). Therefore, our results suggest that the disease-associated defect in neurotransmitter release is due to their reduced expression at individual presynaptic sites. We also found that long-term treatment with GM1 ganglioside during neural differentiation affected neurotransmitter release from the presynapse even in normal iPSC-derived neurons. This study thus supports a pathogenic mechanism underlying neural dysfunction in GM1 gangliosidosis, in which the excess accumulation of GM1 ganglioside inhibits synaptic function via the impairment of synapse-associated molecules during neuronal differentiation.

Considering that GM1 ganglioside itself could cause synaptic dysfunction, removing the accumulated GM1 ganglioside should be effective in treating the disease. To this end, we sought to identify drug candidates that could suppress the accumulation of GM1 ganglioside using HCS with iPSC-derived NSCs. Using a chemical library containing 2,217 compounds, we found 25 compounds that reduced GM1 ganglioside accumulation in NSCs. Among them, amodiaquine and thiethylperazine also reduced GM1 ganglioside accumulation in the model mice. Accumulated GM1 ganglioside was observed in the brain of BKO mice as early as when they were born. With regard to the clinical symptoms, they were born normal and healthy at least until 4 months of age. Therefore, we investigated the effects of two compounds on the accumulation in 15-day aged BKO mice. The previous study also reported that the mice developed definite neurological symptoms from 6 months of age and they died at 7–10 months of age (Matsuda et al., 1997). Although this study did not approach the neurological symptoms and the survival rate in the compounds-treated mice, further study is required to find out whether the compounds are still effective on the neurological symptoms and the survival rate *in vivo*. Amodiaquine and thiethylperazine also restored the defective neurotransmitter release at the synapse of patient-derived neurons. While it remains unclear whether reducing GM1 ganglioside can recover neuronal function in clinical GM1 gangliosidosis, our results provide the demonstration that suppressing the accumulation of GM1 ganglioside might alleviate the neuronal dysfunction associated with this disease.

Based on this study, we propose two mechanisms underlying the effect of amodiaquine and thiethylperazine on GM1 ganglioside accumulation. First, our results suggest a strategy for GM1 gangliosidosis therapy whereby activation of the metabolic pathway from GM3 ganglioside to ceramide might affect GM1 ganglioside accumulation. Although the treatment with amodiaquine and thiethylperazine reduced GM1 ganglioside accumulation in the patient cells, we did not detect any upregulation of β-GAL activity. In addition, the treatment did not affect the expression of GM gangliosides synthases; however, we did demonstrate enhanced enzymatic activities, including NEU1 and β-GLU in GM1 ganglioside degradation. NEU1 encodes the lysosomal neuraminidase enzyme, sialidase, which removes the terminal sialic acid from oligosaccharide chains and metabolizes GM3 ganglioside to lactosylceramide by removing neuraminic acid (Venier and Igdoura, 2012). In the lysosome, this enzyme is also part of a heterotrimeric complex together with β-GAL and cathepsin A (Bonten et al., 2014), and upregulation of NEU1 expression with the treatment might affect formation of the heterotrimeric complex and somehow stabilize the β-GAL component. β-GLU is an enzyme with glucosylceramidase activity that converts glucosylceramide to ceramide in lysosomes (Venier and Igdoura, 2012). Mutations in the β-GLU gene cause Gaucher's disease, which is characterized by neuronal disturbance and could also be associated with Parkinson's disease (Suzuki et al., 2015). With respect to GM1 ganglioside accumulation, we showed that GM2 and GM3 gangliosides accumulated in GM1 gangliosidosis-derived NSCs and neurons, suggesting that synthetic and/or degradative pathways for GM1 ganglioside are stagnated in GM1 gangliosidosis. A previous study showed accumulations of GM2 and GM3 gangliosides in patients' brain (Hahn et al., 1997). Reactivating the pathways from GM3 ganglioside to ceramide could remove this stasis and progress to relaxation of GM1 ganglioside accumulation. GM2 and GM3 gangliosides are converted from GM1 ganglioside (i.e., GM2 and GM3 gangliosides are downstream products of GM1 ganglioside). At the same time, GM2 and GM3 gangliosides are also substrates for GM1 ganglioside (i.e., GM2 and GM3 gangliosides are

(C) The treatments with our two candidates activated autophagy in β-GAL KO mice, which were treated as described in (A). The brain homogenates were prepared and subjected to western blot analysis with anti-LC3B, anti-tubulin, or anti-p62 antibodies. The band intensities were measured using ImageJ software, normalized to the PBS-treated *Glb1* (+/−) and described below the images.
(D and E) Enzyme activities of NEU and β-GLU were also enhanced with the candidate treatments in β-GAL KO mice, which were treated as described in (A). The brain homogenates were prepared and the enzyme activities of β-GLU (D), and NEU (E) were measured. *$p < 0.05$, each candidate-treated *Glb1* (−/−) versus PBS-treated *Glb1* (−/−), Student's t test (n = 3 independent samples).

also located upstream in the biosynthesis of GM1 ganglioside). Activating the metabolic pathway of GM2 and GM3 ganglioside might affect the conversion into GM1 from precursor gangliosides (GM2 and GM3) in the biosynthesis pathway of GM1 ganglioside.

We also identified autophagy activation as a possible second mechanism by which our drug agents exert their demonstrated effects on neural cells, and cellular autophagy is impaired in lysosomal storage diseases (Lieberman et al., 2012; Maetzel et al., 2014; Soga et al., 2015). We confirmed the upregulations of LC3-II and insoluble p62 in GM1 gangliosidosis-derived skin fibroblasts compared with normal controls, suggesting that autophagy initiation is upregulated in the fibroblasts, in turn impairing autophagic flux; however, both markers were not at all enhanced in GM1 gangliosidosis-derived NSCs. This discrepancy might be due to the rapid turnover of autophagic flux in neural cells compared with non-neural cells (Yue et al., 2009). We found that the tested compounds could enhance the protein levels of LC3-II and downregulate the insoluble p62 expression both in NSCs and in mouse models. Consistently, the treatments also upregulated p62 granules in GM1 gangliosidosis-derived NSCs, and these granules appear in cells with high autophagy activity such as during starvation (Ben Younes et al., 2011). A previous study using iPSCs derived from Niemann-Pick type C disease, a lysosomal storage disorder characterized by excess deposition of cellular cholesterol, showed that autophagy-inducing compounds could have cytoprotective effects even without affecting the deposition of cholesterol itself and that inducing autophagy might be a promising treatment option for lysosomal storage diseases (Maetzel et al., 2014). The present study suggests that such a scenario might also be applied for GM1 gangliosidosis, although our case did not show any cytoprotective effect on the neurons derived from GM1 iPSCs, although activating autophagy could still have a potential effect on GM1 gangliosidosis. We showed that amodiaquine and thiethylperazine reduced GM1 ganglioside accumulation with activating autophagy. Because these compounds activated autophagy in both patient cells and normal cells, they must affect the autophagic pathway directly, i.e., their autophagy-inducing effects were not due to the outcome of reducing GM1 ganglioside accumulation. Autophagy plays a role in the digestion of waste products in cells. Its activation promotes the metabolism of intracellular components. Therefore, the metabolism of GM gangliosides contained in major membrane components of cells may also be activated with activation of autophagy.

In contrast to our results, a previous study demonstrated that amodiaquine treatment inhibited autophagy function in a melanoma cell line (Qiao et al., 2013). In this study, the treatment did not activate autophagy flux in HeLa carcinoma cells. This suggested that the effects on autophagy with amodiaquine and thiethylperazine treatments were dependent on the cell type.

This study highlights the downstream pathway of GM1 ganglioside degradation and autophagy as a potential therapeutic target and presents drug candidates, including amodiaquine and thiethylperazine, for treating GM1 ganglioside accumulation in patients with GM1 gangliosidosis.

EXPERIMENTAL PROCEDURES

Imaging Cytometry-Based High-Content Screening for GM1-Reducing Drugs

A138 and 201B7 NSCs were plated at 5×10^4 cells/well in 96-well plates coated with Geltrex, and then compound libraries (1,457 compounds were kindly provided from Drug Discovery Initiative, The University of Tokyo, https://www.ddi.u-tokyo.ac.jp/en/, and 760 compounds were purchased as "Petit screening 2014" from Sigma-Aldrich) were added to each well at 5 μM. After incubation for 72 h, the cells were fixed for 10 min in 4% paraformaldehyde in PBS (pH 7.4) at room temperature, permeabilized with 0.1% Triton X-100 in PBS, and then blocked with 1% BSA in PBS for 10 min at room temperature. GM1 gangliosides in the cells were stained with Alexa Fluor 488-conjugated cholera toxin B (CTB) in PBS, and the fluorescence intensities of CTB staining were measured with an IN Cell Analyzer 6000 using the Developer Toolbox software (GE Healthcare). The CTB fluorescence intensity of untreated 201B7 NSCs was used for negative controls. All the procedures from plating to staining were automated using the Biomek NX laboratory automation workstation (Beckman Coulter). Statistical evaluation of the results yielded a reproducible Z′-factor value range of 0.78–0.81, demonstrating the excellent assay. Z′ factor $= 1 - (3 \times SD_2 + 3 \times SD_1)/(X_2 - X_1)$ where SD_1, X_1 and SD_2, X_2 represent the standard deviations and means of the minimal (1) and maximal (2) signals, respectively (Mayr and Bojanic, 2009).

Statistics

Unless indicated in the figure legends, an unpaired, 2-tailed Student's t test was used to calculate p values and evaluate the statistical significance of the difference between the indicated samples. The differences were considered to be significant at a 5% level.

Study Approval

Human samples were collected and analyzed in accordance with approval from the Ethics Committee for Gene Analysis and Genome Research of Kumamoto University. Subjects gave informed consent. All animal experiments conformed to the animal use guidelines of the Committee for Ethics on Animal Experiments of Kumamoto University.

Other Materials and Methods

Other materials and methods are described in Supplemental Experimental Procedures.

SUPPLEMENTAL INFORMATION

Supplemental Information can be found online at https://doi.org/10.1016/j.stemcr.2020.03.012.

AUTHOR CONTRIBUTIONS

R.K., T.N., and T.E. designed the experiments. R.K., T.N., H.O., Y.Y., T.O., and S.I. performed the experiments and analyzed the data. N.F. and H.F. provided the Sendai viruses and patient samples, respectively. R.K., T.N., and T.E. wrote the manuscript. All authors edited and approved the manuscript.

ACKNOWLEDGMENTS

The authors thank Yumi Soejima, Toyoko Tsuru, Fanyan Wei, and Dr. Shirou Matsumoto for their technical support and helpful discussions. BKO mice were kindly provided by Dr. Junichiro Matsuda at National Institute of Health and Dr. Eiji Nanba at Tottori University in Japan. This study was supported, in part, by grants from the Ministry of Health, Labour and Welfare of Japan, the Japan Agency for Medical Research and Development (A-MED), and Japan Society for the Promotion of Science KAKENHI grant number 16K19654. The compound library was provided by Drug Discovery Initiative, University of Tokyo.

Received: November 6, 2019
Revised: March 13, 2020
Accepted: March 17, 2020
Published: April 16, 2020

REFERENCES

Baek, R.C., Broekman, M.L.D., Leroy, S.G., Tierney, L.A., Sandberg, M.A., d'Azzo, A., Seyfried, T.N., and Sena-Esteves, M. (2010). AAV-mediated gene delivery in adult GM1-gangliosidosis mice corrects lysosomal storage in CNS and improves survival. PLoS One *5*, 16.

Ben Younes, A., Tajeddine, N., Tailler, M., Malik, S.A., Shen, S., Metivier, D., Kepp, O., Vitale, I., Maiuri, M.C., and Kroemer, G. (2011). A fluorescence-microscopic and cytofluorometric system for monitoring the turnover of the autophagic substrate p62/SQSTM1. Autophagy *7*, 883–891.

Bonten, E.J., Annunziata, I., and d'Azzo, A. (2014). Lysosomal multienzyme complex: pros and cons of working together. Cell. Mol. Life Sci. *71*, 2017–2032.

Brunetti-Pierri, N., and Scaglia, F. (2008). GM(1) gangliosidosis: review of clinical, molecular, and therapeutic aspects. Mol. Genet. Metab. *94*, 391–396.

Elliot-Smith, E., Speak, A.O., Lloyd-Evans, E., Smith, D.A., van der Spoel, A.C., Jeyakumar, M., Butters, T.D., Dwek, R.A., d'Azzo, A., and Platt, F.M. (2008). Beneficial effects of substrate reduction therapy in a mouse model of GM1 gangliosidosis. Mol. Genet. Metab. *94*, 204–211.

Hahn, C.N., del Pilar Martin, M., Schröder, M., Vanier, M.T., Suzuki, K., Hara, Y., Suzuki, K., and d'Azzo, A. (1997). Generalized CNS disease and massive GM1-ganglioside accumulation in mice defective in lysosomal acid β-galactosidase. Hum. Mol. Genet. *6*, 205–211.

Hicks, A., Davis, S., Rodger, J., HelmeGuizon, A., Laroche, S., and Mallet, J. (1997). Synapsin I and syntaxin 1B: key elements in the control of neurotransmitter release are regulated by neuronal activation and long-term potentiation in vivo. Neuroscience *79*, 329–340.

Hilfiker, S., Pieribone, V.A., Czernik, A.J., Kao, H.-T., Augustine, G.J., and Greengard, P. (1999). Synapsins as regulators of neurotransmitter release. Philos. Trans. R. Soc. Lond. Ser. B Biol. Sci. *354*, 269–279.

Iwamasa, T., Ohshita, T., Nashiro, K., and Iwanaga, M. (1987). Demonstration of GM1-ganglioside in nervous-system in generalized GM1-gangliosidosis using cholera TOXIN-B subunit. Acta Neuropathol. *73*, 357–360.

Kabeya, Y., Mizushima, N., Uero, T., Yamamoto, A., Kirisako, T., Noda, T., Kominami, E., Ohsumi, Y., and Yoshimori, T. (2000). LC3, a mammalian homologue of yeast Apg8p, is localized in autophagosome membranes after processing. EMBO J. *19*, 5720–5728.

Kaizuka, T., Morishita, H., Hama, Y., Tsukamoto, S., Matsui, T., Toyota, Y., Kodama, A., Ishihara, T., Mizushima, T., and Mizushima, N. (2016). An autophagic flux probe that releases an internal control. Mol. Cell *64*, 835–849.

Komatsu, M., Waguri, S., Koike, M., Sou, Y., Ueno, T., Hara, T., Mizushima, N., Iwata, J., Ezaki, J., Murata, S., et al. (2007). Homeostatic levels of p62 control cytoplasmic inclusion body formation in autophagy-deficient mice. Cell *131*, 1149–1163.

Latour, Y.L., Yoon, R., Thomas, S.E., Grant, C., Li, C., Sena-Esteves, M., Allende, M.L., Proia, R.L., and Tifft, C.J. (2019). Human GLB1 knockout cerebral organoids: a model system for testing AAV9-mediated GLB1 gene therapy for reducing GM1 ganglioside storage in GM1 gangliosidosis. Mol. Genet. Metab. Rep. *21*, 100513.

Li, L., and Chin, L.-S. (2003). The molecular machinery of synaptic vesicle exocytosis. Cell. Mol. Life Sci. *60*, 942–960.

Lieberman, A.P., Puertollano, R., Raben, N., Slaugenhaupt, S., Walkley, S.U., and Ballabio, A. (2012). Autophagy in lysosomal storage disorders. Autophagy *8*, 719–730.

Maetzel, D., Sarkar, S., Wang, H.Y., Abi-Mosleh, L., Xu, P., Cheng, A.W., Gao, Q., Mitalipova, M., and Jaenisch, R. (2014). Genetic and chemical correction of cholesterol accumulation and impaired autophagy in hepatic and neural cells derived from Niemann-Pick type C patient-specific iPS cells. Stem Cell Reports *2*, 866–880.

Matsuda, J., Suzuki, O., Oshima, A., Ogura, A., Noguchi, Y., Yamamoto, Y., Asano, T., Takimoto, K., Sukegawa, K., Suzuki, Y., et al. (1997). beta-galactosidase-deficient mouse as an animal model for G(M1)-gangliosidosis. Glycoconj. J. *14*, 729–736.

Mayr, L.M., and Bojanic, D. (2009). Novel trends in high-throughput screening. Curr. Opin. Pharmacol. *9*, 580–588.

Morita, M., Saito, S., Ikeda, K., Ohno, K., Sugawara, K., Suzuki, T., Togawa, T., and Sakuraba, H. (2009). Structural bases of GM1 gangliosidosis and Morquio B disease. J. Hum. Genet. *54*, 510–515.

Numakawa, T., Takei, N., Yamagishi, S., Sakai, N., and Hatanaka, H. (1999). Neurotrophin-elicited short-term glutamate release from cultured cerebellar granule neurons. Brain Res. *842*, 431–438.

O'Leary, E.M., and Igdoura, S.A. (2012). The therapeutic potential of pharmacological chaperones and proteosomal inhibitors,

Celastrol and MG132 in the treatment of sialidosis. Mol. Genet. Metab. *107*, 173–185.

Okita, K., Matsumura, Y., Sato, Y., Okada, A., Morizane, A., Okamoto, S., Hong, H., Nakagawa, M., Tanabe, K., Tezuka, K., et al. (2011). A more efficient method to generate integration-free human iPS cells. Nat. Methods *8*, 409–U452.

Qiao, S., Tao, S., Rojo de la Vega, M., Park, S.L., Vonderfecht, A.A., Jacobs, S.L., Zhang, D.D., and Wondrak, G.T. (2013). The antimalarial amodiaquine causes autophagic-lysosomal and proliferative blockade sensitizing human melanoma cells to starvation-and chemotherapy-induced cell death. Autophagy *9*, 2087–2102.

Sandhoff, K., and Harzer, K. (2013). Gangliosides and gangliosidoses: principles of molecular and metabolic pathogenesis. J. Neurosci. *33*, 10195–10208.

Sano, R., Annunziata, I., Patterson, A., Moshiach, S., Gomero, E., Opferman, J., Forte, M., and d'Azzo, A. (2009). GM1-ganglioside accumulation at the mitochondria-associated ER membranes links ER stress to Ca^{2+}-dependent mitochondrial apoptosis. Mol. Cell *36*, 500–511.

Shield, J.P.H., Stone, J., and Steward, C.G. (2005). Bone marrow transplantation correcting beta-galactosidase activity does not influence neurological outcome in juvenile GM1-gangliosidosis. J. Inherit. Metab. Dis. *28*, 797–798.

Soga, M., Ishitsuka, Y., Hamasaki, M., Yoneda, K., Furuya, H., Matsuo, M., Ihn, H., Fusaki, N., Nakamura, K., Nakagata, N., et al. (2015). HPGCD outperforms HPBCD as a potential treatment for Niemann-Pick disease type C during disease modeling with iPS cells. Stem Cells *33*, 1075–1088.

Son, M.Y., Kwak, J.E., Seol, B., Lee, D.Y., Jeon, H., and Cho, Y.S. (2015). A novel human model of the neurodegenerative disease GM1 gangliosidosis using induced pluripotent stem cells demonstrates inflammasome activation. J. Pathol. *237*, 98–110.

Suzuki, Y., Ichinomiya, S., Kurosawa, M., Matsuda, J., Ogawa, S., Iida, M., Kubo, T., Tabe, M., Itoh, M., Higaki, K., et al. (2012). Therapeutic chaperone effect of N-octyl 4-epi-beta-valienamine on murine G(M1)-gangliosidosis. Mol. Genet. Metab. *106*, 92–98.

Suzuki, M., Fujikake, N., Takeuchi, T., Kohyama-Koganeya, A., Nakajima, K., Hirabayashi, Y., Wada, K., and Nagai, Y. (2015). Glucocerebrosidase deficiency accelerates the accumulation of proteinase K-resistant alpha-synuclein and aggravates neurodegeneration in a *Drosophila* model of Parkinson's disease. Hum. Mol. Genet. *24*, 6675–6686.

Takahashi, K., Tanabe, K., Ohnuki, M., Narita, M., Ichisaka, T., Tomoda, K., and Yamanaka, S. (2007). Induction of pluripotent stem cells from adult human fibroblasts by defined factors. Cell *131*, 861–872.

Takamura, A., Higaki, K., Kajimaki, K., Otsuka, S., Ninomiya, H., Matsuda, J., Ohno, K., Suzuki, Y., and Nanba, E. (2008). Enhanced autophagy and mitochondrial aberrations in murine G(M1)-gangliosidosis. Biochem. Biophys. Res. Commun. *367*, 616–622.

Tessitore, A., del P Martin, M., Sano, R., Ma, Y.J., Mann, L., Ingrassia, A., Laywell, E.D., Steindler, D.A., Hendershot, L.M., and d'Azzo, A. (2004). G(M1)-ganglioside-mediated activation of the unfolded protein response causes neuronal death in a neurodegenerative gangliosidosis. Mol. Cell *15*, 753–766.

Tucker, W.C., and Chapman, E.R. (2002). Role of synaptotagmin in Ca^{2+}-triggered exocytosis. Biochem. J. *366*, 1–13.

Vardar, G., Chang, S., Arancillo, M., Wu, Y.-J., Trimbuch, T., and Rosenmund, C. (2016). Distinct functions of syntaxin-1 in neuronal maintenance, synaptic vesicle docking, and fusion in mouse neurons. J. Neurosci. *36*, 7911–7924.

Venier, R.E., and Igdoura, S.A. (2012). Miglustat as a therapeutic agent: prospects and caveats. J. Med. Genet. *49*, 591–597.

Yoshino, H., Miyashita, K., Miyatani, N., Ariga, T., Hashimoto, Y., Tsuji, S., Oyanagi, K., Ohama, E., Ikuta, F., and Suzuki, A. (1990). Abnormal glycosphingolipid metabolism in the nervous system of galactosialidosis. J. Neurol. Sci. *97*, 53–65.

Yue, Z.Y., Friedman, L., Komatsu, M., and Tanaka, K. (2009). The cellular pathways of neuronal autophagy and their implication in neurodegenerative diseases. Biochim. Biophys. Acta *1793*, 1496–1507.

Stem Cell Reports, Volume *14*

Supplemental Information

Novel Drug Candidates Improve Ganglioside Accumulation and Neural Dysfunction in GM1 Gangliosidosis Models with Autophagy Activation

Ryutaro Kajihara, Tadahiro Numakawa, Haruki Odaka, Yuji Yaginuma, Noemi Fusaki, Toshika Okumiya, Hirokazu Furuya, Seiji Inui, and Takumi Era

kajihara *et al* **Figure S1**

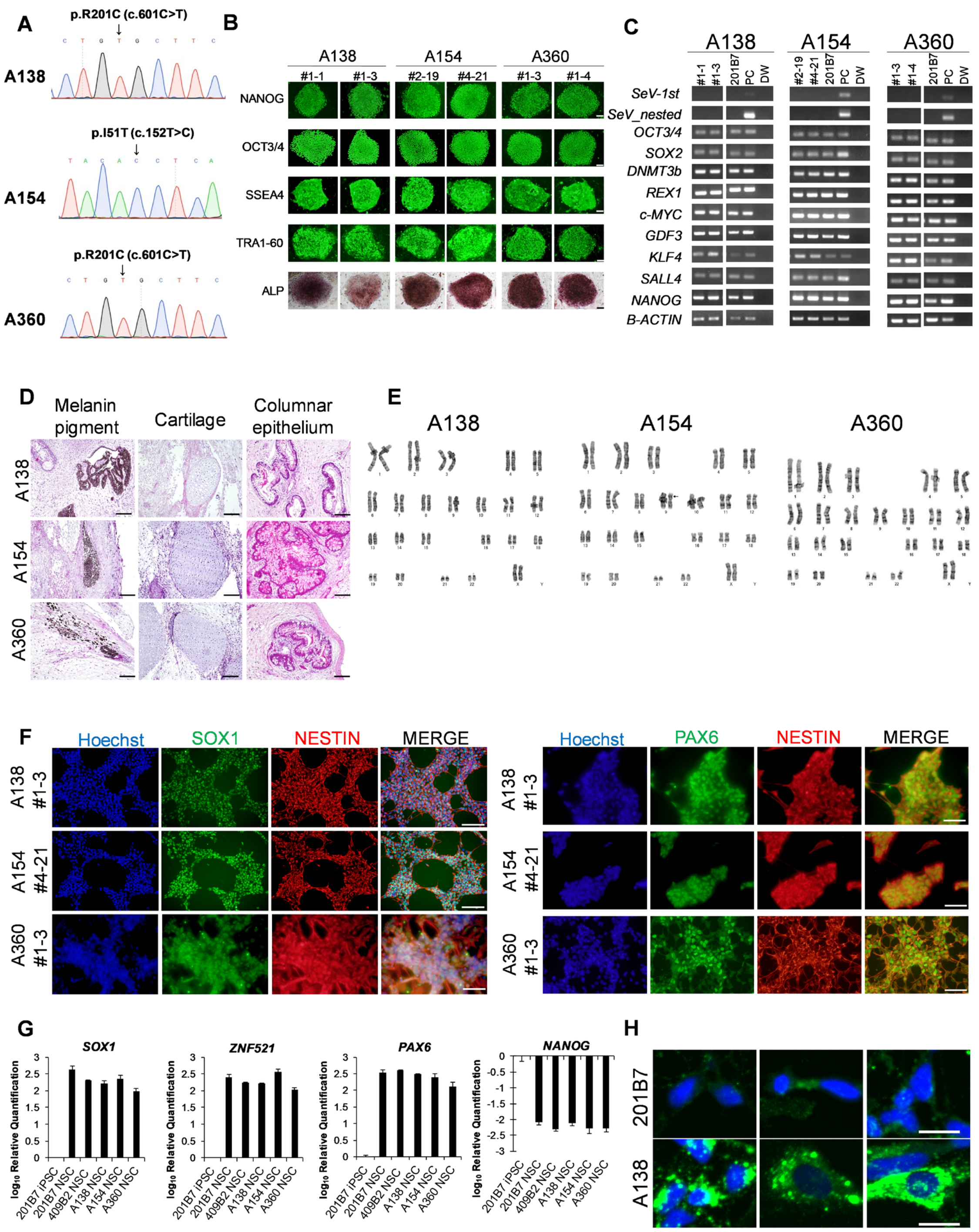

Figure S1. iPSCs and NSCs derived from patients with GM1 gangliosidosis (Related to Figure 1)

(A) Mutations of *GLB1* gene in the patients-derived fibroblasts were confirmed by direct sequencing. The *GLB1* gene was amplified by the genomic PCR and the resultant

PCR products were sequenced using ABI PRISMTM 310 Genetic Analyzer. (B) Immunofluorescence and Alkaline Phosphatase (ALP) staining of iPSC lines for pluripotency markers. The iPSC lines A138 #1-1, 1-3, A154 #2-19, #4-21, A360 #1-3 and #1-4 were generated from skin fibroblasts derived from GM1 gangliosidosis patients. Scale bars, 200 μm. (C) RT-PCR analyses of Sendai virus RNA and pluripotency markers. RT-PCR analyses of GM1 gangliosidosis-derived iPSCs for pluripotency markers. Primers used for *OCT3/4*, *SOX2*, *KLF4,* and *cMYC* were designed to detect the expressions of endogenous genes, but not of transgenes. To detect SeV RNA, nested RT-PCR was performed. PC: positive control, DW: Deionized water as a negative control. (D) Histological analyses of the iPSC-derived teratomas. The iPSC lines, A138 #1-3, A154 #4-21 and A360 #1-3, were transplanted into the testes of NOJ（NOD/Scid/Jak3 KO） immunodeficient mice. Palpable tumors were observed about 12-16 weeks after the transplantation. Tumor samples were collected, fixed in 10% formalin, and processed for paraffin-embedding and hematoxylin-eosin staining following standard procedures. Scale bars, 100 μm. (E) Karyotype analyses of the patients-derived iPSCs, A138 #1-3, A154 #4-21 and A360 #1-3. G band analyses of chromosome were performed by Nihon Gene Research Laboratories. Inc. (Sendai, Japan), according to the manufacturer’s protocol. (F) Immunofluorescence staining of the iPSC-derived NSCs for NSC markers. The A138 #1-3, A154 #4-21 and A360 #1-3 iPSC lines were induced for NSC differentiation. Scale bars, 100 μm. (G) Expression of NSC markers *SOX1*, *ZNF521*, *PAX6,* and *NANOG* in the iPSC-derived NSCs, measured by RT-qPCR. The bars represent the mean ± SD from three independent experiments and the ratio value is normalized to the levels in 201B7-iPSCs (Control iPSC). (H) Representative images with the high magnification corresponding to Figure 1B. Green: CTB, Blue: Hoechst. Scale bars, 200 μm.

kajihara *et al* **Figure S2**

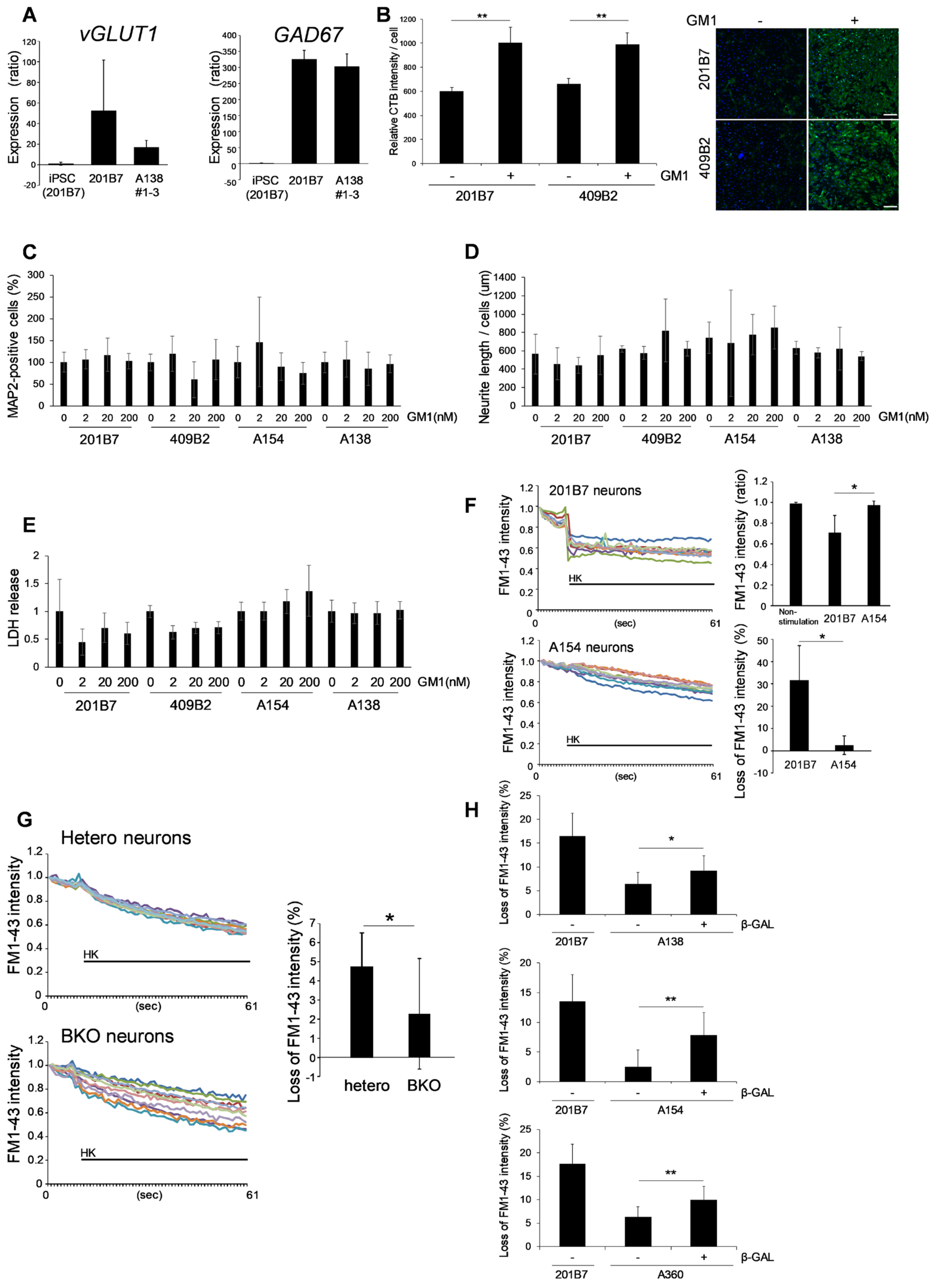

Figure S2. Deficit in presynaptic function of GM1 gangliosidosis neurons (Related to Figure 2)

No alteration in the number of MAP2-positive surviving cells or total neurite outgrowth in control- and disease-neurons with or without exposure to exogenous GM1 ganglioside. (A) Expression of glutamatergic (*vGLUT1*) and GABAergic (*GAD67*) neural markers was determined by qPCR (n = 3-5, n indicates the number of culture dishes in each condition). Data were ratio value normalized to the levels in 201B7-iPSCs. (B) Treatment of GM1 ganglioside in normal NSCs causes accumulation of GM1 ganglioside. Normal NSCs derived from 201B7 and 409B2 iPSCs were treated with 200 nM GM1 ganglioside for 72 hours. The NSCs were stained with AF488-CTB and the fluorescence intensities were measured using IN CELL ANALYZER 6000 (Left). $^{**}P < 0.01$ (n = 4, independent samples) Representative images (Right). Scale bars, 200 μm. (C-E) Immunostaining of MAP2 and LDH leakage assay using neurons at day 66 after the 20-day treatment with GM1 gangliosides (0, 2, 20, or 200 nM). (mean ± SD from three independent experiments). (C); MAP2-positive cells in control (201B7 and 409B2)- and disease (A138 #1-3 and A154 #4-21)-neurons with or without GM1 ganglioside application. Neuronal survival was shown as the relative value of MAP2-positive cells at each GM1 concentration using the non-treatment group for each cell line. (D); quantification data of neurite outgrowth in neurons with or without GM1 ganglioside exposure was also shown. (E); Relative enzymatic activity of lactate dehydrogenase (LDH) in the culture medium was assessed. (F) Decreased exocytotic release in the gangliosidosis-derived neurons. To examine exocytotic process, the loss of FM1-43 dye fluorescence was monitored. High potassium (HK^+) solution (50 mM) was applied for cell depolarization. A154 #4-21-disease neurons on Day 62. Each trace indicates a changed intensity of FM 1-43 fluorescence from single vesicle-like buttons. The decreased fluorescence intensity of FM dye was determined by comparing the stimulation intensity (2 sec after HK+ stimulation) with basal levels (2 sec before the stimulation). The quantitative data were shown as; (Upper) ratio value of basal/ HK+-stimulated one, and (Lower) loss of FM intensity [%: (basal intensity − HK+-stimulated one − fading value of fluorescence) × 100], respectively. $^{*}P < 0.05$ (n = 10, n indicates the number of randomly selected buttons). (G) Decreased exocytotic release in the BKO mice-derived neurons. To examine exocytotic process during the depolarization at the presynaptic sites, loss of FM1-43 dye fluorescence was monitored. HK^+ solution (50 mM) was applied for cell depolarization. Each trace indicates the changed intensity of FM 1-43 fluorescence from single vesicle-like buttons. The decreased fluorescence intensity of FM dye was determined by comparing the stimulation intensity (2 sec after HK+ stimulation) with basal levels (2 sec before the stimulation). The quantitative data are shown as loss of FM intensity [%: (basal

intensity − HK+-stimulated one − fading value of fluorescence) ×100]. * $P < 0.05$ ($n = 10$, n indicates the number of randomly selected buttons). Hetero neurons, neurons derived from β-GAL heterogeneous (*Glb1* +/-) mice; BKO neurons, neurons derived from β-GAL KO (*Glb1* -/-) mice. (H) Effects of β-GAL treatment on the neural cells. Improvement of the abnormal exocytotic release in the disease-derived neurons after the long-term treatments with 200 nM β-GAL. The neural cells were treated throughout the entire time course of neural differentiation. Left: A138 #1-3 on day 60. Middle: A154 #4-21 on day 87. Right: A360 #1-3 on day 62. *$P < 0.05$, **$P < 0.01$ ($n = 10$, mean ± SD).

kajihara *et al*   Figure S3

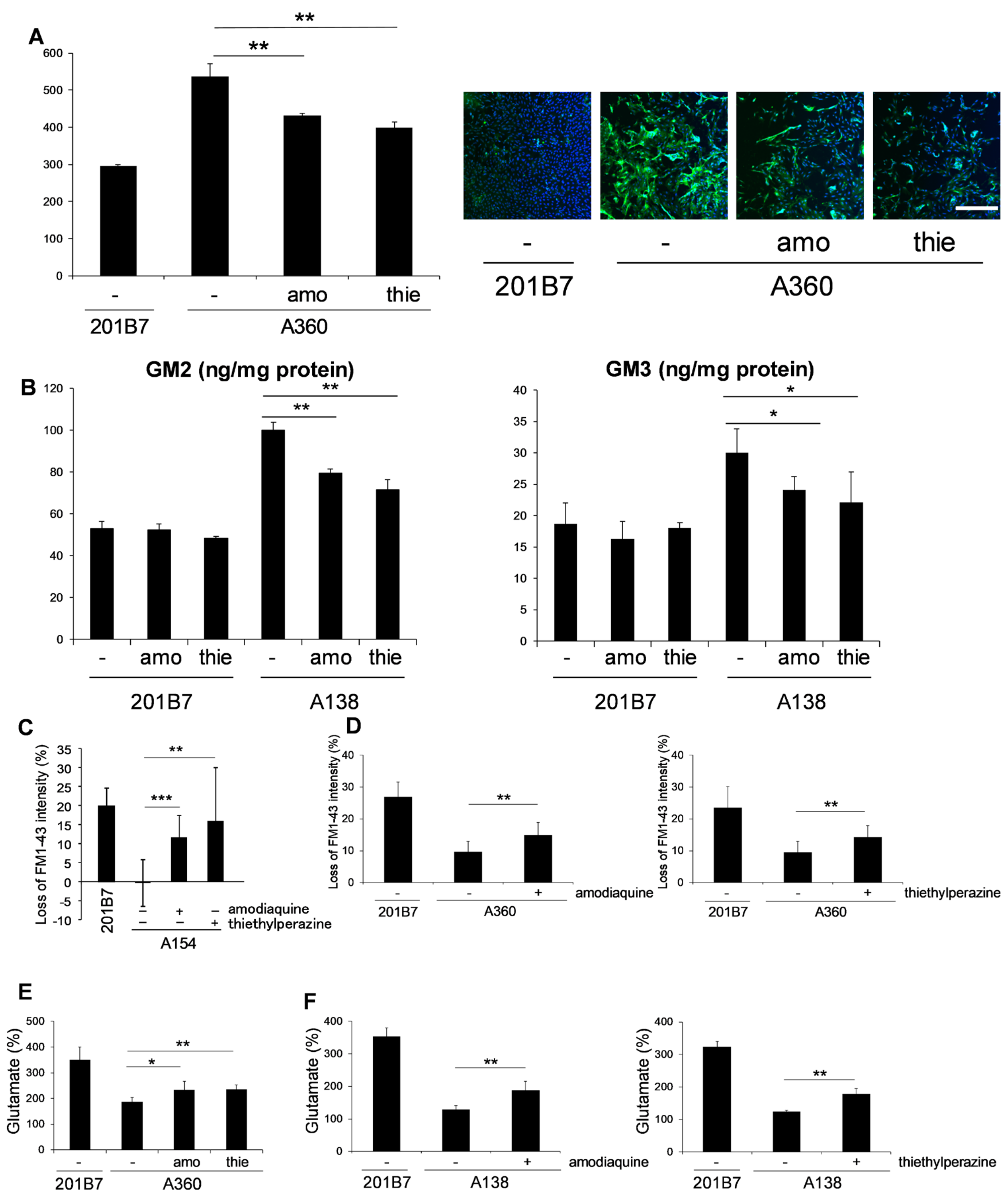

Figure S3. Gangliosides accumulation and in vitro presynaptic function is improved with the treatments of drug candidates (Related to Figure 3)

(A) 201B7 and A360 NSCs were treated with amodiaquine (5 μM) and thiethylperazine (5 μM) for 72 hours. The NSCs were stained with AF488-CTB and the fluorescence intensities were measured using IN CELL ANAYLZER 6000 (Left). $^{**}P < 0.01$ (n = 4, independent samples) Representative images (Right). Scale bars, 100 μm. (B)

Quantification of GM2 and GM3 gangliosides in the NSCs treated with amodiaquine and thiethylperazine. Gangliosides were purified from control (201B7)- and disease (A138 #1-3)-NSCs treated with amodiaquine (5 μM) and thiethylperazine (5 μM) and were measured by LC-MS (mean ± SD from three independent experiments). * $P < 0.05$, ** $P < 0.01$, indicated the treated versus non-treated NSCs, Student's t-test. (C-F) In vitro presynaptic function is restored with long-term treatments of the identified drug candidates. (C, D) Recovery from presynaptic dysfunction in the disease neurons after a long-term treatment with amodiaquine or thiethylperazine in vitro. These two compounds (1.0 μM, respectively) were applied throughout the entire time course of neural differentiation. ** $P < 0.01$, *** $P < 0.001$ (n = 10, n indicates the number of randomly selected buttons). (C); A154 #4-21 on day 74. (D); A360 #1-3 on day 62. (E, F) Treatment with amodiaqine or thiethylperazine restored the decrease of glutamate released from GM1 gangliosidosis-derived neurons. The amount of glutamate was measured by HPLC. The data were displayed as; ratio value (%) of the HK+-stimulated release/basal one, respectively. Basal release (1 min) was collected before the stimulation with HK^+ (50mM, 1 min). *P<0.05., **P<0.01 (n=4, n indicating the number of wells for each experimental condition). (E); A360 #1-3 on day 57. (F); A138 #1-3 on day 69

kajihara *et al*   Figure S4

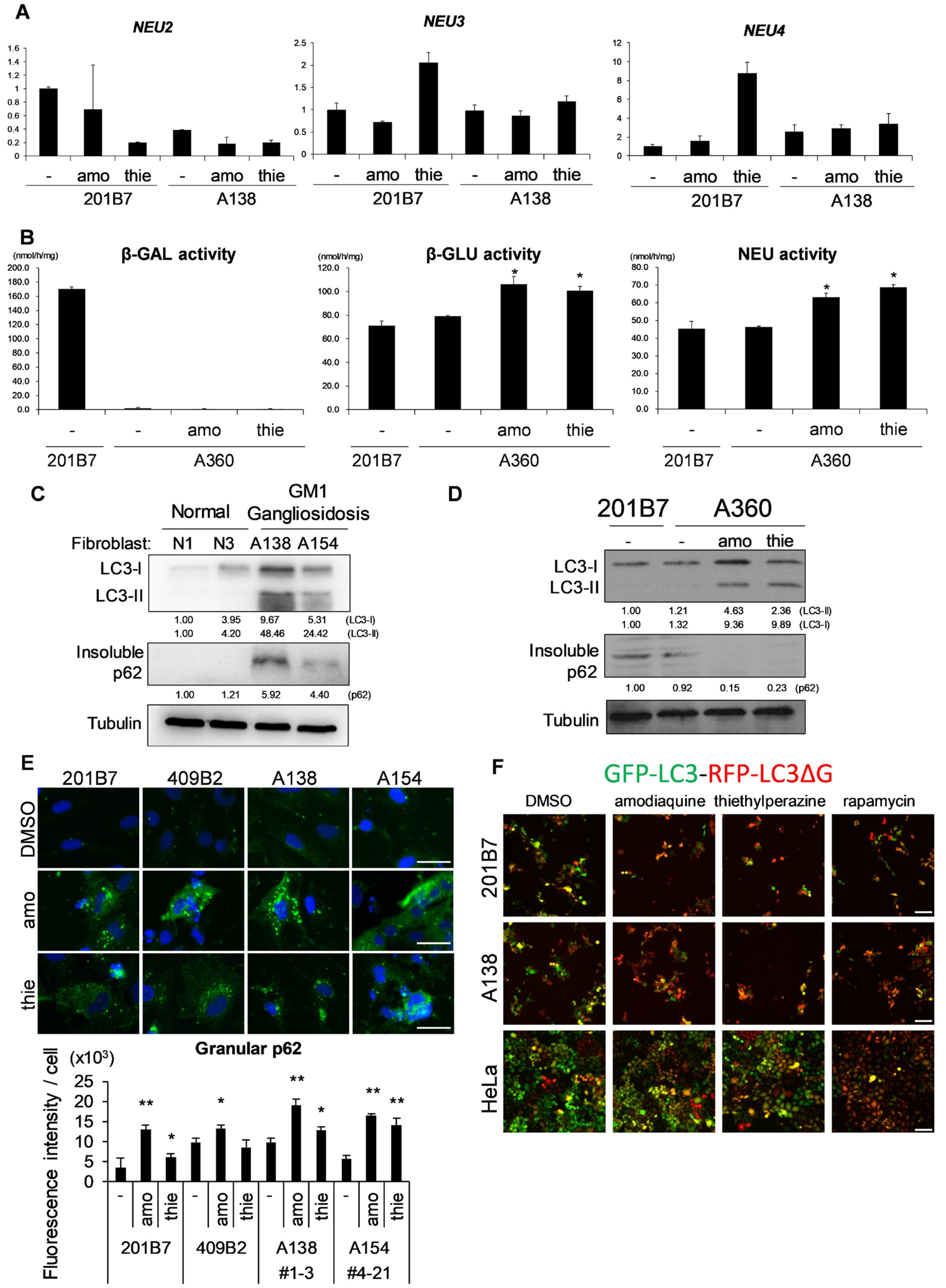

Figure S4. Amodiaquine and thiethylperazine enhance the lysosomal glycosphingolipid degradation and activate autophagy (Related to Figure 4)

(A) Gene expression of NEU family with drug candidate treatments. Normal (201B7) and disease-derived (A138 #1-3) NSCs were treated with 5 μM amodiaquine and 5 μM thiethylperazine for 72 hours. The expression levels of neuraminidases (*NEU2*, *NEU3* and *NEU4*) were measured by RT-qPCR. Data were ratio value normalized to the levels in 201B7-NSCs (-). (mean ± SD from three independent experiments). amo, amodiaquine; thie, thiethylperazine. (B) Enzyme activities of NEU1 and β-GLU were also enhanced with the candidate treatments. NSCs were treated with 5 μM amodiaquine (amo) and 5 μM thiethylperazine (thie) for 48 hours and enzyme activities of β-GAL (left), NEU1 (middle), and β-GLU (right) were measured. Enzyme activities are expressed as nmol/h/mg protein. (mean ± SD from three independent experiments). *$P < 0.05$, ** $P < 0.01$, indicated the treated versus non-treated NSCs, Student's t-test. (C-F) Autophagy in GM1 gangliosidosis-derived cells. (C) Autophagic flux was impaired in GM1 gangliosidosis-derived skin fibroblasts. The protein levels of LC3-II and insoluble p62 were enhanced in GM1 gangliosidosis-derived skin fibroblasts (A138 and A154) compared to normal fibroblasts (N1 and N3). The band intensities were measured using ImageJ software, normalized to each N1 control and described below the images. The results shown are representative of two independent experiments. (D) The protein levels of LC3-II were elevated and those of insoluble p62 decreased in NSCs treated with both compounds. NSCs were treated with 5 μM amodiaquine (amo) and 5 μM thiethylperazine (thie) for 72 hours, western blotting. The band intensities were measured using ImageJ software, normalized to the non-treated 201B7 and described below the images. (E) Granular p62 increased in NSCs treated with amodiaquine and thiethylperazine. NSCs were treated with the candidates as described in Figure 4F. Right: Granular p62 was measured by immunofluorescence staining for p62 followed by the quantification of the fluorescence intensity using IN CELL ANALYZER 6000. *$P < 0.05$, **$P < 0.01$, indicated the treated versus non-treated NSCs, Student's t-test. Left: Representative images. Scale bars, 200 μm. Green, p62, Blue, Hoechst. (F) Representative images corresponding to Figure 4G. Scale bars, 150 μm.

kajihara *et al*   Figure S5

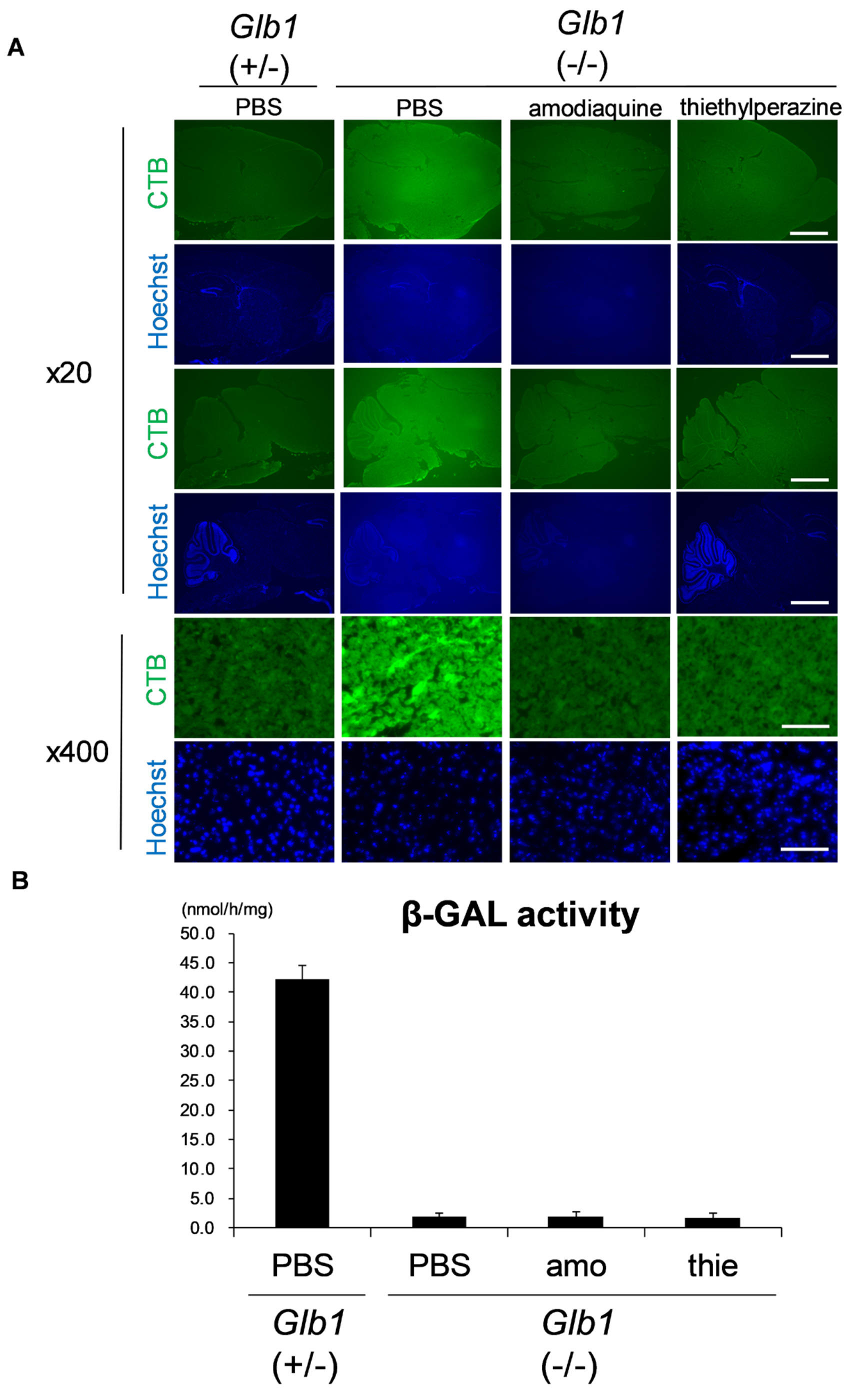

Figure S5. Treatments with amodiaquine and thiethylperazine can reduce GM1 ganglioside accumulations in the brains of model mice (Related to Figure 5)

(A) High and low magnified images corresponding to Figure 5A. Scale bars, 1000 μm (x20), 200 μm (x400). (B) Enzyme activities of β-GAL were not altered with the candidate treatments in *Glb1* KO mice, which were treated as described in Figure 5A.

The brain homogenates were prepared and enzyme activities of β-GAL were measured (mean ± SD from three independent experiments).

kajihara *et al*   Figure S6

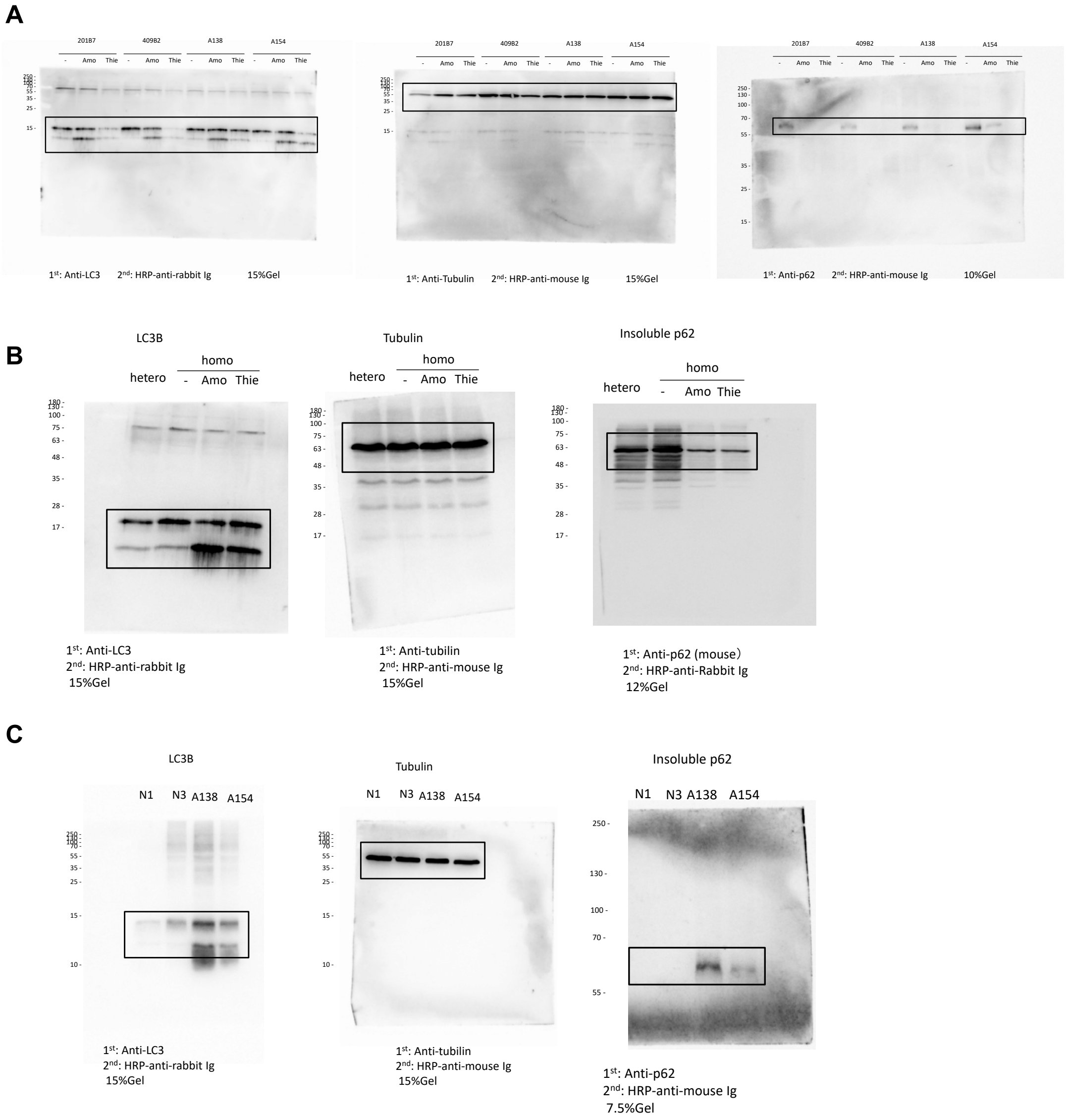

Figure S6. Uncropped images of immunoblots

Raw data corresponding to Figure 4F (**A**), to Figure 5C (**B**), and to Figure S4C (**C**).

Supplemental Experimental Procedure

Skin-derived fibroblasts

Fibroblasts were isolated from explants of skin biopsies obtained from patients following informed consent under protocols approved by the ethics committees of the assigning authors. Skin samples were minced and cultured in Dulbecco's modified essential medium (DMEM, Life Technologies) supplemented with 10% FBS. The subsequent fibroblast cultures were expanded for iPS cell induction as follows.

iPSC generation

We generated iPSCs from human skin-derived fibroblasts as described previously (Fusaki et al., 2009). Briefly, human fibroblast cells were seeded in six-well plates 1 day before infection with Sendai virus (SeV) vectors at 10 multiplicity of infection. After 7 days of culturing, the infected cells were harvested by trypsin and plated on 60-mm dishes coated with mitomycin C-treated mouse embryonic fibroblast feeder cells. From 18 to 25 days after infection, colonies were picked and cultured in human iPSC medium. To remove the Sendai virus, the culturing temperature was increased from 37℃ to 38℃ for 3 days at passage 1 or 2.

iPSC culture

Human iPSCs were cultured on the mitomycin C-treated mouse feeder cells in DMEM/F12 (SIGMA) supplemented with 20% KNOCKOUT serum replacement (KSR, Invitrogen), 2 mM L-glutamine (Life Technologies), 0.1 mM nonessential amino acids (NEAA, SIGMA), 0.1 mM 2-mercaptoethanol (SIGMA), 0.5% penicillin and streptomycin (Nacalai Tesque, Japan), and 5 ng/ml basic fibroblast growth factor (bFGF, WAKO, Japan).

RNA isolation and PCR

Total RNA was isolated using Sepasol® Super G reagent (Nacalai Tesque, Japan), and then transcribed to cDNA using Superscript III (Invitrogen) and random primers (Invitrogen). QuickTaq (TOYOBO, Japan) was used for RT-PCR and THUNDERBIRD qPCR Mix (TOYOBO) for RT-Quantitative PCR (RT-qPCR), as described previously (Soga et al., 2015), and data were analyzed using a StepOnePlus real-time PCR system (Applied Biosystems). Primers used for *OCT3/4*, *SOX2*, *KLF4* and c-*MYC* were designed to detect endogenous gene expression, but not that of transgenes. Nested RT-PCR was performed to detect the SeV genome. Table S1 lists the relevant primer sequences.

Table S1. Primers details for PCR, RT-PCR and RT-qPCR

Genes	Sequences (Forward; F, Reverse; R)	Application
GLB1 (I51T)	F: CTGGTGGACCTGGCTTAGCAATGGT<br>R: GCCAGTCCGGCTCATGACAAGCCCC	PCR
GLB1 (R201C)	F: TAGGAGGTAAACTTTTATGGTGAAT<br>R: ATGAAAACATGAAAAATCTCAATCT	PCR
SeV	F: GGATCACTAGGTGATATCGAGC<br>R: ACCAGACAAGAGTTTAAGAGATATGTATC	RT-PCR
SeV Nested	F: TCGAGCCATATGACAGCTCG<br>R: GAGATATGTATCCTTTTAAATTTTCTTGTCTTCTTG	RT-PCR(nested PCR)
OCT3/4	F: GACAGGGGGAGGGGAGGAGCTAGG<br>R: CTTCCCTCCAACCAGTTGCCCCAAAC	RT-PCR
SOX2	F: GGGAAATGGGAGGGGTGCAAAAGAGG<br>R: TTGCGTGAGTGTGGATGGGATTGGTG	RT-PCR
KLF4	F: GATTACGCGGGCTGCGGCAAAACCTACACA<br>R: TGATTGTAGTGCTTTCTGGCTGGGCTCC	RT-PCR
c-MYC	F: GCGTCCTGGGAAGGGAGATCCGGAGC<br>R: TTGAGGGGCATCGTCGCGGGAGGCTG	RT-PCR
NANOG	F: CAGCCCCGATTCTTCCACCAGTCCC<br>R: CGGAAGATTCCCAGTCGGGTTCACC	RT-PCR
GDF3	F: CTTATGCTACGTAAAGGAGCTGGG<br>R: GTGCCAACCCAGGTCCCGGAAGTT	RT-PCR
REX1	F: CAGATCCTAAACAGCTCGCAGAAT<br>R: GCGTACGCAAATTAAAGTCCAGA	RT-PCR
SALL4	F: AAACCCCAGCACATCAACTC<br>R: GTCATTCCCTGGGTGGTTC	RT-PCR
DNMT3b	F: TGCTGCTCACAGGGCCCGATACTTC<br>R: TCCTTTCGAGCTCAGTGCACCACAAAAC	RT-PCR
b-ACTIN	F: CAACCGCGAGAAGATGAC<br>R: AGGAAGGCTGGAAGAGTG	RT-PCR
PAX6	F: GTCCATCTTTGCTTGGGAAA<br>R: TAGCCAGGTTGCGAAGAACT	qPCR
SOX1	F: GCCCTGAGCCGACTGTGA<br>R: CCGTGAATACGATGAGTG	qPCR
ZNF 521	F: ACCTCCGTGTCCAGTACGAC<br>R: ATGTCAGGGGTTTGTTGAGC	qPCR
NANOG	F: CCAAAGGCAAACAACCCACTT<br>R: CGGGACCTTGTCTTCCTTTTT	qPCR
SYNAPTOTAGMIN I	F: TGAAGTACCTTTTGAACAAATCCA<br>R: GCATCGTTCTTGCCAATCTT	qPCR
SYNAPSIN I	F: TTGCCCAGATGGTTCGACTG<br>R: TGTCAACCTTGACCTTGCCC	qPCR
SYNTAXIN 1A	F: GAAGCACAGTGCCATCCTG<br>R: TGTTTGCTGTCTTCTTTATGTCG	qPCR
GM3 synthase	F: AAGAGACTGCCTTTGACATCC<br>R: CATATCCAAAACCCGCCAAAC	qPCR
GM2 synthase	F: AGAGGGTCAGGCAGATCTCA<br>R: TGCTGTGTTGGTCTGGTAGC	qPCR
GM1 synthase	F: CGCTATTCTTGCTGGGAGAG<br>R: GGGTCTTTAGGGTGAGGTTG	qPCR
GLB1	F: ACGGTGGACTTTGGAACAG<br>R: TGATTGTGGAGTGAGGTTGG	qPCR
HEXA	F: GCTATCGTGACCTGCTTTTCG<br>R: TCTCCAGTGTATGCCGTTTC	qPCR
NEU1	F: TTCTCCAACCCAGCACATC<br>R: ACTGTCTCTTTCCGCCATG	qPCR
NEU2	F: TGCGCAGAGGAGACTACGA<br>R: GTCTGCGCGTCATACAAGG	qPCR
NEU3	F: GGCTTGTTTGGGTGTTTGTT<br>R: TTTTGAATTGGCTTGGGTTC	qPCR
NEU4	F: ACCGCCGAGAGTGTTTTGG<br>R: CGTGGTCATCGCTGTAGAAGG	qPCR
GBA	F: ACAGGATTGCTTCTACTTCAGG<br>R: GGGTCAAAGGAGTCACAGTATG	qPCR
GALC	F: CCTGCATGAAGATGAGCTGTTCACA<br>R: TAGGTACTTGGGAAGGGCTGGGA	qPCR
GAPDH	F: GGCTGGCATTGCCCTCAACG<br>R: AGGGACTCCCCAGCAGTGAG	qPCR

Karyotype analysis

G band chromosome analyses were performed by Nihon Gene Research Laboratories Inc. (Sendai, Japan), according to the manufacturer's protocol.

Teratoma formation

The patient-derived iPSC lines grown on MEF feeder layers were collected by treatment with collagenase IV and injected into the testes of NOJ（NOD／Scid／Jak3 KO） immunodeficient mice (Okada et al., 2008). Palpable tumors were observed at 12-16 weeks after injection. Tumor samples were collected, fixed in 10% formalin, and processed for paraffin-embedding and hematoxylin-eosin staining following standard procedures.

Genomic Sequencing

The mutations of *GLB1* gene in the patients-derived fibroblasts were confirmed by direct sequencing. The genomic DNAs extracted were amplified by PCR and the resultant PCR products were sequenced by ABI PRISM^{TM} 310 Genetic Analyzer (Applied Biosystems) as described previously (Soga et al., 2015) .

Western blot analysis

Lysates were prepared in RIPA buffer (50 mM Tris-HCl [pH 8], 150 mM NaCl, 0.5% sodium deoxycholate, 0.1% SDS, and 1% Nonidet P-40) with freshly added protease inhibitors and phosphatase inhibitors. To detect insoluble p62, RIPA-insoluble pellets were dissolved in SDS sample buffer. Western blot analysis was performed as described previously (Kajihara et al., 2014). Briefly, samples were separated by SDS-PAGE and transferred to Immobilon-P membranes (Millipore), which were incubated for 1 h in blocking buffer (TBS containing 0.1% Tween 20 and 5% nonfat dry milk) followed by overnight incubation with primary antibody in blocking buffer. After extensive washing, the blot was incubated with a relevant secondary antibody for 1 h and processed using ECL reagents (GE Healthcare, Buckinghamshire, U.K.) according to the manufacturer's instructions. In order to compare the particular protein band intensities, the obtained scanned images were analyzed using ImageJ software (NIH, Bethesda, MA, USA). The relative band intensities were expressed in arbitrary units in relation to the appropriate reference sample band intensity. Antibodies and working conditions are listed in Table S2.

Table S2. Antibodies details for immunostaining and immunoblotting

Antibodies and other probes	Species	Working Dilution	Vendor (Catalog number)	Application
Anti-SSEA4	Mouse	1:500	MILLIPORE (MAB4304)	immunostaining
Anti-TRA-1-60	Mouse	1:500	MILLIPORE (MAB4360)	immunostaining
Anti-NANOG	Goat	1:1000	R&D systems (4903S)	immunostaining
Anti-OCT3/4	Mouse	1:500	Santa Cruz (sc-5279)	immunostaining
Anti-SOX1	Rabbit	1:100	Cell Signaling Technology	immunostaining
Anti-PAX6	Rabbit	1:1000	Covance (PRB-278P)	immunostaining
Anti-NESTIN	Mouse	1:500	R&D systems (MAB1259)	immunostaining
Cholera Toxin Subunit B (Recombinant), Alexa Fluor 488		1:2500	Molecular Probes (C34775)	immunostaining
Anti-LC3	Rabbit	1:500	Cell Signaling Technology	immunoblot
Anti-p62 (Human)	Mouse	1:500<br>1:200	MBL (M162-3)	immunoblot<br>immunostaining
Anti-p62 (Mouse)	Rabbit	1:1000	MBL (PM045)	immunoblot
Anti-MAP2	Rabbit	1:500	Millipore (Ab5622)	immunostaining
Anti-SYNAPSIN I	Mouse	1:500	Synaptic systems (106001)	immunostaining
Anti-mouse HRP	Goat	1:3000	Bio rad (170-6516)	immunoblot
Anti-Rabbit HRP	Goat	1:3000	Bio rad (170-6515)	immunoblot
Alexa 488-conjugated goat anti-mouse IgG	Goat	1:1000	Invitrogen (A11029)	immunostaining
Alexa 488-conjugated donkey anti-rabbit IgG	Donkey	1:1000	Invitrogen (A21206)	immunostaining
Alexa 594-conjugated goat anti-mouse IgG	Goat	1:1000	Invitrogen (A11005)	immunostaining
Hoechst 33258		1:2000	Invitrogen (H3569)	immunostaining

Cell staining and immunocytochemistry

Alkaline phosphatase staining was performed using the Leukocyte Alkaline Phosphatase kit (SIGMA). For immunocytochemistry, cells were fixed with 4% paraformaldehyde in phosphate-buffered saline (PBS) for 30 min at 4℃, and then treated with 0.2% Triton X-100 for 15 min at room temperature (RT) to localize nuclear proteins. The cells were then washed three times with blocking buffer (PBS containing 2% FBS) and incubated overnight at 4℃ with primary antibodies diluted in blocking buffer. The bound primary antibodies were detected by incubation in Alexa-Fluor-conjugated secondary antibodies for 1 h, and nuclei were counterstained with 1 μg/ml Hoechst 33258 (Invitrogen). Table S2 lists the antibodies and conditions used in these experiments.

Neuralization of iPSCs

We differentiated iPSCs into neural stem cells (NSCs) using Gibco PSC neural induction medium (Life Technologies) according to the manufacturer's protocol with minor modification (Yan et al., 2013). Semi-confluent iPSCs cultured on MEF feeder cells were detached with collagenase IV and re-plated onto Geltrex (Life Technologies)-coated plates with Neural Induction Medium [NIM, consisting of Neurobasal Medium (NM: from Life Technologies) and 1/100 volume of Neural Induction Supplement (NIS: Life Technologies)], containing Y-27632 (10 μM). Medium was changed with fresh NIM (without Y-27632) on days 3 and 5 after plating. At day 7, colonies of differentiated NSCs were re-plated for further maintenance (48 hours), before detachment using Accutase (Life Technologies) and gentle pipetting to dissociate the colonies into single cells. The dissociated NSCs were then cultured on Geltrex-coated dishes with Neural Expansion Medium (NEM) comprising 50% Advanced DMEM/F12 (Life Technologies), 50% NM, and 1/100 volume of NIS, supplemented with Y-27632 [10 μM, until passage 2 (P2)]. The NSCs were then passaged every 5-9 days and used for neural differentiation up to P10. For neuralization, the NSCs were dissociated for plating on polyethyleneimine (PEI)-coated glass-bottom dishes (Matsunami Glass Ind.) or astrocyte feeders prepared from mouse cortex with NM containing a 1/50 volume of B-27 supplement (Life Technologies) and 0.5 mM L-glutamate. Three weeks later, the culture medium was replaced with DMEM containing 20% KSR, and fresh medium was replaced once or twice a week. For immunocytochemistry and imaging assays, we used neurons maintained for 56-85 days from neuralization. In some experiments, recombinant human β-GAL (R&D systems, 200nM) was added to the culture medium during neuralization

FM 1-43 imaging

FM 1-43 imaging was used to monitor exocytotic activity in the iPSC-derived neurons, as described previously (Numakawa et al., 1999). Briefly, the neuralizing culture medium was replaced with artificial cerebrospinal fluid (ACSF; 125 mM NaCl, 2.5 mM KCl, 2 mM $CaCl_2$, 1 mM $MgCl_2$, 1.25 mM NaH_2PO_4, 25 mM $NaHCO_3$, 25 mM glucose and 5 μM FM 1-43) for 30 min at 37℃. After several washes with fresh ACSF without the FM dye, the FM 1-43 concentration in cells was monitored using a fluorescence microscope (DMi8, Leica). Neurons were irradiated with an excitation wavelength of 488 nm and emitted fluorescence was detected at 520 nm. HK+ (50 mM KCl) ACSF solution was used to trigger membrane depolarization, and decreases in the cellular FM 1-43 fluorescence intensity were determined by comparing the stimulation (2 sec after HK+ stimulation) and basal (2 sec before the stimulation) intensities. The intensity of FM emission was captured every 1 sec and stored as stacked images using the LAS X imaging system (Leica). To confirm reproducibility, the imaging experiment was carried out at least 3-5 times on independent cultures.

Measurement of lysosomal enzyme activities

Lysosomal enzyme activities in cell lysates were measured fluorometrically with 4-methylumbelliferone (4MU) substrates including 4-methylumbelliferyl-β-D-galactopyranoside (Sigma-Aldrich) for β-GAL, 4-methylumbelliferyl-α-D-N-acetylneuraminic acid (Sigma-Aldrich) for NEU1, and 4-methylumbelliferyl-β-D-glucopyranoside (Sigma-Aldrich) for β-GLU, as described previously (Okumiya et al., 2006). Briefly, 10 μl of the cell homogenate was added to 60 μl of the 4MU substrate solution in a 96-well, white microtiter plate (OptiPlate-96, Packard, Meriden, CT). The reaction mixture was then incubated at 37°C for 1 h, and the reaction was stopped by addition of 200 μl of 0.2M Glycine-NaOH buffer pH10.7 with 0.1% Triton X-100. Fluorescence intensities were measured with a fluorescence plate reader (excitation 360 nm; emission 450 nm; Infinite F200 Pro, TECAN Japan, Kawasaki, Japan), and corrected for substrate blank without cell homogenates. To calibrate the measurement of liberated 4MU, we used 125 μmol/l of 4MU in 20 mmol/l sodium phosphate buffer at pH 7.0 instead of the sample. The enzyme activity was expressed as nanomoles of 4MU released per hour per milligram cellular protein (nmol/h/mg protein).

Ganglioside purification and quantification by LC-MS

Gangliosides were extracted according to the procedure developed by Svennerholm and

Fredman (Fong et al., 2009). Briefly, cells or portions of mice brains were resuspended in water, lysed by freeze thawing, and then extracted twice with chloroform/methanol/water (4:8:3 v/v/v). The upper layers were pooled, and distilled water was added to give a final chloroform/methanol/water ratio of 1:2:1.4 (v/v/v), before centrifuging for 30 min at 2000 x g. Additional methanol (half of the lower phase volume) and 0.01 M KCl (20% of the total volume of the lower phase plus added methanol) were added to the lower phase, vortexed, and then centrifuged for 30 min at 2000 x g. The two upper phases were combined and evaporated to dryness in a rotary evaporator. The dried residue was dissolved in 2 ml of KCl 0.1 M, and recycled 3 times on a Sepak C18 column (1 ml). The bound material was eluted with 15 ml of methanol and 5 ml of chloroform/methanol (2:1 v/v). The eluates were evaporated to dryness in a rotary evaporator, and resuspended in 50% methanol as samples for HPLC analysis using an Agilent 1100 series HPLC system (Santa Clara, CA) comprising a binary pump, degasser, column heater (60°C), and refrigerated auto-sampler (5°C). Samples or standards (10 μl) were separated using an APS-2 hypersil hydrophilic column (150 mm x 2.1 mm, 3 μm, Thermo Scientific, Waltham, MA) coupled to an APS-2 guard column (10 mm x 2.1 mm inner diameter) with an acetonitrile/ammonium acetate buffer gradient. Eluants from the HPLC system were introduced into an Agilent 6470 Triple Quadrupole Mass Spectrometer to quantify each class of ganglioside.

Neuronal survival assay and neurite length measurement

NSC lines (201B7, 409B2, A138 #1-3, A154 #4-21) were plated at 1.6 x 10^4 cells/well in PEI-coated 96-well plates, and then cultured for neuronal differentiation for 66 days. GM1 exposure was performed during day 46 to 66, and then again at day 96. At day 66, cells were fixed with 4% paraformaldehyde for 20 min at room temperature. After blocking and permeabilization with PBS containing 1% BSA and 0.1% Triton X-100 for 30 min, cells were incubated with anti-MAP2 antibody (1:500, ab5622, Millipore) at 4°C overnight. Cells were then washed with PBS and stained with Alexa Fluor 488 donkey anti-rabbit IgG (1:1000, Life Technologies) and Hoechst 33342 (1 μg/ml, Invitrogen) for 1 hour at room temperature. Fluorescence of MAP2 and nuclear staining was visualized using an IN Cell Analyzer 6000, and quantified as number of MAP2-positive cells per field and length of MAP2-positive neurites using Developer Toolbox software. To assess cell viability, neuronal survival was shown as the relative value of MAP2-positive cells at each GM1 concentration compared to the non-treatment group for each cell line.

Treatment of GM1 gangliosidosis model mice with hit compounds

Glb1 gene KO mice (BKO mice) were bred and kept in specific pathogen-free conditions in the Center for Animal Resources and Development (CARD), Kumamoto University. PBS, 40 mg/kg amodiaquine, or 6 mg/kg thiethylperazine was intraperitoneally injected into homozygous BKO mice twice a day from 9 to 15 days of age (n = 4 each). At 15 days of age, the mice were sacrificed and the brain samples were collected for further experiments. For histological analysis of GM1-ganglioside storage, frozen sections of the brain samples were fixed for 10 min in 4% paraformaldehyde in PBS, pH 7.4 at room temperature. After blocking with 1% BSA in PBS for 10 min at room temperature, sections were incubated for 1 h at room temperature with Alexa Fluor 488-conjugated cholera toxin B in PBS, (1:200; Molecular Probes). After 3 × 10-min washes in PBS they were counterstained with Hoechst 33258.

Primary culture of mouse neurons

Cerebral cortical tissues of 1- or 2-day-old controls (*Glb1* +/-) and BKO mice (*Glb1* -/-) were dissected for culturing cortical neurons as previously reported (Numakawa et al., 2009). Briefly, dissected brain tissue was digested with 0.25% trypsin at 37° C for 30 min, and then cortical cells were dissociated by gentle pipetting, followed by plating on polyethyleneimine-coated glass-bottom dishes at 5 x 10^5 cells/cm^2. The cortical cell cultures were maintained for 22 days with neurobasal media containing B27 prior to FM imaging.

Measurement of autophagic flux

To generate stable cell lines expressing an autophagic flux probe (GFP-LC3-RFP-LC3ΔG), NSCs and HeLa cells were retrovirally transfected with pMRX-IP-GFP-LC3-RFP-LC3ΔG (Addgene, 84572) as previously described (Kaizuka et al., 2016). The cells were plated at 5 x 10^4 cells/well in 96-well plates coated with Geltrex and then treated with 5 μM amodiaquine, 5 μM thiethylperazine or 1 μM rapamycin for 72 hours. The fluorescence intensities of GFP and RFP were measured using IN Cell Analyzer 6000 (GE Healthcare). The data was analyzed with Developer Toolbox software (GE Healthcare). Autophagic flux was estimated by calculating the GFP/RFP signal ratio.

LDH leakage assay

In order to evaluate the possible cytotoxicity of GM1 ganglioside, lactate dehydrogenase (LDH) leaking from the neurons into the culture medium was measured

by Cytotoxicity LDH Assay Kit-WST (Dojindo) according to the manufacturer's protocol with minor modification. Following exposure to 200nM GM1 ganglioside during day 46 to 66 of neuronal differentiations, 50 µl culture medium was transferred from 96 well culture plate to a clear 96 well plate. After adding 50 µl Working Solution to each wells, plate was incubated for 30 min at the room temperature. Then 25 µl Stop Solution was applied to each wells and the absorbance at 450 nm was measured by a microplate reader.

Measurement of glutamate

The amount of released glutamate, an excitatory neurotransmitter, from differentiated neurons was measured with high performance liquid chromatography (HPLC; Shimazu Co, Osaka, Japan). First, neuronal cultures were gently washed 4-6 times using assay buffer (ACSF; 125 mM NaCl, 2.5 mM KCl, 2 mM $CaCl_2$, 1 mM $MgCl_2$, 1.25 mM $NaH2PO_4$, 25 mM $NaHCO_3$, and 25 mM glucose). Then, fresh assay buffer was applied for 1min and collected without a stimulation as the basal release of glutamate. Afterword, secondary incubation with assay buffer containing KCl (50 mM) was performed for 1 min. Before the analysis with HPLC, amino acids in the collected samples were derivated with o-phthalaldehyde and -2-mercaptoethanol. The 12- or 24-well plate were used for cell cultures to carry out the determination of glutamate.

Supplemental References

Fong, B., Norris, C., Lowe, E., and McJarrow, P. (2009). Liquid chromatography-high-resolution mass spectrometry for quantitative analysis of gangliosides. Lipids *44*, 867-874.

Fusaki, N., Ban, H., Nishiyama, A., Saeki, K., and Hasegawa, M. (2009). Efficient induction of transgene-free human pluripotent stem cells using a vector based on Sendai virus, an RNA virus that does not integrate into the host genome. Proceedings of the Japan Academy Series B, Physical and biological sciences *85*, 348-362.

Kaizuka, T., Morishita, H., Hama, Y., Tsukamoto, S., Matsui, T., Toyota, Y., Kodama, A., Ishihara, T., Mizushima, T., and Mizushima, N. (2016). An Autophagic Flux Probe that Releases an Internal Control. Mol Cell *64*, 835-849.

Kajihara, R., Sakamoto, H., Tanabe, K., Takemoto, K., Tasaki, M., Ando, Y., and Inui, S. (2014). Protein phosphatase 6 controls BCR-induced apoptosis of WEHI-231 cells by regulating ubiquitination of Bcl-xL. Journal of immunology (Baltimore, Md : 1950) *192*, 5720-5729.

Numakawa, T., Kumamaru, E., Adachi, N., Yagasaki, Y., Izumi, A., and Kunugi, H. (2009). Glucocorticoid receptor interaction with TrkB promotes BDNF-triggered PLC-gamma signaling for glutamate release via a glutamate transporter. Proc Natl Acad Sci U S A *106*, 647-652.

Numakawa, T., Takei, N., Yamagishi, S., Sakai, N., and Hatanaka, H. (1999). Neurotrophin-elicited short-term glutamate release from cultured cerebellar granule neurons. Brain Res *842*, 431-438.

Okada, S., Harada, H., Ito, T., Saito, T., and Suzu, S. (2008). Early development of human hematopoietic and acquired immune systems in new born NOD/Scid/Jak3null mice intrahepatic engrafted with cord blood-derived CD34 + cells. International journal of hematology *88*, 476-482.

Okumiya, T., Keulemans, J.L., Kroos, M.A., Van der Beek, N.M., Boer, M.A., Takeuchi, H., Van Diggelen, O.P., and Reuser, A.J. (2006). A new diagnostic assay for glycogen storage disease type II in mixed leukocytes. Mol Genet Metab *88*, 22-28.

Soga, M., Ishitsuka, Y., Hamasaki, M., Yoneda, K., Furuya, H., Matsuo, M., Ihn, H., Fusaki, N., Nakamura, K., Nakagata, N*., et al.* (2015). HPGCD outperforms HPBCD as a potential treatment for Niemann-Pick disease type C during disease modeling with iPS cells. Stem Cells *33*, 1075-1088.

Yan, Y., Shin, S., Jha, B.S., Liu, Q., Sheng, J., Li, F., Zhan, M., Davis, J., Bharti, K., Zeng, X*., et al.* (2013). Efficient and rapid derivation of primitive neural stem cells and generation of brain subtype neurons from human pluripotent stem cells. Stem Cells Transl Med *2*, 862-870.
